# Information-flow interfaces

**DOI:** 10.1007/s10703-024-00447-0

**Published:** 2024-05-23

**Authors:** Ezio Bartocci, Thomas Ferrère, Thomas A. Henzinger, Dejan Nickovic, Ana Oliveira da Costa

**Affiliations:** 1 TU Wien, Vienna, Austria; 2 Imagination Technologies, Kings Langley, UK; 3 IST, Klosterneuburg, Austria; 4 AIT, Vienna, Austria

**Keywords:** Contract-based design, Interface theory, Hyperproperties, Information-flow

## Abstract

Contract-based design is a promising methodology for taming the complexity of developing sophisticated systems. A formal contract distinguishes between *assumptions*, which are constraints that the designer of a component puts on the environments in which the component can be used safely, and *guarantees*, which are promises that the designer asks from the team that implements the component. A theory of formal contracts can be formalized as an *interface theory*, which supports the composition and refinement of both assumptions and guarantees. Although there is a rich landscape of contract-based design methods that address functional and extra-functional properties, we present the first interface theory designed to ensure system-wide security properties. Our framework provides a refinement relation and a composition operation that support both incremental design and independent implementability. We develop our theory for both *stateless* and *stateful* interfaces. Additionally, we introduce information-flow contracts where *assumptions* and *guarantees* are sets of flow relations. We use these contracts to illustrate how to enrich information-flow interfaces with a semantic view. We illustrate the applicability of our framework with two examples inspired by the automotive domain.

## Introduction

The rise of pervasive information and communication technologies seen in cyber-physical systems [1], internet of things, and blockchain services has been accompanied by a tremendous growth in the size and complexity of systems [2]. Subtle dependencies involving multiple architectural layers and unforeseen environmental interactions can expose these systems to cyber-attacks. This problem is further exacerbated by the heterogeneous nature of their constituent components, which are often developed independently by different teams or providers. In such a scenario, defining and enforcing security requirements across components at an early stage of the design process becomes a necessity. This engineering approach is called *security-by-design*. Although in recent years there has been impressive progress in the verification of security properties for individual system components, the science of compositional security design [3, 4] is still in its infancy.

Security policies are usually enforced during design by restricting the flow of information in a system [5]. *Information-flow policies* define which information a user or a software/hardware component is allowed to observe or to interfere with while interacting with another component. The design of systems that satisfy information flow requirements is mainly supported today by *verification* methods. Verification of information flow properties is a well-studied problem with a rich landscape of both theory and tools, ranging from language-based [6–9] to simulation-based [10] approaches. Existing verification methods do not exploit the component-based structure of complex systems. First, a single verification tool cannot be used in presence of heterogeneous components. Second, there are no guidelines on how to divide-and-conquer the verification effort. More specifically, it is not clear what are the properties to assess in individual components that collectively uphold the system-level information flow policy. In addition, there is no obvious process of combining verification outcomes from these components to infer system-level properties. It follows that there is a need for a rigorous and compositional design method that takes information flow into account from the early stages of system development and that complements and facilitates the subsequent verification effort.

From a formal-language perspective, information flow is a *hyperproperty* [11] characterized over sets of system executions. Enforcing information flow policies is challenging due to the presence of side channels and implicit flows, which can potentially breach information security measures. For example, in a modern car, the tight coupling between the cyber and the physical components may allow an attacker to infer computational properties, such as secrets used for encryption, from side-channels, such as power consumption and electromagnetic radiation [12]. The increasing connectivity in the automotive systems facilitates attacks that involve gathering data from a car, exploiting weaknesses of its software implementation and ultimately gaining control over it [13, 14].

In this paper, we present a *contract-based design* [15] approach for information-flow policies. Contract-based design provides a formal framework for building complex systems from individual components, mixing both top-down and bottom-up steps. A top-down step decomposes and refines system-wide requirements; a bottom-up step assembles a system by combining available components. A formal contract distinguishes between *assumptions*, which are constraints that the designer of a component puts on the environments in which the component can be used safely, and *guarantees*, which are promises that the designer asks from the team that implements the component. A theory of formal contracts can be formalized as an *interface theory*, which supports the composition and refinement of both assumptions and guarantees [16–18]. While there is a rich landscape of interface theories for functional and extra-functional properties [19–22], we present the first interface theory that is designed for ensuring system-wide security properties, thus paving the way for a science of safety and security co-engineering.

Our information flow interface theory allows to talk about the flow of information between system variables while abstracting away on the concrete semantics of how the information flows. This focus on the structural aspects of information flow enables compositional reasoning in presence of heterogeneous components and is complementary to the existing body of work on information flow verification. For example, various heterogeneous components can be implemented under different semantics. However, if the component flows can be derived from its implementation under its concrete semantics, our theory needs only to know what these flows are but is agnostic about the underlying semantic interpretation. This enables compositional design of secure systems from trusted components and allows deriving necessary component properties from system-level requirements, thus providing a divide-and-conquer approach to the verification tasks.

Our theory is based on information-flow assumptions as well as information-flow guarantees. As an interface theory, our theory supports both *incremental design* and *independent implementability* [17]. Incremental design allows the composition of different system parts, each coming with their own assumptions and guarantees, without requiring additional knowledge of the overall design context. Independent implementability enables the separate refinement of different system parts by different teams that, without gaining additional information about each other’s design choices, can still be certain that their designs, once combined, preserve the specified system-wide requirements. While in previous interface theories, the environment of a component is held responsible for meeting assumptions, and the implementation of the component for the guarantees, there are cases of information-flow violations for which blame cannot be assigned uniquely to the implementation or the environment. In information-flow interfaces we therefore introduce, besides assumptions and (open system) guarantees, a new, third type of constraint—called *closed-guarantees*—whose enforcement is the shared responsibility of the implementation and the environment.

We develop our framework for both *stateless* and *stateful* interfaces. Stateless information-flow interfaces are built from primitive information-flow constraints—assumptions, open-guarantees, and closed-guarantees—of the form “the value of a variable *x* is always independent of the value of another variable *y*.” Stateful information-flow interfaces add a temporal dimension, e.g., “the value of *y* is independent of *x* *until* the value of *z* is independent of *x*.” The temporal dimension is introduced through a natural notion of state and state transition for interfaces, not through logical operators. We prove that our calculus of information-flow interfaces satisfies the principles of incremental design and independent implementability.

*Contribution* This paper expands and further improves our preliminary manuscript published at FASE 2022 [1] with the following new contributions:

- We introduce a natural semantic interpretation for information-flow interfaces (See Sect. 3.3) as contracts where assumptions and guarantees are sets of flow relations (i.e., implementations and environments).
- We add a new example where we show the application of both stateless and stateful information-flow interfaces to the top-down and bottom-up design of an electronic vehicle immobilizer (See Sect. 2.3).
- We provide all the detailed proofs of our theory and extend the discussion on the related work.
- We introduce a semantic definition of complement-flow composition in Definition 3 and prove that it is equivalent to the syntactic expression presented in the conference paper (c.f., Lemma 2), highlighting the role of the syntactic definition as a general proof method for compositional properties on information-flow interfaces.

*Paper organization* Section 2 presents two examples from the automotive industry motivating the applicability of our theory with two examples from the automotive industry. Section 3 introduces the notion of stateless interface and component algebra for secure information flows, while Sect. 4 extends this theory with stateful components and interfaces. Section 5 discusses the related work and we conclude in Sect. 6.

## Application examples

We motivate our approach with two examples from the automotive industry.

**Fig. 1:**
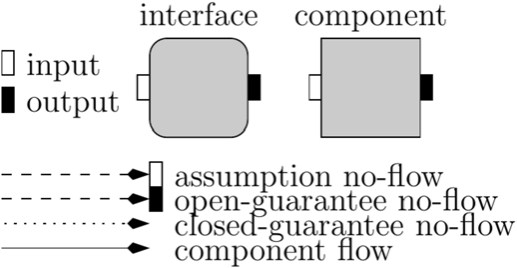
Representation of the objects in our theory

We adopt the graphical representation depicted in Fig. 1 to represent the different entities of our interface theory. Interfaces specify no-flow requirements and are meant to be used as a design or specification formalism, while components abstract concrete implementations by describing their flow of information. We use a square to denote a *component* and a square with rounded edges to denote an *interface*. During our target system’s successive design and refinement steps, we need to reason about its subsystems. Subsystems are typically *open systems*, interacting with an external environment by receiving inputs from their environment and reacting to them by producing appropriate outputs. We make a visual distinction between *inputs* and *outputs* by depicting them with a white and a black rectangle associated to an interface or a component, respectively.

Interfaces specify restrictions on information flows, which we call no-flow relations and we depict them with dashed and dotted arrows. Information-flow interfaces distinguish between environment and implementations responsibilities by specifying three kinds of requirements: an *assumption* on its environment, an *open-guarantee* on its implementations, and a *closed-guarantee* on the closed-system defined by composing its environment and implementations. Dashed arrows to input variables represent assumptions, while dashed arrows to output variables represent open-guarantees. Closed-system no-flows requirements (closed-guarantees) are represented as dotted arrows to output variables. In contrast, components specify implemented information flows, which we depict by solid-line arrows.

### Shared communication infrastructure

Our first example is a shared communication infrastructure (also referred to as a *shared bus*) in a car that connects the distance warners and a wheel sensor to the breaking system and the odometer. Distance warners sense the car’s proximity to other objects and send their analysis to other components. In our example, we have two distance warners, one at the front and another at the back of the car, using the shared bus to communicate with the braking system. The wheel sensor senses the wheel rotations and sends this information through the shared bus to the odometer. This example is an adaptation of the industrial case study presented by Marcus Mikulcak et al. in [10]. In this case study, the authors focused on the verification task, while in our example, we illustrate how to use our interface theory to do the stepwise design of such infrastructure.

**Fig. 2:**
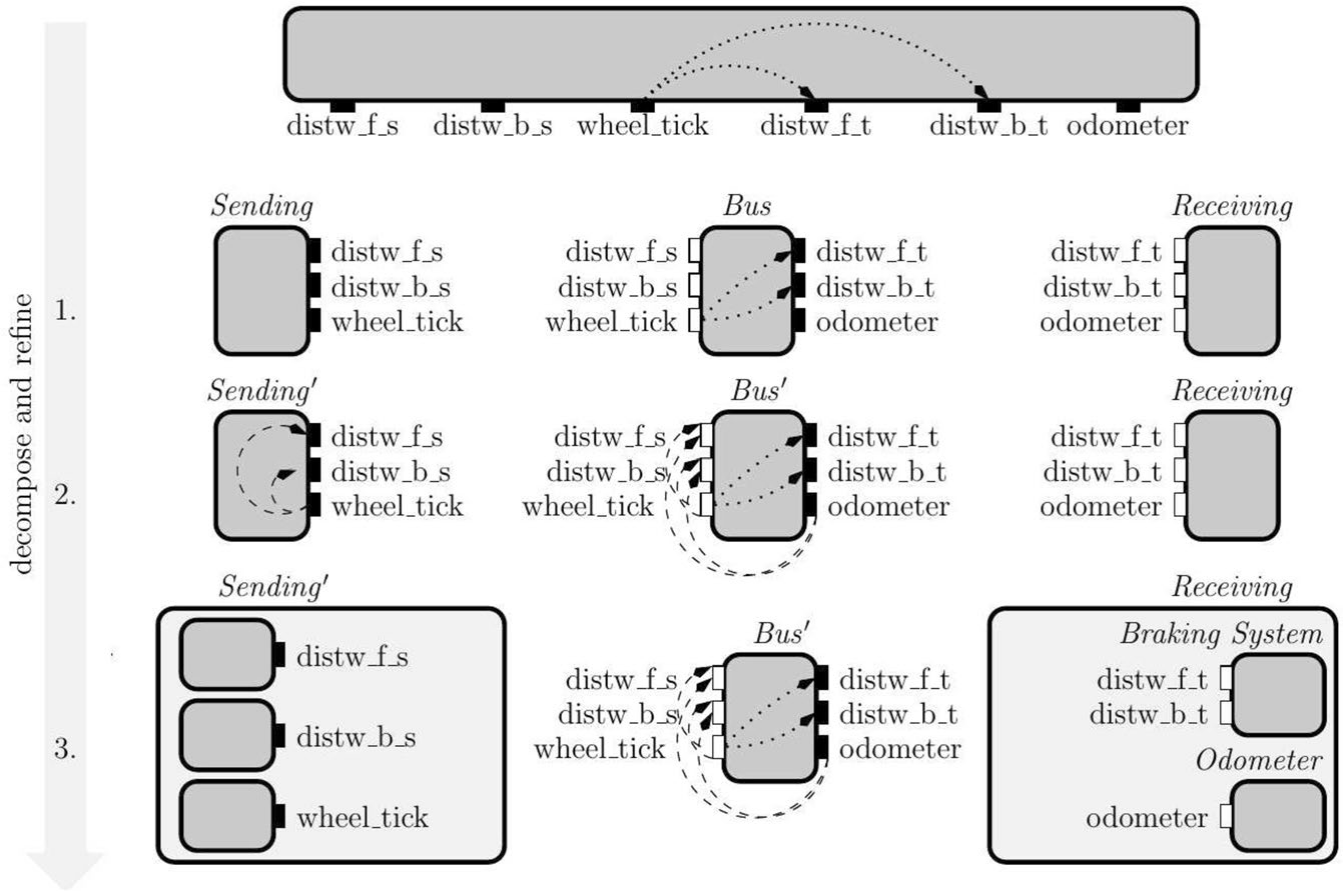
Top-down design of a shared communication infrastructure used by two distance warners, *distw_f_s* and *distw_b_s*, and a wheel sensor, *wheel_tick*, to communicate with the braking system, *distw_f_t* and *distw_b_t*, and the odometer, *odometer*, respectively

The main goal of our design process is to guarantee the following system-level requirement: *“information from the wheel sensor does not flow to the braking system”*. In other words, we require that the communication channel between the distance warners and the braking system cannot be interfered with (e.g., by the wheel sensor data) because it performs a safety-critical functionality. This requirement is an example of an *integrity policy*. We enforce this integrity policy using our secure-by-design theory by propagating appropriate requirements to subsystems and components through successive decomposition and refinement steps, shown in Fig. 2.

**Top-down design.** We start the design process with the specification of our system-level integrity policy. This policy is formalized in the form of two closed-guarantees depicted in the topmost interface in Fig. 2. These closed-guarantees forbid flow of data from the wheel sensor *wheel_tick* to target of the distance warners, *distw_f_t* and *distw_b_t*.

Next, we naturally decompose the initial system into three subsystems: the *sending subsystem* (warners and wheel sensor), the *shared bus*, and the *receiving subsystem* (breaking system and odometer). The decomposition of the system into subsystems is a manual step performed by the engineer, in which an interface is associated to each subsystem. Despite the decomposition being a manual activity, our interface theory provides support to automatically check whether the system is decomposed in a way that preserves the overall security requirements. In our first attempt (step 1 in Fig. 2), we propose a straight-forward decomposition of the original requirement: we keep the two closed-guarantees from the first interface as closed-guarantees in the *Bus* interface and do not add further assumptions and guarantees in all three interfaces. However, in this decomposition, the *Bus* interface is not *well-formed* – it misses assumptions and open-guarantees that are required to support its closed guarantees. We illustrate this issue with an example. For instance, the *Bus* interface allows a flow from *wheel_tick* to the source of the front distance warner through its environment which, together with the flow from the distance warner source to its target allowed by *Bus* open-guarantee, defines a flow from *wheel_tick* to *distw_f_t* forbidden by the interface’s closed-guarantee.

To remedy the well-formdness problem, we can use open-guarantees instead of closed-guarantees to specify the forbidden flows in the *Bus* interface. However, this is not sufficient – in that case the composition of the sub-system interfaces does not refine the system-level requirement. In fact, our interface theory tells us that we must add (1) new assumptions to the *Bus* interface, and (2) new open-guarantees to the *Sending* interface that imply the added *Bus* interface assumptions. With these additions, shown in the step 2 of Fig. 2, the composition of the three interfaces is now a refinement of the original requirement.

With this certified decomposition of the original specification, our theory guarantees that each subsystem can now be further *refined independently* (possibly by different teams). The step 3 of Fig. 2 illustrates an independent refinement of the Sending and the Receiving interfaces.

**Fig. 3:**
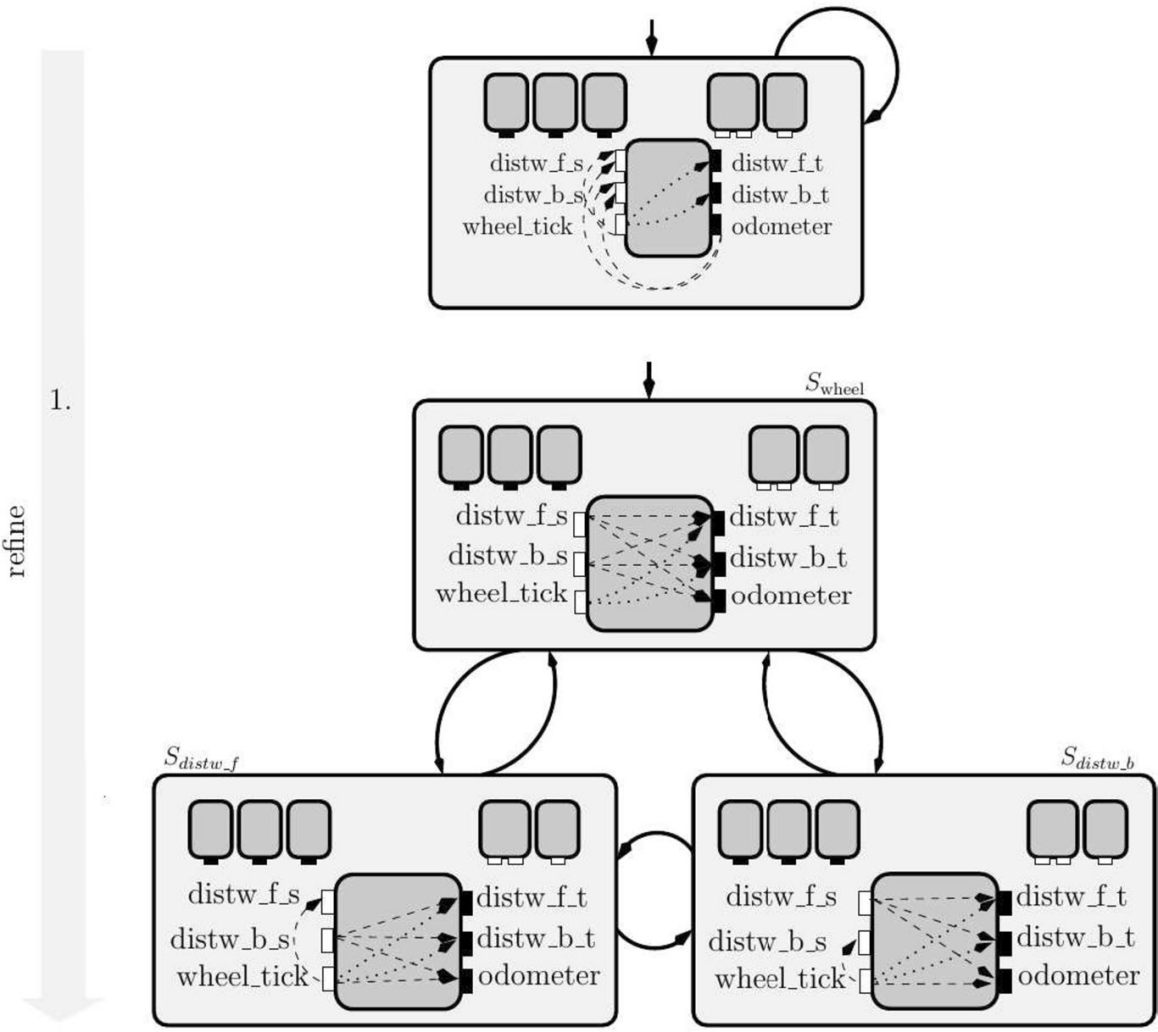
Design of mutually exclusive shared communication infrastructure for distance warners and the wheel odometer. Each state is defined by the composition of the interfaces inside

**Stateful top-down design.** In Fig. 3, we present the stateful view of the system, requiring the system to satisfy the composition of the Sending, the Bus, and the Receiving interfaces derived in Fig. 2 at all times. We refine the initial interface to specify that in each specification state only one of the sending components can use the bus.

The interfaces that define each state are named after the sending component that can use the bus (e.g. in the state $S_{\text {wheel}}$ only the $wheel\_tick$ can use it). All three states have the same closed-guarantee requiring no flows from the wheel tick to the distance warners’ target. For each state, we specify the mutually exclusive use by one of the sending components by adding to the open-guarantees the requirement that there is no flow from the inputs of the other sending components to any of the bus outputs. In particular, the open-guarantee in the interface of the state $S_{\text {wheel}}$ requires there is no flow from distance warners to any of the output variables.

As the access to the bus is mutually exclusive, we can simplify the assumptions on the environment in the Bus interface while keeping our interfaces well-formed. Recall that closed-guarantees (our primary constraint for well-formedness) specify information-flow restrictions on the interaction between environments and implementations. With fewer implementations (the open-guarantee imposes more flow restrictions), we can accept more environments (we need fewer flow restrictions on the assumption) and still meet the open-guarantee requirements. In this example, we simplify the assumptions as follows: $S_{\text {wheel}}$ has an empty assumption, while the states specifying the exclusive use of the bus by one of the distance warners only require from the environment that there is no flow from the wheel tick to the distance warner that is allowed to use the bus.

Finally, the components of our system can be, for instance, the Simulink and Stateflow models provided to the authors [10] by their industrial partners. We can then use the tool introduced in their work to verify whether these components implement the stateful interfaces we derived.

In summary, our framework defines relations on both stateless and stateful interfaces specifying information-flow policies that allow to check if: (i) a given interface refines (or abstracts) the current specification; (ii) two interfaces are compatible for composition; (iii) a specification is consistent; (iv) information-flows in a component define an implementation of a given interface; and (v) a system decomposition refines the system specification.

### Electronic vehicle immobilizer

Our second example considers an *electronic vehicle immobilizer* (EVI) that is a security device handling a transponder key. Car manufacturers use it to prevent hot wiring a car, thus its theft [14, 23]. If the transponder authentication fails, then the engine control unit blocks the car’s ignition. The communication between the immobilizer and other components in the vehicle takes place through the controller area network (CAN), a serial communication technology that is commonly used in automobile architectures to connect electronic control units (ECUs). This communication protocol does not include native support of security-related features. Thus, it is the responsibility of the components that use the bus to enforce confidentiality and integrity policies.

**Fig. 4:**
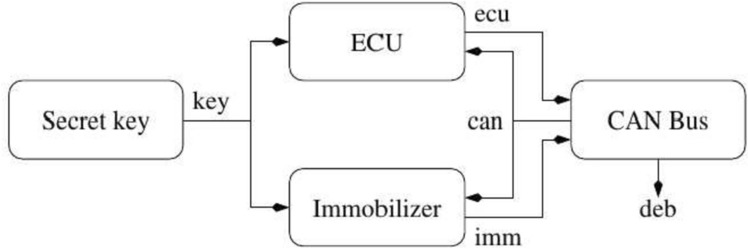
High-level view on immobilizer feature requirements

In this example, we illustrate how the main concepts of our interface theory can help in both the top-down and the bottom-up design of the EVI security requirements. We model the communication of an automotive engine control with an immobilizer through the CAN, which is adapted from the architecture described by Lemke [23]. The authentication follows a challenge-response protocol. The communication session starts with the engine’s ECU sending a freshly generated random number encrypted with a secret key known to both of the devices. The immobilizer replies to this challenge with an appropriate response encrypted with the same key. Figure 4 shows a high-level overview of our model which must enforce this security property: *the secret key shall never leak to the environment via the CAN bus*. More specifically, we depict the shared key as the key variable between the ECU and the immobilizer; the communication using the CAN bus with variables ecu and imm connecting the ECU and immobilizer to the CAN, and the variable can broadcasting the CAN output to them; and deb as a debug variable outputting from the CAN. We now illustrate how to use our framework to implement this property in (1) a top-down, and (2) a bottom-up design fashion.

**Fig. 5:**
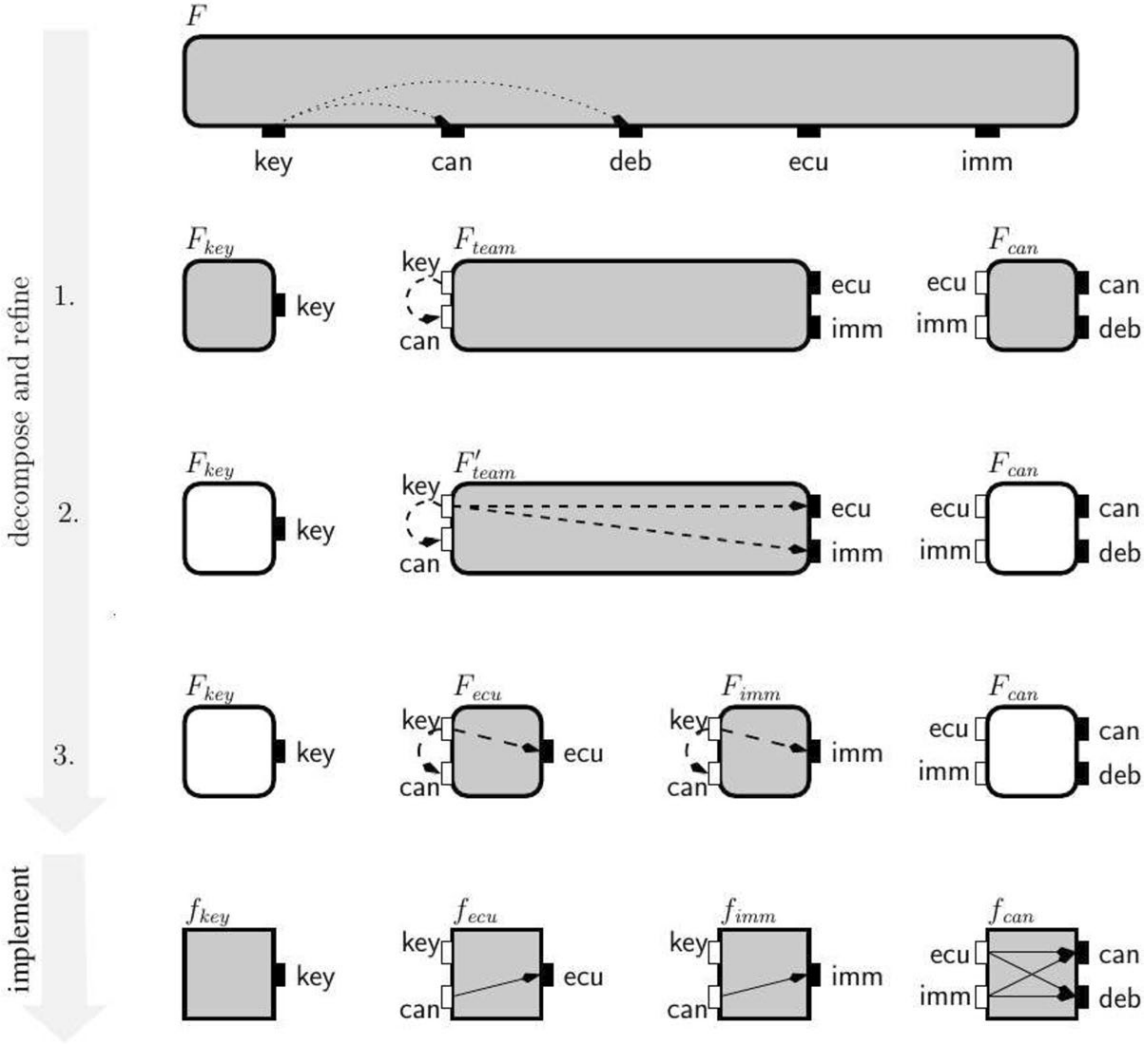
Top-down design for EVI system with key for drawing interfaces and components

**Stateless top-down design.** We first demonstrate stepwise refinement of a global specification: different engineering teams can independently implement subsystems, without violating the overall specification. We start by illustrating this process for the stateless case in Fig. 5. We start with the interface $F$ that represents the overall (closed) system when it is in operation mode. It specifies the global property that information from key is not allowed to flow to can or deb. We assume that the secret key and the CAN bus are standard components provided by third-party suppliers. Our goal is to design the remaining sub-system consisting of the immobilizer and the ECU. This gives us a natural decomposition of $F$ into three interfaces: (1) $F_{key }$ specifying the secret key, (2) $F_{can }$ specifying the CAN bus, and (3) $F_{team }$ specifying the sub-system that we want to further develop.

The decomposition of a system into subsystems is the responsibility of the design team. Our framework provides tools for checking whether the subsystems are compatible and whether their composition is a refinement of the original system. After the first step of Fig. 5, showing an apparent decomposition of the original specification, the closed-guarantee from $F$ becomes an assumption in $F_{team }$. This decomposition fails the checks provided by our framework, because $F_{team }$ *is not compatible* with $F_{can }$—their composition would violate the assumption from $F_{team }$ by enabling the secret key to flow to the CAN via the ECU or the immobilizer. These two interfaces can be made compatible by strengthening open-guarantees of $F_{team }$ and forbidding the key to flow to the ECU and the immobilizer, resulting in the interface $F_{team }'$. We then further decompose $F_{team }'$ into two interfaces: (1) $F_{ecu }$ specifying the ECU component, and (2) $F_{imm }$ specifying the immobilizer component. We note that the composition of $F_{key }$, $F_{can }$, $F_{ecu }$ and $F_{imm }$ refines the original system-level specification $F$. Finally, we implement the four interfaces, derived from the overall specification, in components $f_{key }$, $f_{ecu }$, $f_{imm }$ and $f_{can }$. We note that the implementation of $f_{ecu }$ and $f_{imm }$ could be done independently, by two separate teams. The ECU (immobilizer) component guarantees that the secret key does not flow to its output port and works correctly in any environment that forbids other means of the secret key flowing to the CAN bus.

**Fig. 6:**
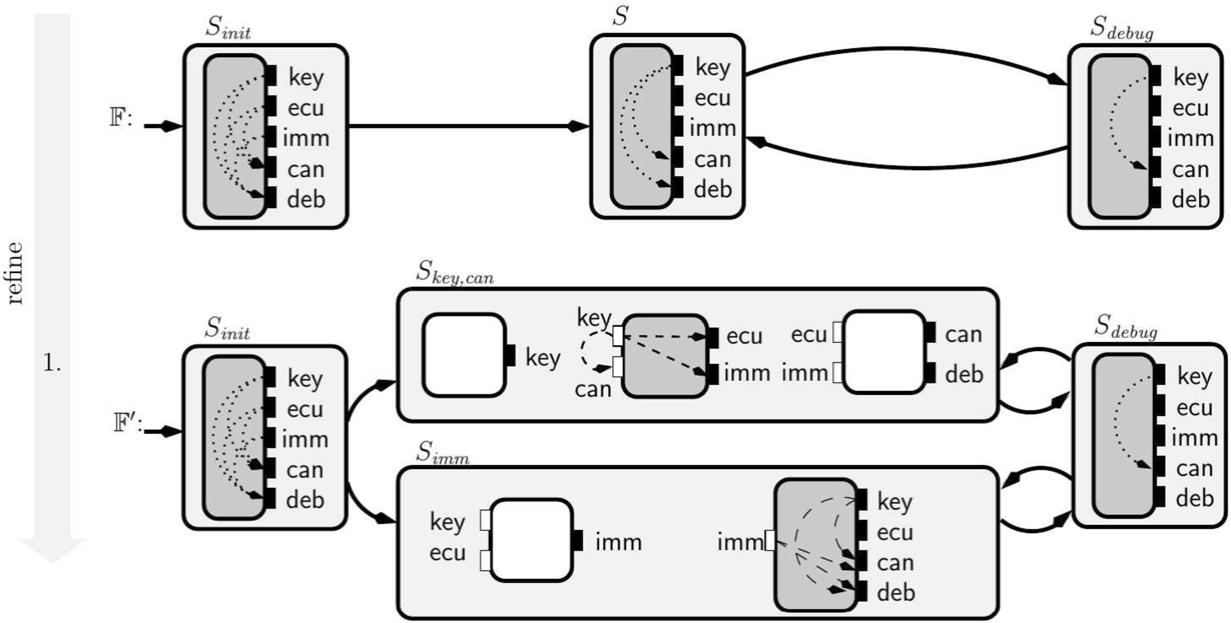
Stateful design for EVI system

**Stateful top-down design.** The system being designed can be in one of the following modes: *initialization*, *operational* or *debug*. This is illustrated in the stateful interface ${\mathbb {F}}$ in Fig. 6. *Stateful interfaces* are finite state machines with every state being labeled with a stateless interface. During initialization no information flows to CAN or the debug port, this state ends when an immobilizer communicates with the car. Then, the operation mode is decorated with the same interface $F$ from the stateless example while the initialization is decorated with the interface $S_{init }$ in Fig. 6. Debug mode specified in interface $S_{debug }$ allows all information to flow to the debug port.

In the stateful interface ${\mathbb {F}}'$ we illustrate a refinement of the initial specification in ${\mathbb {F}}$. We consider the case that the team needs to accommodate two different architectures for the operational mode, for example due to backward compatibility constraints. Then, in addition to the decomposition of $F$ showcased in Fig. 5, an alternative is specified in which the immobilizer is a third-party part.

**Fig. 7:**
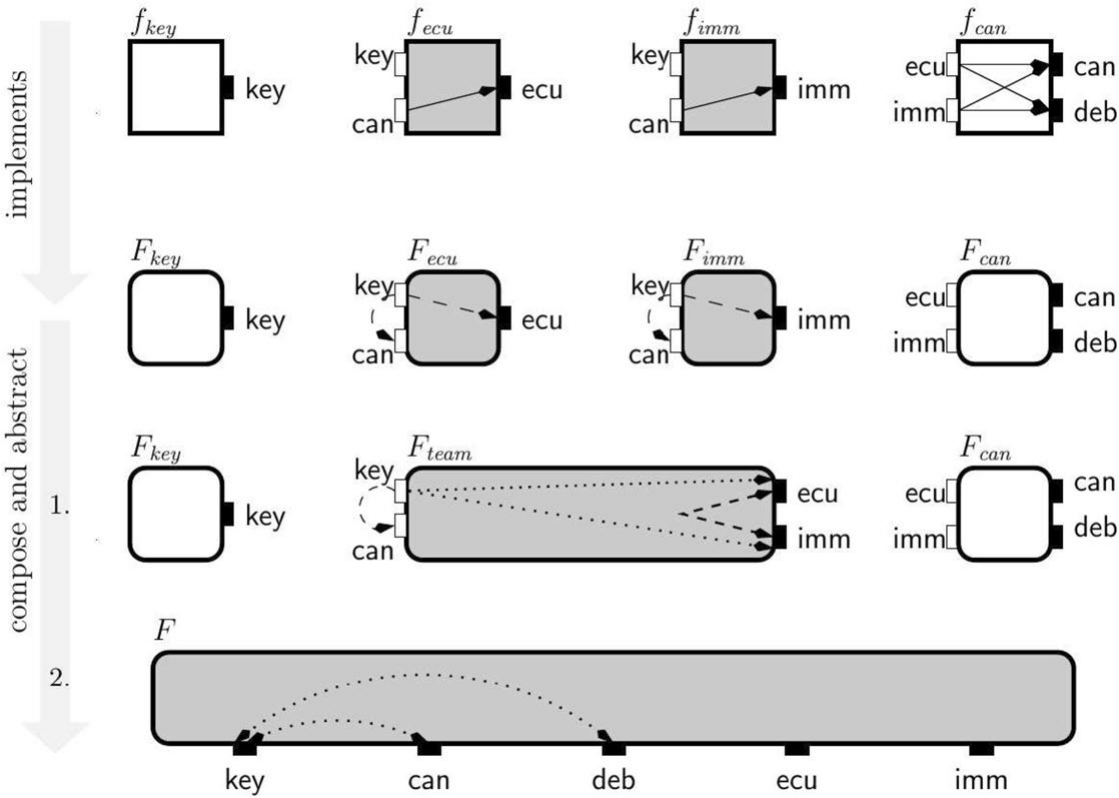
Bottom-up design for EVI system

**Stateless bottom-up design.** The bottom-up design process, illustrated in Fig. 7, is to a large extent symmetric to the top-down approach. We start with the available secret key, ECU, immobilizer and CAN components, specified by interfaces $F_{key }$, $F_{ecu }$, $F_{imm }$ and $F_{can }$. The main design step consists in composing a set of interfaces and *inferring new global closed-guarantees of the composition*. These closed-guarantees are flows that cannot be created given the current set of assumptions and open-guarantees. In the example we infer two global closed-guarantees, that the secret key can never flow either to the CAN or to the debug port, because those ports can only be accessed through $\textsf {ecu}$ or $\textsf {imm}$ ports to which $\textsf {key}$ cannot flow.

## Stateless information-flow interfaces

This section introduces a stateless interface and component algebra for secure information flow. In particular, we consider the *structural* properties of information flow within a system. In other words, we are interested in the existence or absence of an information flow between variables rather than specifying how the flow happens.

An *information-flow interface* specifies forbidden information flows in an open system by defining three types of constraints: assumption, open-guarantee and closed-guarantee. An interface *assumption* characterizes flows that are not allowed in the system environment. While an *open-guarantee* describes all flows forbidden in the open system defined by the interface, i.e., flow restrictions local to the interface’s implementations. The *closed-guarantee* qualifies flows not allowed at the interaction between the system and its environment. They specify a requirement on the closed system (environment with implementations) to be enforced by a combination of the open-guarantee and assumption no-flows requirements.

Information-flow interfaces explicitly distinguish between variables owned by the environment from variables owned by the implementation, referred to as *input* and *output* variables. Variables ownership establishes whose entity is responsible for enforcing restrictions on the flow of information to each of the system’s variables. We require input and output variables to define disjoint sets to ensure a clear-cut on the responsibility to enforce such policies.

We introduce *flow relations*, as both reflexive and transitively closed relations, to represent information flow between variables. An *information-flow component* abstracts the implementation of a system by a *flow relation*.

### Definition 1

Let $X$ and $Y$ be disjoint sets of *input* and *output* variables, respectively, with $Z= X\cup Y$ the set of all variables. A relation ${\mathcal {M}}\subseteq Z\times Y$ is a *flow relation* iff it is a transitive relation, and reflexive over $Y\times Y$. A * stateless information-flow component* is a tuple $(X,Y,{\mathcal {M}})$ where ${\mathcal {M}}\subseteq Z\times Y$ is a flow relation, called *flows*. A *stateless information-flow interface* is a tuple $(X,Y,{\mathcal {A}},{\mathcal {G}}, {\mathcal {P}})$ where ${\mathcal {A}}\subseteq Z\times X$ is a relation, called *assumption*; ${\mathcal {G}}\subseteq Z\times Y$ is a relation, called *open-guarantee*; and ${\mathcal {P}}\subseteq Z\times Y$ is a relation, called *closed-guarantee*.

We say that a *component implements* a given interface when it does not have flows forbidden by the interface’s open-guarantee. Likewise, a component is a *permissible environment* of a given interface when it does not have flows forbidden by the interface’s assumption. While implementations of an interface $F$ have the same sets of input and output variables as $F$, permissible environments have the same sets but with their roles switched.

### Definition 2

Let $F= (X, Y, {\mathcal {A}}, {\mathcal {G}}, {\mathcal {P}})$ be an information-flow interface. A component $f_{{\mathcal {E}}} = (Y, X, {\mathcal {E}})$ is an *environment* of $F$. We say that $f_{{\mathcal {E}}}$ is a *permissible environment of* $F$, denoted $f_{{\mathcal {E}}} \models _{\!\!{\mathcal {A}}} F$, iff ${\mathcal {E}}\subseteq \overline{{\mathcal {A}}}$, where $\overline{{\mathcal {A}}}= (Z\times X) {\setminus } {\mathcal {A}}$. A component $f= (X, Y, {\mathcal {M}})$ *implements* the interface $F$, denoted $f\models _{{\mathcal {G}}} F$, iff ${\mathcal {M}}\subseteq \overline{{\mathcal {G}}}$, where $\overline{{\mathcal {G}}}= (Z\times Y) {\setminus } {\mathcal {G}}$.

We observe that, as intuitively explained before, closed-guarantees do not influence the definition of implementations and permissible environment. However, they play a pivotal role in defining well-formed interfaces introduced next.

An information-flow interface is *well-formed* when it has at least one implementation and one permissible environment. Therefore, all of its relations must be irreflexive. We refer to irreflexive relations as *no-flow* relations. In this work, we are interested in interfaces defined over no-flow relations. Note that, by definition, a variable always has access to its value. Hence, no implementation can satisfy an interface requiring no flow of information from a variable to itself.

A well-formed interface ensures, additionally, that its closed-guarantee is *consistent* with its open-guarantee and assumption. A closed-guarantee is inconsistent (for a given open-guarantee and assumption) when there exists an environment permissible by the assumption and an implementation allowed by the open-guarantee that, when composed, includes a flow forbidden by the closed-guarantee. Given two flow relations ${\mathcal {M}}$ and ${\mathcal {M}}'$, their composition is the transitive closure of all their flows, i.e., $({\mathcal {M}}\cup {\mathcal {M}}')^*$. To formalize consistency for the closed-guarantee of a given interface $F$, we define next the *set of all flows defined by the composition* ${\mathcal {N}}\bullet \mathcal {N}'$ *of two no-flow relations* ${\mathcal {N}}$ and $\mathcal {N}'$, *which we naturally define as the pairwise composition of all flow relations disjoint from one of the relations being composed.*

### Definition 3

The *set of flows defined by the complement-flow composition of no-flow relations* ${\mathcal {N}}\subseteq U\times V$ and ${\mathcal {N}}' \subseteq U' \times V'$ is:

$$\begin{aligned} \begin{aligned} {\mathcal {N}}\bullet \mathcal {N}'&= \{(z,z')\in ({\mathcal {M}}\cup {\mathcal {M}}')^*\ \mid \ {\mathcal {M}}\subseteq {\overline{{\mathcal {N}}}}, {\mathcal {M}}' \subseteq \overline{{\mathcal {N}}'}, \\&\quad \text { and }{\mathcal {M}}\text { and }{\mathcal {M}}' \text {are flow relations}\}. \end{aligned} \end{aligned}$$

**Fig. 8:**
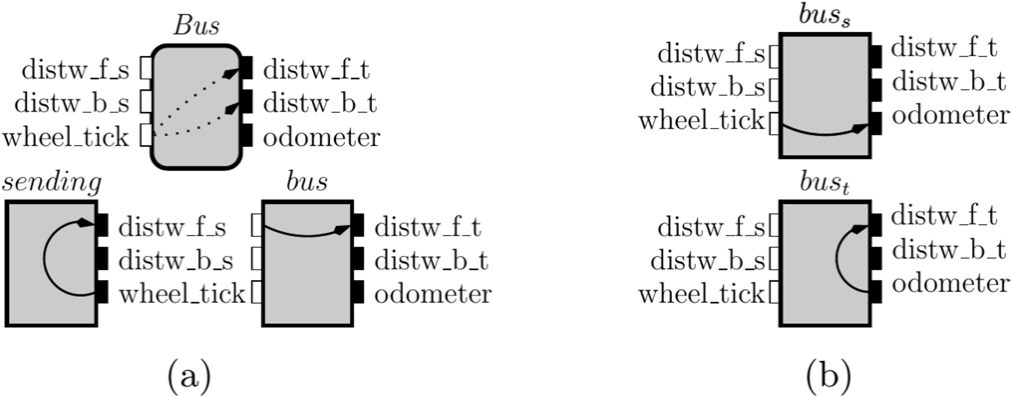
Interface *Bus* implementations – *bus*, $bus _s$ and $bus _t$ – and one of its permissible environments – *sending*

### Example 1

In Fig. 8, we have the first refinement of the interface *Bus* from our application example. The *Bus* interface specifies the requirement on the closed system that there are no information flows from *wheel_tick* to both *distw_f_t* and *distw_b_t*. The *Bus* interface also specifies this requirement as a guarantee on the open system. Then, the *bus* component (in the same figure) is an implementation of *Bus* because it has only a flow from *distw_f_s* to *distw_f_t*, which is not in the open-guarantee of the *Bus* interface. *Bus* does not have any requirement in its assumption, then the *sending* component is a permissible environment for *Bus*.

The composition of the *sending* and *bus* components shows that the *Bus* interface is not well-formed because there is a flow from *wheel_tick* to *distw_f_t*, which is a flow forbidden by the closed-guarantee of *Bus*. Hence, the assumption and open-guarantee in *Bus* are insufficient to enforce the *Bus* closed-guarantee.

### Definition 4

An information-flow interface $(X, Y, {\mathcal {A}}, {\mathcal {G}}, {\mathcal {P}})$ is *well-formed* iff ${\mathcal {A}}$, ${\mathcal {G}}$ and ${\mathcal {P}}$ are *no-flow relations* (i.e., irreflexive relations); and the closed-guarantee is consistent with the open-guarantee and assumption, i.e. $({\mathcal {A}}\bullet {\mathcal {G}}) \cap {\mathcal {P}}= \emptyset$.

The proposition below proves that our definition of well-formedness captures the intended relation between a well-formed interface closed-guarantee with its permissible environments and implementations.

### Proposition 1

For all well-formed interfaces $F= (X, Y, {\mathcal {A}}, {\mathcal {G}}, {\mathcal {P}})$, and for all components $f= (X, Y, {\mathcal {M}})$ and $f_{{\mathcal {E}}} = (Y, X, {\mathcal {E}})$: if $f$ implements $F$, $f\models _{{\mathcal {G}}} F$, and $f_{{\mathcal {E}}}$ is a permissible environment of $F$, $f_{{\mathcal {E}}} \models _{\!\!{\mathcal {A}}} F$, then their combined flows are consistent with the closed-guarantee of $F$, $({\mathcal {M}}\cup {\mathcal {E}})^* \cap {\mathcal {P}}= \emptyset$.

### Proof

Consider an arbitrary interface $F$, and components $f= (X, Y, {\mathcal {M}})$ and $f_{{\mathcal {E}}} = (Y, X, {\mathcal {E}})$, s.t.: (i) $F$ is a well-formed interface, (ii)$f\models _{{\mathcal {G}}} F$, and (iii) $f_{{\mathcal {E}}} \models _{\!\!{\mathcal {A}}} F$. Let $(z,z') \in ({\mathcal {M}}\cup {\mathcal {E}})^*$. By Definition 3 and assumptions (ii) and (iii), $(z,z') \in {\mathcal {A}}\bullet {\mathcal {G}}$, and by our initial assumption (i), $(z,z') \notin {\mathcal {P}}$. Hence $({\mathcal {M}}\cup {\mathcal {E}})^* \cap {\mathcal {P}}= \emptyset$. $\square$

Though the definition of complement-flow composition is intuitive, it is not obvious whether there is an economic way to compute it. Note that the definition requires evaluating the reflexive and transitive closure over all subsets of the composed relations complement that are flow relations. We will now discuss how to characterize the complement-flow composition syntactically, in particular, as a regular expression over flow relations.

Our first challenge is that the complement of an arbitrary relation does not necessarily define a flow relation because it may not be transitively closed. The biggest challenge, however, is that not all relations have a maximal flow relation that is a subset of its complement, i.e., no flow relation subsumes all flow relations that are subsets of a given relation complement. Within our theory, this means that not all interfaces have maximal implementations or maximal permissible environments.

### Example 2

In Fig. 8, we have two components, $bus_s$ and $bus_t$, that implement the interface *Bus* from the previous example. A maximal implementation of *Bus* must include the flows in both $bus_s$ and $bus_t$. As flows are transitively closed, the maximal implementation would include a flow from $wheel\_tick$ to $distw\_f\_t$, which violates the *Bus* open-guarantee and, therefore, does not define an implementation of *Bus*.

Without maximal implementations and maximal permissible environments, we cannot characterize the flow relation of the closed system defined by an interface $F$ by the transitive closure of all pairs of variables in the complement of both its assumption and open-guarantee, i.e., $(\overline{{\mathcal {A}}}\cup \overline{{\mathcal {G}}})^*$. Note that this approach would yield more flows than the flows of the closed system defined by $F$. Instead, the flow relation of the closed system defined by $F$ includes all pairs of variables $(z,z')$ such that there exists a path from *z* to $z'$ that alternates between flows in the complement of the assumption, $\overline{{\mathcal {A}}}$, and the complement of the open-guarantee, $\overline{{\mathcal {G}}}$. We illustrate this intuition below and formalize it in Lemma 2.

### Example 3

We present a step-by-step evaluation of the the complement-flow composition between the two open-guarantees as it will be formalized in the Lemma 2 after this example.

**Fig. 9:**
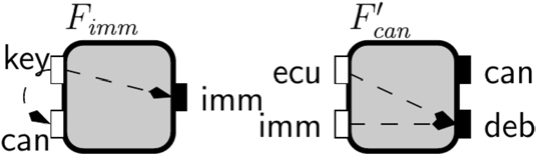
Interfaces to illustrate complement-flow composition in Example 3

Consider the interfaces $F_{imm }$ and $F'_{can }$ in Fig. 9 with open-guarantees:

$$\begin{aligned} \begin{aligned} {\mathcal {G}}_{F_{imm }}&= \{(\textsf {key}, \textsf {imm}), (\textsf {key},\textsf {can})\}\text { and }{\mathcal {G}}_{F'_{can }} = \{(\textsf {ecu}, \textsf {deb}), (\textsf {imm},\textsf {deb})\}. \end{aligned} \end{aligned}$$

As stated in Lemma 2, we can define the complement-flow composition between $F_{imm }$ and $F'_{can }$ interfaces’ open-guarantees by the following alternating composition between their open-guarantess complement:

$$\begin{aligned} {\mathcal {G}}_{F_{\textsf {imm}}} \bullet {\mathcal {G}}_{F'_{\textsf {can}}} = (\textrm{Id}_{Z} \cup \overline{{\mathcal {G}}}_{F_{\textsf {imm}}}) \circ (\overline{{\mathcal {G}}}_{F'_{\textsf {can}}} \circ \overline{{\mathcal {G}}}_{F_{\textsf {imm}}})^* \circ (\textrm{Id}_{Y} \cup \overline{{\mathcal {G}}}_{F'_{\textsf {can}}}), \end{aligned}$$

where $$\textrm{Id}_{Z} = \{(z,z)\ \mid \ z \in Z_{F_{\textsf {imm}}} \cup Z_{F'_{\textsf {can}}}\}$$ and $$\textrm{Id}_{Y} = \{(z,z)\ \mid \ z \in Y_{F_{\textsf {imm}}} \cup Y_{F'_{\textsf {can}}}\}$$.

We start by considering the set of flows that can be in some implementation of the immobilizer or the can bus, i.e., their open-guarantees’ complement:

$$\begin{aligned} \begin{aligned} \overline{{\mathcal {G}}}_{F_{\textsf {imm}}}&= \{(\textsf {can}, \textsf {imm}), (\textsf {imm},\textsf {imm})\} \text { and }\\ \overline{{\mathcal {G}}}_{F'_{\textsf {can}}}&= \{(\textsf {ecu}, \textsf {can}), (\textsf {imm}, \textsf {can}), (\textsf {can}, \textsf {deb}), (\textsf {deb}, \textsf {can}), (\textsf {can}, \textsf {can}), (\textsf {deb},\textsf {deb}) \}. \end{aligned} \end{aligned}$$

When we consider sequences of pairs with two steps defined between alternating implementations of the immobilizer and the can bus we get the sets:
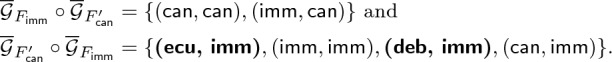
We highlight in bold pairs that were not present in the previous iteration. The new pair $(\textsf {ecu}, \textsf {imm})$ is defined by the sequence that starts with $(\textsf {ecu}, \textsf {can})$ from $\overline{{\mathcal {G}}}_{F'_{\textsf {can}}}$ followed by $(\textsf {can}, \textsf {imm})$ in $\overline{{\mathcal {G}}}_{F_{\textsf {imm}}}$. The two-step sequences added two new possible flows: from the ECU input port and the debug output port of the CAN bus to the $\textsf {imm}$ output port in the immobilizer, respectively. We consider next three step sequences:

$$\begin{aligned} \begin{aligned} (\overline{{\mathcal {G}}}_{F_{\textsf {imm}}} \circ \overline{{\mathcal {G}}}_{F'_{\textsf {can}}})\circ \overline{{\mathcal {G}}}_{F_{\textsf {imm}}}&= \{(\textsf {can}, \textsf {imm}), (\textsf {imm},\textsf {imm})\} = \overline{{\mathcal {G}}}_{F_{\textsf {imm}}},\\ \overline{{\mathcal {G}}}_{F_{\textsf {imm}}} \circ (\overline{{\mathcal {G}}}_{F'_{\textsf {can}}}\circ \overline{{\mathcal {G}}}_{F_{\textsf {imm}}})&= \{(\textsf {can}, \textsf {imm}), (\textsf {imm},\textsf {imm}) \}= \overline{{\mathcal {G}}}_{F_{\textsf {imm}}},\\ (\overline{{\mathcal {G}}}_{F'_{\textsf {can}}} \circ \overline{{\mathcal {G}}}_{F_{\textsf {imm}}}) \circ \overline{{\mathcal {G}}}_{F'_{\textsf {can}}}&= \{(\textsf {ecu}, \textsf {can}), (\textsf {imm},\textsf {can}), (\textsf {deb},\textsf {can}), (\textsf {can},\textsf {can})\} \subseteq \overline{{\mathcal {G}}}_{F'_{\textsf {can}}}\text { and }\\ \overline{{\mathcal {G}}}_{F'_{\textsf {can}}} \circ (\overline{{\mathcal {G}}}_{F_{\textsf {imm}}} \circ \overline{{\mathcal {G}}}_{F'_{\textsf {can}}})&= \{(\textsf {ecu}, \textsf {can}), (\textsf {imm}, \textsf {can}), (\textsf {can}, \textsf {can})(\textsf {deb}, \textsf {can}) \} \subseteq \overline{{\mathcal {G}}}_{F'_{\textsf {can}}}. \end{aligned} \end{aligned}$$

We can now stop our evaluation because the sets we obtained are subsets of previous iterations and considering longer sequences will not define new pairs. Then, complement-flow composition between $F_{imm }$ and $F'_{can }$ interfaces’ open-guarantees is:

$$\begin{aligned} {\mathcal {G}}_{F_{\textsf {imm}}} \bullet {\mathcal {G}}_{F'_{\textsf {can}}} = \overline{{\mathcal {G}}}_{F_{\textsf {imm}}} \cup \overline{{\mathcal {G}}}_{F'_{\textsf {can}}} \cup \{(\textsf {ecu}, \textsf {imm}), (\textsf {deb}, \textsf {imm})\}. \end{aligned}$$

Before we prove the lemma below, we remark that interface and component composition is only meaningful for entities with disjoint sets of outputs. Otherwise, it would not be possible to determine which entity involved in the composition is responsible for enforcing requirements on an output. For no-flow relations, output disjointness is equivalent to requiring their sink sets to be disjoint. In Lemma 2 below, we present a syntactic definition for complement-flow composition and prove that it characterizes precisely the complement-flow composition (c.f., Definition 3) when we consider syntactically well-formed interfaces, i.e., the interfaces that do not share output variables and consist only of no-flow relations. Besides avoiding iterating through all possible subsets of no-flow relations to compute complement-flow composition, the syntactic characterization in Lemma 2 provides an inductive proof method for properties related to the composition of information-flow interfaces.

### Lemma 2

Let ${\mathcal {N}}\subseteq U\times V$ and ${\mathcal {N}}' \subseteq U' \times V'$ be no-flow relations, with $V$ and $V'$ being disjoint sets, $V\cap V'=\emptyset$, and the set of all variables being $Z= U\cup U' \cup V\cup V'$. Then, ${\mathcal {N}}\bullet {\mathcal {N}}' = (\textrm{Id}_{Z} \cup {\overline{{\mathcal {N}}}}) \circ (\overline{{\mathcal {N}}'} \circ {\overline{{\mathcal {N}}}})^* \circ (\textrm{Id}_{V\cup V'} \cup \overline{{\mathcal {N}}'})$, where $\textrm{Id}_{Z}$ is the identity relation over all variables in ${\mathcal {N}}$ and ${\mathcal {N}}'$, $\textrm{Id}_{V\cup V'}$ is the identity relation over $V\cup V'$ and $R \circ R' = \{(z,z'')\mid (z,z') \in R \text { and }(z',z'') \in R'\}$ is the usual composition between relations.

### Proof

Consider arbitrary no-flow relations ${\mathcal {N}}\subseteq U\times V$ and ${\mathcal {N}}' \subseteq U' \times V'$ where $V$ and $V'$ are disjoint sets.

We start by proving that ${\mathcal {N}}\bullet {\mathcal {N}}' \subseteq (\textrm{Id}_{Z} \cup {\overline{{\mathcal {N}}}}) \circ (\overline{{\mathcal {N}}'} \circ {\overline{{\mathcal {N}}}})^* \circ (\textrm{Id}_{V\cup V'} \cup \overline{{\mathcal {N}}'})$. Let $(z,z') \in {\mathcal {N}}\bullet {\mathcal {N}}'$. Then, there exists two flow relations ${\mathcal {M}}\subseteq U\times V$ and ${\mathcal {M}}'\subseteq U' \times V'$ s.t. (i) ${\mathcal {M}}\subseteq {\overline{{\mathcal {N}}}}$, (ii) ${\mathcal {M}}' \subseteq \overline{{\mathcal {N}}'}$ and (iii) $(z,z') \in ({\mathcal {M}}\cup {\mathcal {M}}')^*$ or, equivalently, $(z,z') \in (\textrm{Id}_{Z} \cup {\mathcal {M}}^+) \circ ({\mathcal {M}}'^+ \circ {\mathcal {M}}^+)^* \circ (\textrm{Id}_{V\cup V'} \cup {\mathcal {M}}'^+)$. The identity relation in the rightmost side of the expression is defined over the domain $V\cup V'$ (i.e., $\textrm{Id}_{V\cup V'}$) because $z' \in V\cup V'$. By flow relations being transitively closed, it follows: $(z,z') \in (\textrm{Id}_{Z} \cup {\mathcal {M}}) \circ ({\mathcal {M}}' \circ {\mathcal {M}})^* \circ (\textrm{Id}_{V\cup V'} \cup {\mathcal {M}}')$. By the initial assumptions (i) and (ii), $(z,z') \in (\textrm{Id}_{Z} \cup {\overline{{\mathcal {N}}}}) \circ (\overline{{\mathcal {N}}'} \circ {\overline{{\mathcal {N}}}})^* \circ (\textrm{Id}_{V\cup V'} \cup \overline{{\mathcal {N}}'})$.

We prove the other direction: $(\textrm{Id}_{Z} \cup {\overline{{\mathcal {N}}}}) \circ (\overline{{\mathcal {N}}'} \circ {\overline{{\mathcal {N}}}})^* \circ (\textrm{Id}_{V\cup V'} \cup \overline{{\mathcal {N}}'})\subseteq {\mathcal {N}}\bullet {\mathcal {N}}'$. We start with the case $\overline{{\mathcal {N}}'} \circ ({\overline{{\mathcal {N}}}} \circ \overline{{\mathcal {N}}'})^* \subseteq {\mathcal {N}}\bullet {\mathcal {N}}'$. We remark that all sequences defined by $\overline{{\mathcal {N}}'} \circ ({\overline{{\mathcal {N}}}} \circ \overline{{\mathcal {N}}'})^*$ have elements of $\overline{{\mathcal {N}}'}$ in the odd positions and elements of ${\overline{{\mathcal {N}}}}$ in the even positions. We choose this to simplify the presentation of the proof. The other cases can be proved analogously. We prove this case by proving first the stronger property below for all sequences defined by $\overline{{\mathcal {N}}'} \circ ({\overline{{\mathcal {N}}}} \circ \overline{{\mathcal {N}}'})^*$:

($\star$) for all $n\in {\mathbb {N}}$ and all sequences $(z_1,z_2) \cdot (z_2,z_3) \cdot \ldots \cdot (z_{n}, z_{n+1})$ where $(z_{2i-1},z_{2i}) \in \overline{{\mathcal {N}}'}$ and $(z_{2i},z_{2i+1}) \in {\overline{{\mathcal {N}}}}$, with $1 \le i \le \lceil n/2 \rceil$, there is $1 \le m \le n$ s.t. $z_1 = z_{m}$ and $(z_{1},z_{n+1}) \in ({\mathcal {M}}\cup {\mathcal {M}}')^*$ for flow relations ${\mathcal {M}}' = \{(z_{j},z_{j+1})\,\mid \, m \le j \le n \text { and }j \text { is odd} \}^+ \cup \textrm{Id}_{B'}$ and ${\mathcal {M}}= \{(z_{j},z_{j+1})\,\mid \, m \le j \le n \text { and }j \text { is even}\}^+ \cup \textrm{Id}_{B}$, with ${\mathcal {M}}' \subseteq \overline{{\mathcal {N}}'}$ and ${\mathcal {M}}\subseteq {\overline{{\mathcal {N}}}}$.

The property above tell us that for any alternating sequence between elements in the complement of ${\mathcal {N}}$ and ${\mathcal {N}}'$ defining a path from $z_1$ to $z_{n+1}$, we can use this sequence to define two flow relations that are allowed by either ${\mathcal {N}}$ or ${\mathcal {N}}'$ such that $(z_1, z_{n+1})$ is in composition of the defined flow relations. From this property, it follows that for all pair of variables $(z,z') \in \overline{{\mathcal {N}}'} \circ ({\overline{{\mathcal {N}}}} \circ \overline{{\mathcal {N}}'})^*$, we can define two flow relations ${\mathcal {M}}$ and ${\mathcal {M}}'$ that witness $(z,z') \in {\mathcal {N}}\bullet {\mathcal {N}}'$. The main challenge in defining such witnessing flow relations for a pair $(z,z')$ is that it is not enough to define the flows relations ${\mathcal {M}}$ and ${\mathcal {M}}'$ as the sets of all elements in the complement of ${\mathcal {N}}$ and ${\mathcal {N}}'$, respectively, in a given alternating sequence from *z* to $z'$. Such flow relations may not define proper witnesses, i.e., it may be that ${\mathcal {M}}\not \subseteq {\overline{{\mathcal {N}}}}$ or ${\mathcal {M}}' \not \subseteq \overline{{\mathcal {N}}'}$.
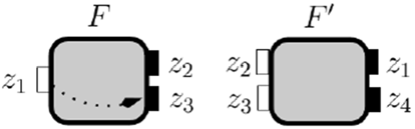


For example, consider the interfaces to our left with open-guarantees ${\mathcal {G}}_{F} =\{(z_1, z_3)\}$ and ${\mathcal {G}}_{F'} = \emptyset$. A possible alternating sequence derived from the interfaces’ guarantees complement is $(z_2, z_3) \cdot (z_3, z_1) \cdot (z_1, z_2) \cdot (z_2, z_4)$ defining a path from $z_2$ to $z_4$. Consider the flow relations ${\mathcal {M}}_{F} = \{(z_1, z_2), (z_2, z_3)\}^*$ and ${\mathcal {M}}_{F'} = \{(z_3, z_1), (z_2,z_4)\}^*$ defined by pairs in the sequence that are in $\overline{{\mathcal {G}}}_{F}$ and $\overline{{\mathcal {G}}}_{F'}$, respectively. Then, ${\mathcal {M}}_{F} \not \subseteq \overline{{\mathcal {G}}}_{F}$ because $(z_1,z_3) \in {\mathcal {M}}_{F}$. We prove below that when such a case occurs, there exists a cycle at the beginning of the sequence that can be removed. Once the cycle is removed, the new sequence defines an alternating path from the same source to the same sink (of the original sequence) that can be used directly to define our intended flow relations. In this example, we can remove the path prefix $(z_2, z_3) \cdot (z_3, z_1) \cdot (z_1, z_2)$, to get the path $(z_2,z_4)$ which satisfies our requirements.

We prove now ($\star$) by natural induction on the size of the sequences. We start with the *base case*, $n=1$, i.e., we consider a sequence of the form $(z_1, z_2)$ where $(z_{1},z_{2}) \in \overline{{\mathcal {N}}'}$. Then, for $k=1$, we have $({\mathcal {M}}' = \{(z_1,z_2)\} \cup \textrm{Id}_{U'}) \subseteq \overline{{\mathcal {N}}'}$ and $({\mathcal {M}}= \textrm{Id}_{U})\subseteq {\overline{{\mathcal {N}}}}$ (no-flow relations are irreflexive).

For the *induction step*, we assume by induction hypothesis (IH) that ($\star$) holds for an arbitrary $n\in {\mathbb {N}}$. Consider arbitrary sequence $\sigma = (z_1,z_2) \cdot \ldots \cdot (z_{n+1}, z_{n+2})$ of size $n+1$. By (IH), there exists $1 \le m_{n} \le n$ defining ${\mathcal {M}}_n$ and ${\mathcal {M}}_n'$ over the $\sigma$’s sub-sequence $(z_1,z_2) \cdot (z_2,z_3) \cdot \ldots \cdot (z_{n}, z_{n+1})$ of size *n*, as specified in $(\star )$, s.t. ${\mathcal {M}}_n' \subseteq \overline{{\mathcal {N}}'}$ and ${\mathcal {M}}_n \subseteq {\overline{{\mathcal {N}}}}$. We proceed by cases on the parity of $n+1$.

Let $n+1$ *be an odd number*, then $(z_{n+1}, z_{n+2}) \in \overline{{\mathcal {N}}'}$. Consider the case that the last pair in the sequence, $(z_{n+1}, z_{n+2})$, together with ${\mathcal {M}}_n'$ defines a flow relation that is a subset of $\overline{{\mathcal {N}}'}$, i.e., $({\mathcal {M}}_n' \cup \{(z_{n+1}, z_{n+2})\})^* \subseteq \overline{{\mathcal {N}}'}$. Then, by (IH) there exists $m = m_n$ that satisfies $(\star )$ for the sequence of size $n+1$. Otherwise, there exists a sequence using $(z_{n+1}, z_{n+2})$ and elements of ${\mathcal {M}}_n'$ defining a path between a pair of variables in ${\mathcal {N}}'$. Formally, there exists a sequence for $(z_1', z_{k+1}') \in {\mathcal {N}}'$:

$$\begin{aligned} (z_1',z'_2) \cdot \ldots \cdot (z_{n+1}, z_{n+2}) \cdot \ldots \cdot (z'_{k},z'_{k+1}) \end{aligned}$$

where $k \in {\mathbb {N}}$ and $\{(z_1',z'_2) \cdots (z'_{k},z'_{k+1})\} \subseteq \{(z_{j},z_{j+1})\,\mid \, m \le j \le n \text { and }j \text { is odd} \}$. Note that both the sequence before and the sequence after $(z_{n+1}, z_{n+2})$ may be empty, i.e., the path may start or end with $(z_{n+1}, z_{n+2})$. As ${\mathcal {M}}_n'$ is transitively closed, the sequence above can be simplified to:

$$\begin{aligned} (\dagger ) \ (z'_{1},z_{n+1}) \cdot (z_{n+1}, z_{n+2}) \cdot (z_{n+2},z'_{2}) \end{aligned}$$

where $\{(z'_{1},z_{n+1}),(z_{n+2},z'_{2})\} \in {\mathcal {M}}_n'$. Recall that, ${\overline{{\mathcal {N}}}} \subseteq U\times V$ and $\overline{{\mathcal {N}}'} \subseteq U' \times V'$. By $V$ and $V'$ being disjoint sets and $(z_n, z_{n+1}) \in {\overline{{\mathcal {N}}}}$, it follows that $z_{n+1} \in V$ and $z_{n+1} \notin V'$. Then, for all variables *z*, $(z, z_{n+1}) \notin {\mathcal {M}}_n'$. Hence the sequence $(\dagger )$ must start with the pair $(z_{n+1}, z_{n+2})$. As $(z_{n+2}, z_{2}') \in {\mathcal {M}}_n'$ then there exists a pair in the original sequence that starts with $z_{n+2}$ and another pair that ends with $z_{2}'$. In particular, for $m_n \le h \le h' \le (n+1)$, we have the following sub-sequence of $\sigma$:

$$\begin{aligned} \underset{=}{z_{n+2}}{} & {} \\ (z_{m_{n}}, z_{m_{n}+1}) \cdot \ldots \cdot (z_{h-1} , z_{h}) \cdot (\ \ z_{h} \ \ {}&, z_{h+1}) \cdot \ldots \cdot \underset{=}{z'_2 \ } (z_{h'}, &z_{h'+1}&) \cdot \ldots \cdot (z_{n+1}, z_{n+2}).&\\ \end{aligned}$$

By $(z_{n+1}, z_{n+2}) \in \overline{{\mathcal {N}}'}$ and $V$ disjoint from $V'$, then $z_{n+2} \in V'$ and $z_{n+2} \notin V$. Then, as $z_h = z_{n+2}$, there is no pair $(z_{h-1}, z_{h}) \in {\overline{{\mathcal {N}}}}$ and the sequence above must start with $(z_{h}, z_{h+1})$. So, $m_n = h$ and $z_{m_{n}} = z_{h} = z_{n+2}$ and the previous sequence simplifies as follows:

$$\mathop {\left( {z_{{m_{n} }} \;\;,z_{{m_{n} + 1}} } \right)}\limits^{{\mathop {z_{{n + 2}} }\limits_{ = } }} \cdot \ldots \cdot \mathop {\left( {z_{{h^{\prime}}} ,z_{{h^{\prime} + 1}} } \right)}\limits^{{\mathop {z_{2}^{\prime } }\limits_{ = } }} \cdot \ldots \cdot \left( {z_{{n + 1}} ,z_{{n + 2}} } \right).$$

By (IH), $z_1 = z_{m_{n}}$. Let $m = n+1$. As $n+1$ is an odd number, it defines the flow relations ${\mathcal {M}}_{n+1}' = \{(z_{n+1}, z_{n+2})\} \cup \textrm{Id}_{V'}$ and ${\mathcal {M}}_{n+1} = \textrm{Id}_{V}$. Then, $(z_1,z_{n+2}) \in {\mathcal {M}}_{n+1}'$ because $z_{1} = z_{m_{n}} = z_{n+2}$ and $(z_{n+2}, z_{n+2}) \in \textrm{Id}_{V'}$. By both ${\mathcal {N}}$ and ${\mathcal {N}}'$ being no-flow relations and $(z_{n+1}, z_{n+2}) \in \overline{{\mathcal {N}}'}$, then ${\mathcal {M}}_{n+1}' \subseteq \overline{{\mathcal {N}}'}$ and ${\mathcal {M}}_{n+1} \subseteq {\overline{{\mathcal {N}}}}$.

If $n+1$ *is an even number*, then $(z_{n+1}, z_{n+2}) \in {\overline{{\mathcal {N}}}}$ and the argument is analogous.

We can prove analogously that ${\overline{{\mathcal {N}}}}\circ (\overline{{\mathcal {N}}'} \circ {\overline{{\mathcal {N}}}})^* \subseteq {\mathcal {N}}\bullet {\mathcal {N}}'$. Finally, note that $\textrm{Id}_{Z} \circ \textrm{Id}_{V\cup V'} \subseteq {\mathcal {N}}\bullet {\mathcal {N}}'$ follows directly from ${\mathcal {N}}$ and ${\mathcal {N}}'$ being no-flow relations and their domain. $\square$

### Composition and incremental design

This section presents *component and interface composition*. We introduce, additionally, a *compatibility* predicate between interfaces: two interfaces are compatible when their composition defines a well-formed interface. We prove that our notions of composition and compatibility support *incremental design* of systems.

From now on, to simplify presentation, elements of an interface (or component) tuple are annotated with the interface name, i.e., $F= (X_{F}, Y_{F}, {\mathcal {A}}_{F}, {\mathcal {G}}_{F}, {\mathcal {P}}_{F})$. The different types of variables between interfaces $F$ and $F'$ are defined as $Y_{F, F'} = Y_{F} \cup Y_{F'}$, $X_{F, F'} = (X_{F} \cup X_{F'}) {\setminus } Y_{F, F'}$, and $Z_{F, F'} = Y_{F, F'} \cup X_{F, F'}$. The same definition applies to components $f$ and $f'$. Variables between interfaces (components) define the set of variables in the composition of interfaces (components). We will often denote $X_{F, F'}$ as $X_{F\otimes F'}$, $Y_{F, F'}$ as $Y_{F\otimes F'}$, and $Z_{F, F'}$ as $Z_{F\otimes F'}$. The *composition of components* $f$ *and* $f'$ defines the component $f\otimes f' = (X_{f, f'}, Y_{f, f'}, ({\mathcal {M}}_{f} \cup {\mathcal {M}}_{f'})^*).$ We present interface composition by defining the open- and closed-guarantee, and the assumption of the composite separately.

We compose interfaces through their *shared variables*, i.e., all variables that are input for one of the interfaces while being output for the other. Interface composition must allow all implementations of the interfaces being composed. Then, the open-guarantee of such a composition must allow all flows already allowed by one of the interfaces being composed. Additionally, it must allow all flows in the composition of any of the interfaces’ implementations. To evaluate all flows in the composition of two interfaces, we compose their open-guarantees (as in Definition 3) and refer to them as *composite flows*.

#### Definition 5

Let $F$ and $F'$ be information-flow interfaces with open-guarantee ${\mathcal {G}}_{F}$ and ${\mathcal {G}}_{F'}$, respectively. The *composite* open-guarantee *of* $F$ *and* $F'$ is ${\mathcal {G}}_{F, F'} = (Z_{F, F'}\times Y_{F, F'}) {\setminus } ({\mathcal {G}}_{F}\bullet {\mathcal {G}}_{F'})$, also denoted ${\mathcal {G}}_{F\otimes F'}$.

We prove below that our definition of composite open-guarantee preserves all flows in the implementations of the interfaces. The proposition below follows directly from definition of complement-flow composition and complement of an open-guarantee.

#### Proposition 3

For all interfaces $F$ and $F'$ with open-guarantee ${\mathcal {G}}$ and ${\mathcal {G}}'$, respectively, and all components $f= (X, Y, {\mathcal {M}})$ and $f' = (X', Y', {\mathcal {M}}')$ that implement them, $f\models _{{\mathcal {G}}} F$ and $f' \models _{{\mathcal {G}}'} F'$, the composition of the components satisfies the restriction imposed by the open-guarantee defined by both interfaces, i.e., $({\mathcal {M}}\cup {\mathcal {M}}')^* \subseteq \overline{{\mathcal {G}}}_{F\otimes F'}$.

Recall that open-guarantees only specify constraints on their respective interface’s implementations. Consequently, as expected, the other direction of the proposition above does not hold. In the implementations of the composite interface, there may exist flows derived from the interaction between the interfaces being composed that were not allowed in the implementations of each interface individually. We illustrate this in the example below.

#### Example 4

We illustrate how an interface composite can include a flow that was not allowed in the implementations of the interfaces being composed. Consider the interfaces below, where the only no-flow requirement is that *x* does not flow to *y* specified in the open-guarantee of $F$:

$$\begin{aligned} \begin{aligned} F=(\{x,s\},\{y\}, \{\}, \{(x,y)\}, \{\}) \text { and }F'=(\{x\},\{s\}, \{\}, \{\}, \{\}) \end{aligned} \end{aligned}$$

We start by observing that the composite interface allows flows from *x* to *y* because there exist implementations of $F$ and $F'$ that, when composed, define the mentioned flow. One example of such implementation are the components $f_1$ and $f_1'$ defined below, which are implementations of $F$ and $F'$, respectively:

$$\begin{aligned} \begin{aligned} f_1 = (\{x,s\},\{y\},\{(y,y),(s,y)\}) \text { and }f_1' = (\{x\},\{s\},\{(s,s),(x,s)\}. \end{aligned} \end{aligned}$$

Note that we can have a flow from *x* to *s* through $f_1'$ followed by a flow from *s* to *y* through $f_1$. Then, $(x,y) \in \overline{{\mathcal {G}}}_{F\otimes F'}$. Now, we look at two different components:

$$\begin{aligned} \begin{aligned} f_2 = (\{x,s\},\{y\},\{(y,y),(x,y)\}) \text { and }f_2' = (\{x\},\{s\},\{(s,s)\}). \end{aligned} \end{aligned}$$

Then, while the component’s composite implements the interfaces’ composite (i.e., $(\{(y,y),(x,y)\} \cup \{(s,s)\})^* \subseteq \overline{{\mathcal {G}}}_{F\otimes F'}$), $f_2$ does not implement $F$.

We remark that the complement of the composite open-guarantee of interfaces $F$ and $F'$ and their open-guarantee no-flow composition define the same sets. Formally, $\overline{{\mathcal {G}}}_{F\otimes F'} = {\mathcal {G}}_{F} \bullet {\mathcal {G}}_{F'}$ because $\overline{{\mathcal {G}}}_{F\otimes F'} = (Z\times Y) {\setminus } ((Z\times Y) {\setminus } {\mathcal {G}}_{F} \bullet {\mathcal {G}}_{F'})$. It follows directly from the definition of no-flow composition that it defines a monotonic and associative operation. Then, the complement of a composite open-guarantee is also monotonic and associative, i.e., $\overline{{\mathcal {G}}}_{F} \subseteq \overline{{\mathcal {G}}}_{F\otimes F'}$ and $\overline{{\mathcal {G}}}_{F\otimes F'} = \overline{{\mathcal {G}}}_{F'\otimes F}$.

The assumption of an interface composition is the weakest condition in the environment allowing the interfaces being composed to work together while supporting incremental design. Incremental design of systems requires that interfaces’ compatibility for composition is independent of the order they are composed. Not all assumptions of the interfaces being composed will stay as assumptions of the composite interface. Note that shared variables between two interfaces are input variables for one of the interfaces but will be in the output variables of their composition. Formally, the set of *shared variables* of interfaces $F$ and $F'$, are $\textrm{Shared}_{F, F'} = (X_{F} \cup X_{F'}) \cap Y_{F, F'}.$ If the environment can influence the information flow to a shared variable, we may need to add assumptions to prevent that flow. We define below *derived assumptions*, which are new requirements on the environment’s information-flow derived from no-flow in the assumption pointing to shared variables.

**Fig. 10:**
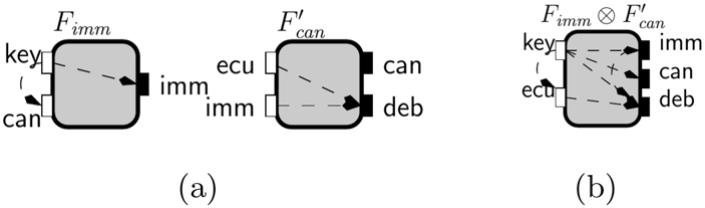
The result of composing interfaces in (a), depicted in (b), includes a derived assumption from $\textsf {key}$ to $\textsf {ecu}$

#### Example 5

In Fig. 10a, we depict an interface specifying information-flow policies for a car immobilizer, $F_{imm }$, along with an interface for a Controller Area Network (CAN bus), $F'_{can }$. Interface $F_{imm }$ has only one assumption that $\textsf {key}$ does not flow to $\textsf {can}$. In this design, the immobilizer uses the CAN to communicate with the car’s electronic control unit (ECU). Our goal is to compose both interfaces. These interfaces share the variable $\textsf {can}$: a shared variable between the interfaces and an output variable of their composition. The interface $F'_{can }$ cannot guarantee that the only assumption in $F_{imm }$ is satisfied after composition because it does not have a port $\textsf {key}$. As we are working with open systems and assume that the environment is helpful, we can add further assumptions to ensure the correctness of this composition. For example, we can add assumptions that prevent $\textsf {key}$ from flowing to an input port in $F'_{can }$ that can flow to $\textsf {can}$. Such flows could be part of a flow from $\textsf {key}$ to $\textsf {can}$, which would violate the assumption we want to enforce. In this case, we note that in $F'_{can }$ information in $\textsf {ecu}$ can flow to $\textsf {can}$. So, the composite interface must include the assumption that $\textsf {key}$ does not flow to $\textsf {ecu}$, which is a *derived assumption*. The derived assumption is depicted in Fig. 10b.

#### Definition 6

Let $F$ and $F'$ be information-flow interfaces with assumption ${\mathcal {A}}_{F}$ and ${\mathcal {A}}_{F'}$, respectively. The *assumption derived from* $F$ *and* $F'$ is:

$$\begin{aligned} \hat{{\mathcal {A}}}_{F, F'} = \{(z,z') \mid (z,s) \in {\mathcal {A}}_{F} \cup {\mathcal {A}}_{F'} \text { and }(z',s) \in \overline{{\mathcal {G}}}_{F\otimes F'}\}. \end{aligned}$$

Their *composite assumption* is ${\mathcal {A}}_{F\otimes F'} = ({\mathcal {A}}_{F} \cup {\mathcal {A}}_{F'} \cup \hat{{\mathcal {A}}}_{F, F'}) \cap (Z_{F, F'} \times X_{F, F'}).$

We remark that by definition of interface’s assumption and composite guarantee, the variable *s* in the definition of the derived assumption ${\mathcal {A}}_{F\otimes F'}$ must be an input variable of one of the interfaces (as $(z,s) \in {\mathcal {A}}_{F} \cup {\mathcal {A}}_{F'}$) while also being an output of one of them (as $(z',s) \in \overline{{\mathcal {G}}}_{F\otimes F'}$). In other words, *s* is a shared variable of $F$ and $F'$.

#### Example 6

From the example before, information from the ports $\textsf {ecu}$, $\textsf {imm}$ and $\textsf {deb}$ can all flow to $\textsf {can}$. So, they are flows in the composite interface and, by Definition 5, $\{(\textsf {ecu},\textsf {can}),(\textsf {imm},\textsf {can}), (\textsf {deb}, \textsf {can})\} \subseteq \overline{{\mathcal {G}}}_{F_{imm }, F'_{can }}$. Then, $\hat{{\mathcal {A}}}_{F_{imm }, F'_{can }} =\{(\textsf {key},\textsf {can}), (\textsf {key},\textsf {ecu}), (\textsf {key},\textsf {imm}), (\textsf {key},\textsf {deb})\}$. From those assumptions only $(\textsf {key},\textsf {ecu})$ points to a variable in $X_{F, F'}$, so ${\mathcal {A}}_{F_{imm }\otimes F'_{can }} = \{(\textsf {key},\textsf {ecu})\}$.

The closed-guarantee of the composition contains all pairs of variables in the closed-guarantees of each interface being composed. We observe that, as more is known about the system after composition, a composite interface may strengthen the closed-guarantees of each interface. For this reason, composite closed-guarantees include, additionally, all derived closed-guarantees from the assumption and open-guarantees of the composite. *Derived closed-guarantees* are all pair of variables (*z*, *y*) in a given open-guarantee such that no composition between any of its implementations with a permissible environment of a given assumption has a flow from *z* to *y*.

#### Definition 7

The *derived closed-guarantee* from an assumption ${\mathcal {A}}$ and open-guarantee ${\mathcal {G}}$ is $\hat{{\mathcal {P}}}_{{\mathcal {A}},{\mathcal {G}}} = {\mathcal {G}}{\setminus } ({\mathcal {A}}\bullet {\mathcal {G}})$. Let $F$ and $F'$ be information-flow interfaces with closed-guarantees ${\mathcal {P}}_{F}$ and ${\mathcal {P}}_{F'}$, respectively. Their composite closed-guarantee is the union of their closed-guarantees with the derived closed guarantee from the composite assumption and open-guarantee, i.e., ${\mathcal {P}}_{F\otimes F'} = {\mathcal {P}}_{F} \cup {\mathcal {P}}_{F'} \cup \hat{{\mathcal {P}}}_{{\mathcal {A}}_{F\otimes F'}, {\mathcal {G}}_{F\otimes F'}}$.

Using the definitions of composite assumption, composite open-guarantee, and derived closed-guaranteed introduced above, we define below the composition between any two interfaces. However, not all compositions are meaningful. So, in addition to the definition of composition, we specify a syntactic and a semantic criterion for composition. We start by observing that output variables are the responsibility of the interface’s implementations. For this reason, we introduce the syntactic requirement that interfaces should not have overlapping output variables, as it would not be possible to establish which of the interfaces being composed is responsible for enforcing guarantees on the overlapping variables. We say that two interfaces are *composable* when both interfaces’ output variables are disjoint. From the semantic point of view, we say that interfaces $F$ and $F'$ are *compatible* if whenever one of them provides inputs (e.g., $F$) to the other interface (e.g., $F'$), then the open-guarantee of the first (${\mathcal {G}}_{F}$) includes no-flow requirements that support the assumption of the second interface (${\mathcal {A}}_{F'}$).

#### Definition 8

The *composition* of information-flow interfaces $F$ and $F'$ is the information-flow interface $F\otimes F= (X_{F, F'}, Y_{F, F'}, {\mathcal {A}}_{F\otimes F'}, {\mathcal {G}}_{F\otimes F'}, {\mathcal {P}}_{F\otimes F'}),$ where the composite assumption ${\mathcal {A}}_{F\otimes F'}$ is as in Definition 6, the composite open-guarantee ${\mathcal {G}}_{F\otimes F'}$ is as in Definition 5, and the composite closed-guarantee ${\mathcal {P}}_{F\otimes F'}$ is as in Definition 7. Interfaces $F$ and $F'$ are *composable* iff $Y_{F} \cap Y_{F'} = \emptyset$; and they are *compatible*, denoted $F\sim F'$, iff they are composable and $(({\mathcal {A}}_{F} \cup {\mathcal {A}}_{F'}) \cap (Z_{F, F'} \times Y_{F, F'}))\subseteq {\mathcal {G}}_{F\otimes F'}.$

For well-formed and compatible interfaces, we can simplify the definition of composite closed-guarantees to consider only the derived closed-guarantees between composite assumption and open-guarantee, proved below.

#### Lemma 4

For all well-formed and compatible information-flow interfaces $F$ and $F'$ with closed-guarantees ${\mathcal {P}}_{F}$ and ${\mathcal {P}}_{F'}$, respectively. The derived closed-guarantees of their composite assumption and open-guarantee subsumes each interface closed-guarantee; formally, ${\mathcal {P}}_{F} \cup {\mathcal {P}}_{F'} \subseteq \hat{{\mathcal {P}}}_{{\mathcal {A}}_{F\otimes F'}, {\mathcal {G}}_{F\otimes F'}}$

#### Proof

Let $F$ and $F'$ be well-formed and compatible information-flow interfaces. To prove our intended statement, we start by proving that any path alternating between elements in $\overline{{\mathcal {A}}}_{F\otimes F'}$ and $\overline{{\mathcal {G}}}_{F\otimes F'}$ from any variable of $F$ (or $F'$) to an output variable of the same interface can be translated to a path using only the complements of assumptions and open-guarantees of $F$ (or $F'$). Formally, for compatible interfaces $F$ and $F'$, and all $n \in {\mathbb {N}}$:

$(\star )$ if $(z,z') \in Z_{F} \times Y_{F}$ and $(z,z') \in (\textrm{Id}_{Z_{F\otimes F'}} \cup \overline{{\mathcal {A}}}_{F\otimes F'}) \circ (\overline{{\mathcal {G}}}_{F\otimes F'} \circ \overline{{\mathcal {A}}}_{F\otimes F'})^n \circ \overline{{\mathcal {G}}}_{F\otimes F'}$, then exists $m\in {\mathbb {N}}$ s.t. $(z,z') \in (\textrm{Id}_{Z_{F}} \cup \overline{{\mathcal {A}}}_{F}) \circ (\overline{{\mathcal {G}}}_{F} \circ \overline{{\mathcal {A}}}_{F})^m \circ \overline{{\mathcal {G}}}_{F}.$

This property relies on the interfaces’ compatibility requirement that assumptions to their shared variables are covered by flows allowed by their composite open-guarantee. We prove $(\star )$ by natural induction on *n*. Consider arbitrary compatible interfaces $F$ and $F'$ and let $(z,z') \in Z_{F} \times Y_{F}$.

For the *base case,* $n=0$, $(z,z') \in \overline{{\mathcal {G}}}_{F\otimes F'} \cup ( \overline{{\mathcal {A}}}_{F\otimes F'} \circ \overline{{\mathcal {G}}}_{F\otimes F'})$. We proceed by cases on the $(z,z')$ domain.

If $(z,z') \in \overline{{\mathcal {G}}}_{F\otimes F'}$ and, by $z'\in Y_{F}$ and Lemma 2, then the last flow of any path from *z* to $z'$ must be in $\overline{{\mathcal {G}}}_{F}$, i.e., $(z,z') \in (\textrm{Id}_{F,F'} \cup \overline{{\mathcal {G}}}_{F'}) \circ (\overline{{\mathcal {G}}}_{F}\circ \overline{{\mathcal {G}}}_{F'})^* \circ \overline{{\mathcal {G}}}_{F}$. Let $(z,s) \in (\textrm{Id}_{F,F'} \cup \overline{{\mathcal {G}}}_{F'}) \circ (\overline{{\mathcal {G}}}_{F}\circ \overline{{\mathcal {G}}}_{F'})^*$ and $(s,z') \in \overline{{\mathcal {G}}}_{F}$. If $(z,s) = (s,s)$, then $(z,z') \in \overline{{\mathcal {G}}}_{F}$. Otherwise, by the interfaces being compatible, their set of output variables are disjoint, and $s \in Y_{F'}$ and $s\in X_{F}$. Again by interfaces compatibility, $(z,s) \in Z_{F,F'} \times Y_{F,F'}$ and $(z,s) \in \overline{{\mathcal {G}}}_{F\otimes F'}$, then $(z,s) \notin (({\mathcal {A}}_{F} \cup {\mathcal {A}}_{F'}) \cap Z_{F\otimes F'} \times Y_{F\otimes F'})$. So, in particular, $(z,s) \notin {\mathcal {A}}_{F}$ and, by $s\in X_{F}$, $(z,s) \in \overline{{\mathcal {A}}}_{F}$ Hence, $(z,z') \in \overline{{\mathcal {A}}}_{F} \circ \overline{{\mathcal {G}}}_{F}$.

If $(z,z') \in \overline{{\mathcal {A}}}_{F\otimes F'} \circ \overline{{\mathcal {G}}}_{F\otimes F'}$, then, by $z'\in Y_{F}$ and Lemma 2, $(z,z') \in \overline{{\mathcal {A}}}_{F\otimes F'} \circ (\textrm{Id}_{F,F'} \cup \overline{{\mathcal {G}}}_{F'}) \circ (\overline{{\mathcal {G}}}_{F}\circ \overline{{\mathcal {G}}}_{F'})^* \circ \overline{{\mathcal {G}}}_{F}$. Consider arbitrary:

$$\begin{aligned} (z,s) \in \overline{{\mathcal {A}}}_{F\otimes F'},\ (s,s') \in (\textrm{Id}_{F,F'} \cup \overline{{\mathcal {G}}}_{F'}) \circ (\overline{{\mathcal {G}}}_{F}\circ \overline{{\mathcal {G}}}_{F'})^* \text { and }(s',y) \in \overline{{\mathcal {G}}}_{F}. \end{aligned}$$

If $(s,s')=(s',s')$, then $(z,s') \in \overline{{\mathcal {A}}}_{F\otimes F'}$ and $(s',y) \in \overline{{\mathcal {G}}}_{F}$. As $(z,s') \in \overline{{\mathcal {A}}}_{F\otimes F'}$, then $s'$ is an input variable of both interfaces ($s'\in X_{F\otimes F'}$) and, by $(s',y) \in \overline{{\mathcal {G}}}_{F}$ and definition of interface, $s'$ must be an input variable of $F$ ($s'\in X_{F}$). Then, by $(z,s') \in \overline{{\mathcal {A}}}_{F\otimes F'}$ and definition of composite assumptions, $(z,s') \notin {\mathcal {A}}_{F}$. So, by $s'\in X_{F}$, $(z,s') \in \overline{{\mathcal {A}}}_{F}$. If $(s,s')\ne (s',s')$, by interfaces compatibility, $s' \in Y_{F'}$ and $s' \in Z_{F}$, then $s' \in X_{F}$. Assume towards a contradiction that $(z,s') \in {\mathcal {A}}_{F}$. Then, by definition of derived assumptions and $(s,s') \in \overline{{\mathcal {G}}}_{F\otimes F'}$, $(z,s) \in \hat{{\mathcal {A}}}_{F,F'}$. As $(z,s) \in \overline{{\mathcal {A}}}_{F\otimes F'}$, then $s \in X_{F\otimes F'}$ and $(z,s) \in {\mathcal {A}}_{F\otimes F'}$. This contradicts $(z,s) \in \overline{{\mathcal {A}}}_{F\otimes F'}$. Hence $(z,s') \notin {\mathcal {A}}_{F}$ and so $(z,z') \in \overline{{\mathcal {A}}}_{F} \circ \overline{{\mathcal {G}}}_{F}$.

For the *induction step*, we assume as induction hypothesis the statement $(\star )$ holds for *n* and $(z,z') \in (\textrm{Id}_{Z_{F\otimes F'}} \cup \overline{{\mathcal {A}}}_{F\otimes F'}) \circ (\overline{{\mathcal {G}}}_{F\otimes F'} \circ \overline{{\mathcal {A}}}_{F\otimes F'})^{n+1} \circ \overline{{\mathcal {G}}}_{F\otimes F'}$. Then, by application of the induction hypothesis,

$$\begin{aligned} (z,z') \in (\textrm{Id}_{Z_{F}} \cup \overline{{\mathcal {A}}}_{F}) \circ (\overline{{\mathcal {G}}}_{F} \circ \overline{{\mathcal {A}}}_{F})^m \circ \overline{{\mathcal {G}}}_{F} \circ \overline{{\mathcal {A}}}_{F\otimes F'} \circ \overline{{\mathcal {G}}}_{F\otimes F'} \end{aligned}$$

for some $m \in {\mathbb {N}}$. The rest of the proof is analogous to the base case.

We prove now that for all pairs of variables that are not in the derived closed-guarantee, $(z,z') \notin \hat{{\mathcal {P}}}_{{\mathcal {A}}_{F\otimes F'}, {\mathcal {G}}_{F\otimes F'}}$, they are also not in the individual closed-guarantees, i.e., $(z,z') \in {\mathcal {P}}_{F} \cup {\mathcal {P}}_{F'}$. Consider arbitrary pair $(z,z') \notin \hat{{\mathcal {P}}}_{{\mathcal {A}}_{F\otimes F'}, {\mathcal {G}}_{F\otimes F'}}$. Note that the domain of ${\mathcal {P}}_{F}$ and ${\mathcal {P}}_{F'}$ is $Z_{F} \times Y_{F}$ and $Z_{F'} \times Y_{F'}$, respectively. If $(z,z')$ is not in the union of these domains, then $(z,z') \notin {\mathcal {P}}_{F} \cup {\mathcal {P}}_{F'}$. Let $(z,z') \in (Z_{F} \times Y_{F}) \cup (Z_{F'} \times Y_{F'})$. Note that, by Lemma 2, $(z,z') \in (\textrm{Id}_{Z_{F,F}} \cup \overline{{\mathcal {A}}}_{F\otimes F'}) \circ (\overline{{\mathcal {G}}}_{F\otimes F'} \circ \overline{{\mathcal {A}}}_{F\otimes F'})^* \circ \overline{{\mathcal {G}}}_{F\otimes F'}$. Now, if we consider the case that $z' \in Y_{F}$, then, by the interfaces being compatible, $z'\notin Y_{F'}$. Thus, $(z,z') \in Z_{F} \times Y_{F}$, $(z,z') \notin Z_{F'} \times Y_{F'}$ and, by definition of interface, $(z,z') \notin {\mathcal {P}}_{F'}$. By $(z,z') \in Z_{F} \times Y_{F}$, $F\sim F$ and $(\star )$, $(z,z') \in (\textrm{Id}_{Z_{F,F'}} \cup \overline{{\mathcal {A}}}_{F}) \circ (\overline{{\mathcal {G}}}_{F} \circ \overline{{\mathcal {A}}}_{F})^* \circ \overline{{\mathcal {G}}}_{F}$, i.e., $(z,z') \in {\mathcal {A}}_{F} \bullet {\mathcal {G}}_{F}$. Hence, by $F$ being well-formed, $(z,z') \notin {\mathcal {P}}_{F}$, as well. The case for $z' \in Y_{F'}$ is analogous. $\square$

We now prove important properties about information-flow interface’s composition. Clearly, both the composition operator and the compatibility relation are commutative. We prove below that composition between compatible interfaces preserves well-formedness.

#### Theorem 5

Let $F$ and $F'$ be well-formed information-flow interfaces. If they are compatible, $F\sim F'$, then their composition, $F\otimes F'$, defines a well-formed interface.

#### Proof

Consider arbitrary well-formed interfaces $F$ and $F'$, and assume they are compatible, $F\sim F'$. By definition of composition, both the composite open- and closed-guarantee, ${\mathcal {G}}_{F\otimes F'}$ and ${\mathcal {P}}_{F\otimes F'}$, define no-flow relations (i.e. irreflexive). Now, assume towards a contradiction that composite assumptions are not irreflexive, i.e., there exists an input variable $x \in X_{F, F'}$ s.t. $(x,x) \in {\mathcal {A}}_{F\otimes F'}$. By $F$ and $F'$ being well-formed interfaces, then $(x,x) \notin {\mathcal {A}}_{F} \cup {\mathcal {A}}_{F'}$. So, by definition of composite assumptions, it must be the case that $(x,x) \in {\hat{\mathcal {A}}}_{F,F'}$. By definition of derived assumptions, there exists a variable *s* s.t. $(x,s) \in {\mathcal {A}}_{F} \cup {\mathcal {A}}_{F'}$ and $(x,s) \in \overline{{\mathcal {G}}}_{F,F'}$. This contradicts our initial assumption that $F\sim F'$. Hence the composite assumption also defines an irreflexive relation.

To prove that $({\mathcal {A}}_{F\otimes F} \bullet {\mathcal {G}}_{F\otimes F'}) \cap \overline{{\mathcal {P}}}_{F\otimes F'} = \emptyset$, we start by observing that, by both $F$ and $F'$ being well-formed and Lemma 4, $\overline{{\mathcal {P}}}_{F\otimes F'} = \overline{\hat{{\mathcal {P}}}_{F\otimes F'}}$. Consider arbitrary $(z,z') \in {\mathcal {A}}_{F\otimes F} \bullet {\mathcal {G}}_{F\otimes F'}$. Then, by definition of derived closed-guarantee, $(z,z') \notin \hat{{\mathcal {P}}}_{{\mathcal {A}}_{F\otimes F'}, {\mathcal {G}}_{F\otimes F'}}$. $\square$

Our next step is to prove that our definition of composition and compatibility enables the incremental design of systems. Before we prove this result in Theorem 8, we prove two lemmas establishing that both composite open-guarantees and composite assumptions are associative. We start with the lemma for open-guarantees and define ${\mathcal {G}}_{(F\otimes F')\otimes F''} = (Z_{F\otimes F', F''} \times Y_{F\otimes F', F''}) {\setminus } ({\mathcal {G}}_{F\otimes F'} \bullet {\mathcal {G}}_{F''})$ as the open-guarantee defined by first composing $F$ with $F'$, followed by composing the resulting interface with $F''$.

#### Lemma 6

Let $F$, $F'$ and $F''$ be interfaces with pairwise disjoint set of output variables. Then, ${\mathcal {G}}_{F\otimes (F' \otimes F'')} = {\mathcal {G}}_{(F\otimes F')\otimes F''}$.

#### Proof

Let $F$, $F'$ and $F''$ be arbitrary interfaces with pairwise disjoint set of output variables. By definition of variables between different interfaces:

$$\begin{aligned} Z_{F\otimes F', F''} \times Y_{F\otimes F', F''} = (Z_{F} \cup Z_{F'} \cup Z_{F''}) \times (Y_{F} \cup Y_{F'} \cup Y_{F''}) = Z_{F, F' \otimes F''} \times Y_{F, F' \otimes F''}. \end{aligned}$$

In what follows, we denote the set of all variables over the three interfaces as $Z$ (i.e., $Z= Z_{F} \cup Z_{F'} \cup Z_{F''}$), and the set of output variables as $Y$ (i.e., $Y= Y_{F} \cup Y_{F'} \cup Y_{F''}$). By definition of composite open-guarantees:

$$\begin{aligned} \begin{aligned} {\mathcal {G}}_{(F\otimes F')\otimes F''}&= (Z\times Y) \setminus ({\mathcal {G}}_{F\otimes F'} \bullet {\mathcal {G}}_{F''}) \text { and }\\ {\mathcal {G}}_{F\otimes (F' \otimes F'')}&= (Z\times Y) \setminus ({\mathcal {G}}_{F} \bullet {\mathcal {G}}_{F' \otimes F''}). \end{aligned} \end{aligned}$$

Then, the lemma statement is equivalent to:

$$\begin{aligned} {\mathcal {G}}_{F\otimes F'} \bullet {\mathcal {G}}_{F''}\ =\ {\mathcal {G}}_{F} \bullet {\mathcal {G}}_{F' \otimes F''}. \end{aligned}$$

We present part of the proof to illustrate how to combine properties of composite flows with Lemma 2 to prove this result. The full proof is in the appendix. We consider the case ${\mathcal {G}}_{F\otimes F'} \bullet {\mathcal {G}}_{F''} \subseteq {\mathcal {G}}_{F} \bullet {\mathcal {G}}_{F' \otimes F''}$, which is proved by induction on $n\in {\mathbb {N}}$ on the following statement:

$$\begin{aligned} \text { if }&(z,y) \in (\textrm{Id}_{Z} \cup \overline{{\mathcal {G}}}_{F''}) \circ ( \overline{{\mathcal {G}}}_{F\otimes F'} \circ \overline{{\mathcal {G}}}_{F''})^n,\\ \text {there exists}\ m \in {\mathbb {N}}\text { s.t.\ }&(z,y) \in (\textrm{Id}_{Z} \cup \overline{{\mathcal {G}}}_{F' \otimes F''}) \circ (\overline{{\mathcal {G}}}_{F} \circ \overline{{\mathcal {G}}}_{F' \otimes F''})^m \circ \textrm{Id}_{Y_{F''}}. \end{aligned}$$

We look at the induction step. We assume as induction hypothesis (IH) that the property holds for *n* and consider arbitrary (*z*, *y*) s.t. $(z,y) \in (\textrm{Id}_{Z} \cup \overline{{\mathcal {G}}}_{F''}) \circ ( \overline{{\mathcal {G}}}_{F\otimes F'} \circ \overline{{\mathcal {G}}}_{F''})^{n+1}$. To allow the application of (IH), we decompose (*z*, *y*) into two parts, $(z,y) = \{(z,s)\}\circ \{(s,y)\}$, as follows:

$$\begin{aligned} (\star )\ (z,s) \in (\textrm{Id}_{Z} \cup \overline{{\mathcal {G}}}_{F''}) \circ ( \overline{{\mathcal {G}}}_{F\otimes F'} \circ \overline{{\mathcal {G}}}_{F''})^{n} \ \text { and }\ (s,y) \in \overline{{\mathcal {G}}}_{F\otimes F'} \circ \overline{{\mathcal {G}}}_{F''}. \end{aligned}$$

By (IH), $\text {there exists } m \in {\mathbb {N}}\text { s.t.\ }(z,s) \in (\textrm{Id}_{Z} \cup \overline{{\mathcal {G}}}_{F' \otimes F''}) \circ (\overline{{\mathcal {G}}}_{F} \circ \overline{{\mathcal {G}}}_{F' \otimes F''})^m \circ \textrm{Id}_{Y_{F''}}.$ Moreover, by definition of composite guarantees ($\overline{{\mathcal {G}}}_{F\otimes F'} = {\mathcal {G}}_{F} \bullet {\mathcal {G}}_{F'}$) and Lemma 2:

$$\begin{aligned} (\star \star )\ (s,y) \in (\textrm{Id}_{Z} \cup \overline{{\mathcal {G}}}_{F}) \circ (\overline{{\mathcal {G}}}_{F'} \circ \overline{{\mathcal {G}}}_{F})^* \circ (\textrm{Id}_{Z} \cup \overline{{\mathcal {G}}}_{F'}) \circ \overline{{\mathcal {G}}}_{F''}. \end{aligned}$$

If $(z,s) \in \textrm{Id}_{Z}$, then $(z,y) = (s,y)$. By $(\star \star )$ and monotonicity of open-guarantees, $\overline{{\mathcal {G}}}_{F''}\subseteq \overline{{\mathcal {G}}}_{F' \otimes F''}$, we have $(z,y) \in (\textrm{Id}_{Z} \cup \overline{{\mathcal {G}}}_{F}) \circ (\overline{{\mathcal {G}}}_{F' \otimes F''} \circ \overline{{\mathcal {G}}}_{F})^* \circ \overline{{\mathcal {G}}}_{F' \otimes F''}.$ Which is equivalent to $(z,y) \in (\textrm{Id}_{Z} \cup \overline{{\mathcal {G}}}_{F' \otimes F''}) \circ (\overline{{\mathcal {G}}}_{F} \circ \overline{{\mathcal {G}}}_{F' \otimes F''})^*.$

For the case that $(z,s) \notin \textrm{Id}_{Z}$, we know that $s \in Y_{F''}$. By $F'$ and $F''$ having disjoint sets of output variables, definition of composite open-guarantees ($\overline{{\mathcal {G}}}_{F' \otimes F''} = {\mathcal {G}}_{F'} \bullet {\mathcal {G}}_{F''}$) and Lemma 2, we unfold the expression for (*z*, *s*) as follows:

$$\begin{aligned} (z,s) \in (\textrm{Id}_{Z} \cup \overline{{\mathcal {G}}}_{F' \otimes F''}) \circ (\overline{{\mathcal {G}}}_{F} \circ \overline{{\mathcal {G}}}_{F' \otimes F''})^{m-1} \circ \overline{{\mathcal {G}}}_{F} \circ (\textrm{Id}_{Z} \cup \overline{{\mathcal {G}}}_{F'}) \circ (\overline{{\mathcal {G}}}_{F''} \circ \overline{{\mathcal {G}}}_{F'})^* \circ \overline{{\mathcal {G}}}_{F''}. \end{aligned}$$

Then, by $(\star )$, and definition of composite open-guarantee, $(z,y) \in (\textrm{Id}_{Z} \cup \overline{{\mathcal {G}}}_{F' \otimes F''}) \circ (\overline{{\mathcal {G}}}_{F} \circ \overline{{\mathcal {G}}}_{F' \otimes F''})^{m'}$ for some $m' > m$. $\square$

In the lemma below, we prove that derived assumptions between composable interfaces are both monotonic and associative. Note that, as composite open-guarantees are commutative, then composite assumptions are also commutative.

#### Lemma 7

Let $F$, $F'$ and $F''$ be information-flow interfaces that are pairwise composable. *(a)* If $(z,z') \in {\hat{\mathcal {A}}}_{F', F''}$, then $(z,z') \in {\hat{\mathcal {A}}}_{F\otimes F', F''}$. *(b)* If $(z,z') \in {\hat{\mathcal {A}}}_{F, F' \otimes F''}$, then $(z,z') \in {\hat{\mathcal {A}}}_{F\otimes F', F''} \cup {\hat{\mathcal {A}}}_{F, F'}$.

#### Proof

Consider arbitrary information-flow interfaces $F$, $F'$ and $F''$ that are pairwise composable, i.e., all three interfaces have pairwise disjoint sets of output variables. We focus on a case of the item (b) we want to prove to illustrate how the different definitions and properties presented so far contribute to this result. The full proof is in the appendix. We consider arbitrary pair of variables $(z,z')$ and assume that $(z,z') \in {\hat{\mathcal {A}}}_{F, F' \otimes F''}$. By definition of derived assumptions, there exists a variable *s*:

$$\begin{aligned} (z,s) \in {\mathcal {A}}_{F} \cup {\mathcal {A}}_{F' \otimes F''} \text { and }(z',s) \in \overline{{\mathcal {G}}}_{F, F' \otimes F''}, \text { where } s \in \textrm{Shared}_{F, F' \otimes F''}. \end{aligned}$$

We proceed by cases on the domain of the shared variable $s\in (X_{F} \cup X_{F' \otimes F''}) \cap Y_{F, F' \otimes F''}$ and look in detail into the case $s \in X_{F' \otimes F''} \cap Y_{F}$. Given the domain of *s*, it can only be the case that $(z,s) \in {\mathcal {A}}_{F' \otimes F''}$ and we proceed by cases on ${\mathcal {A}}_{F' \otimes F''}$ definition.

If $(z,s) \in {\mathcal {A}}_{F'}$, then *s* is a shared variable between $F$ and $F'$ (i.e., $s \in X_{F'} \cap Y_{F}$). By associativity of composite open-guarantees (Lemma 6), $(z',s) \in {\mathcal {G}}_{F\otimes F'} \bullet {\mathcal {G}}_{F''}$. By output variables of all three interfaces being disjoint, $s \in Y_{F}$ and Lemma 2, then the last flow from any path from $z'$ to *s* must be in $\overline{{\mathcal {G}}}_{F\otimes F'}$. Formally, $(z',s) = (\textrm{Id}_{Z_{F\otimes F', F''}} \cup \overline{{\mathcal {G}}}_{F''}) \circ (\overline{{\mathcal {G}}}_{F\otimes F'} \circ \overline{{\mathcal {G}}}_{F''})^* \circ \overline{{\mathcal {G}}}_{F\otimes F'}$. Equivalently, there exists a variable $s'$ s.t. $(z',s) = (z',s') \cdot (s',s)$ with:

$$\begin{aligned} (z',s') \in (\textrm{Id}_{Z_{F\otimes F', F''}} \cup \overline{{\mathcal {G}}}_{F''}) \circ (\overline{{\mathcal {G}}}_{F\otimes F'} \circ \overline{{\mathcal {G}}}_{F''})^* \text { and }(s',s) \in \overline{{\mathcal {G}}}_{F\otimes F'}. \end{aligned}$$

By $(z,s) \in {\mathcal {A}}_{F'}$ and $(s',s) \in \overline{{\mathcal {G}}}_{F\otimes F'}$, then $(z,s') \in {\hat{\mathcal {A}}}_{F, F'}$. We proceed now by cases on $(z',s')$. If $(z',s') \in \textrm{Id}_{Z_{F\otimes F', F''}}$, then $(z',s) = (s',s)$ and so $(z',s) \in \overline{{\mathcal {G}}}_{F\otimes F'}$. Hence, by $(z,s) \in {\mathcal {A}}_{F'}$ and definition of derived assumption, $(z,z') \in {\hat{\mathcal {A}}}_{F, F'}$. If $(z',s') \notin \textrm{Id}_{Z_{F\otimes F', F''}}$, then $s'$ must be an output variable of $F''$ and an input variable of the other interface (i.e., $s' \in Y_{F''} \cap X_{F\otimes F'}$). Then, $(z,s') \in {\mathcal {A}}_{F\otimes F'}$. By $(z',s') \in \overline{{\mathcal {G}}}_{(F\otimes F') \otimes F''}$ and definition of derived assumptions, $(z,z') \in {\hat{\mathcal {A}}}_{F\otimes F', F''}$.

If $(z,s) \in {\hat{\mathcal {A}}}_{F', F''}$, by definition of derived assumptions there exists a shared variable $s' \in \textrm{Shared}_{F', F''}$ s.t. $(z,s') \in {\mathcal {A}}_{F'} \cup {\mathcal {A}}_{ F''} \text { and }(s,s') \in \overline{{\mathcal {G}}}_{F', F''}$. As done for the previous case, by *s* being an output variable of $F$, $(z',s) \in \overline{{\mathcal {G}}}_{F, F' \otimes F''}$ and Lemma 2, $(z',s) \in (\textrm{Id}_{Z_{F, F' \otimes F''}} \cup \overline{{\mathcal {G}}}_{F' \otimes F''}) \circ (\overline{{\mathcal {G}}}_{F} \circ \overline{{\mathcal {G}}}_{F' \otimes F''})^* \circ \overline{{\mathcal {G}}}_{F}$. Moreover, by $(s,s') \in \overline{{\mathcal {G}}}_{F', F''}$. then we know that a flow from *s* to $s'$ can be defined by an alternating composition of elements of $\overline{{\mathcal {G}}}_{F'}$ and $\overline{{\mathcal {G}}}_{F''}$, i.e. without using elements of $\overline{{\mathcal {G}}}_{F}$. Hence $(z',s) \cdot (s,s') \in \overline{{\mathcal {G}}}_{F, F' \otimes F''}$ and, by associativity of composite open-guarantees (Lemma 6), $(z',s') \in \overline{{\mathcal {G}}}_{F\otimes F', F''}$. If $(z,s') \in {\mathcal {A}}_{F'}$, then $s' \in Y_{F''}$ and, by (ii), $s' \notin Y_{F}$. Then, $s' \in X_{F\otimes F'}$ and so $(z,s') \in {\mathcal {A}}_{F\otimes F'}$. Hence, by $(z',s') \in \overline{{\mathcal {G}}}_{F\otimes F', F''}$ and $(z,s') \in {\mathcal {A}}_{F\otimes F'}$, $(z,z') \in {\hat{\mathcal {A}}}_{F\otimes F', F''}$. If $(z,s') \in {\mathcal {A}}_{F''}$, then, by $(z,s') \in \overline{{\mathcal {G}}}_{F\otimes F', F''}$, $(z,z') \in {\hat{\mathcal {A}}}_{F\otimes F', F''}$.

Lastly, if $(z,s) \in {\mathcal {A}}_{F''}$, then, by $(z',s) \in \overline{{\mathcal {G}}}_{F\otimes F', F''}$ and definition of derived assumptions, $(z,z') \in {\hat{\mathcal {A}}}_{F\otimes F', F''}$. $\square$

We have now all the necessary intermediary results to prove that information-flow interfaces support the *incremental design of systems*, i.e., different parts of a system can be deemed compatible for composition without requiring further information on the remaining system.

#### Example 7

In this example, we illustrate one of the interesting cases in the proof of incremental design. In this case, we want to prove that by assuming $(F\otimes F') \sim F''$, it follows that $F\sim (F' \otimes F'')$. And, in particular, we want to show that for all pair of variables (*z*, *s*) in the assumption of $F$ (i.e., $(z,s) \in {\mathcal {A}}_{F}$) it must be the case that $(z,s) \in {\mathcal {G}}_{F, F' \otimes F''}$. We start by assuming towards a contradiction that $(z,s) \in {\mathcal {A}}_{F} \cap \overline{{\mathcal {G}}}_{F, F' \otimes F''}$. Hence *s* is a shared variable between $F$ and $F'\otimes F''$. We elaborate now in the case that *s* is a shared variable between $F$ and $F'$, with a depiction of the interfaces referred to in the argument below.
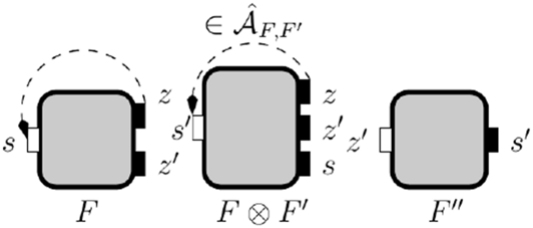


By composite flows being associative, then (*z*, *s*) must be an element of $\overline{{\mathcal {G}}}_{F\otimes F', F''}$. And, by (*z*, *s*) being an assumption of $F$ and by definition of propagated assumptions, for all input variables $s'$ that can flow to *z* through an implementation their composition (i.e., $(s',z) \in \overline{{\mathcal {G}}}_{F\otimes F'}$), then $(z,s')$ must be in the propagated assumptions of both interfaces (i.e., $(z,s') \in {\hat{\mathcal {A}}}_{F,F'}$). Formally, $(z,s') \in {\hat{\mathcal {A}}}_{F, F'}$ and, as a consequence, ${(z,s') \in {\mathcal {A}}_{F\otimes F'}}$. We illustrate this case in the figure to the left.

Finally, by the initial compatibility assumption, ${(F\otimes F') \sim F''}$, ${(z,s') \in {\mathcal {G}}_{F\otimes F', F''}}$. However, as illustrated by $F''$ above, *z* can flow to $s'$ when $F\otimes F'$ is composed with $F''$ because the information in *z* can first flow to $z'$ and then flow to $s'$. Hence ${(z,s') \in \overline{{\mathcal {G}}}_{F\otimes F', F''}}$, which contradicts our initial assumption.

#### Theorem 8

Let $F$, $F'$ and $F''$ be information-flow interfaces. If $F\sim F'$ and $(F\otimes F') \sim F''$, then $F' \sim F''$ and $F\sim (F' \otimes F'')$.

#### Proof

Consider arbitrary interfaces $F$, $F'$ and $F''$, such that (i) $F\sim F'$; and (ii) $F\otimes F' \sim F''$. Note that from our initial assumptions (iii) all three interfaces have disjoint sets of output variables.

We start by proving that $F' \sim F''$. As noted in (iii), $Y_{F'} \cap Y_{F''} = \emptyset$, i.e. $F'$ and $F''$ are composable. We are missing to prove that their assumptions are supported by the composite open-guarantees, i.e., $(({\mathcal {A}}_{F'} \cup {\mathcal {A}}_{F''}) \cap (Z_{F', F''} \times Y_{F', F''})) \subseteq {\mathcal {G}}_{F' \otimes F''}.$ Let $(z,s) \in X_{F', F''} \times Y_{F', F''}$ and $(z,s) \in {\mathcal {A}}_{F'} \cup {\mathcal {A}}_{F''}$. Note that by (i) and (ii), $s \notin Y_{F}$. We want to prove that (*z*, *s*) is in the composite open-guarantee. As $s \notin Y_{F}$ and, by definition of composite assumptions, if $(z,s) \in {\mathcal {A}}_{F'}$, then $(z,s) \in {\mathcal {A}}_{F\otimes F'}$. Then, $(z,s) \in {\mathcal {A}}_{F\otimes F'} \cup {\mathcal {A}}_{F''}$ and, by the compatibility between the interfaces (ii), $(z,s) \in {\mathcal {G}}_{F\otimes F', F''}$. By monotonicity of composite open-guarantees, $\overline{{\mathcal {G}}}_{F'\otimes F''} \subseteq \overline{{\mathcal {G}}}_{F, F'\otimes F''}$ and, by their associativity (Lemma 6), $\overline{{\mathcal {G}}}_{F'\otimes F''} \subseteq \overline{{\mathcal {G}}}_{F\otimes F', F''}$. Then, by $(z,s) \notin \overline{{\mathcal {G}}}_{F\otimes F', F''}$, we have $(z,s) \notin \overline{{\mathcal {G}}}_{F'\otimes F''}$ and, so, $(z,s) \in {\mathcal {G}}_{F'\otimes F''}$.

We prove now that $F\sim F' \otimes F''$. From (iii), $Y_{F} \cap Y_{F' \otimes F''}= \emptyset$, i.e. $F$ and $F' \otimes F''$ are composable. We are missing to prove that $(({\mathcal {A}}_{F} \cup {\mathcal {A}}_{F' \otimes F''}) \cap (X_{F, {F' \otimes F''}} \times Y_{F, {F' \otimes F''}})) \subseteq {\mathcal {G}}_{F, F' \otimes F''}.$ Consider arbitrary $(z,s) \in X_{F, {F'\otimes F''}} \times Y_{F, {F' \otimes F''}}$ s.t. $(z,s) \in {\mathcal {A}}_{F} \cup {\mathcal {A}}_{F' \otimes F''}$. We prove that $(z,s) \in {\mathcal {G}}_{F, {F' \otimes F''}}$ by cases in the (*z*, *s*) domain.

We start with the case that $(z,s) \in {\mathcal {A}}_{F}$. Then, *s* is an input variable of $F$, $s\in X_{F}$, and, by definition of information-flow interfaces, $s\notin Y_{F}$. If $s \in Y_{F'}$, then, *s* is a shared variable between $F$ and $F'$ and, by their compatibility (assumption (i)), $(\star )\ (z,s) \in {\mathcal {G}}_{F\otimes F'}$. Assume towards a contradiction that $(z,s) \notin {\mathcal {G}}_{F, {F'\otimes F''}}$. So, $(z,s) \in \overline{{\mathcal {G}}}_{F, {F' \otimes F''}}$. By associativity of composite open guarantees (Lemma 6), $(z,s) \in \overline{{\mathcal {G}}}_{F\otimes F',F''}$. By $s \in Y_{F\otimes F'}$ and Lemma 2, there exists a variable $s'$ s.t. $(z,s) = (z,s') \cdot (s',s)$ with:

$$\begin{aligned} (\star \star )\ (z,s') \in (\textrm{Id}_{Z_{F\otimes F',F''}} \cup \overline{{\mathcal {G}}}_{F''}) \circ (\overline{{\mathcal {G}}}_{F\otimes F'} \circ \overline{{\mathcal {G}}}_{F''})^* \text { and }(s',s) \in \overline{{\mathcal {G}}}_{F\otimes F'}. \end{aligned}$$

By $(z,s) \in {\mathcal {A}}_{F}$ and $(s',s) \in {\mathcal {G}}_{F\otimes F'}$, then $(z,s') \in {\hat{\mathcal {A}}}_{F,F'}$. If $(z,s') \in \textrm{Id}_{Z_{F\otimes F',F''}}$, then $z=s'$ and $(z,s) \in \overline{{\mathcal {G}}}_{F\otimes F'}$, which contradicts $(\star )$. Otherwise, $s' \in Y_{F''}$ and, by the interfaces compatibility (assumption (ii)), then $s' \in X_{F\otimes F'}$. Then, by $(z,s') \in {\hat{\mathcal {A}}}_{F,F'}$ and definition of composition, $(z,s') \in {\mathcal {A}}_{F\otimes F'}$. As $s' \in X_{F\otimes F'} \cap Y_{F''}$ and by (ii), $(z,s') \in {\mathcal {G}}_{F\otimes F',F''}$, which contradicts $(\star \star )$. Hence $(z,s) \notin \overline{{\mathcal {G}}}_{F, F' \otimes F''}$, i.e. $(z,s) \in {\mathcal {G}}_{F, F' \otimes F''}$. For the case that $(z,s) \in {\mathcal {A}}_{F}$ and $s \in Y_{F''}$, by (ii), $s \in X_{F\otimes F'}$, $(z,s) \in {\mathcal {A}}_{F\otimes F'}$ and $(z,s) \in {\mathcal {G}}_{F\otimes F', F''}$. Then, by associativity of composite flows (Lemma 6), $(z,s) \in {\mathcal {G}}_{F, F' \otimes F''}$.

When $(z,s) \in {\mathcal {A}}_{F' \otimes F''}$, then, $s\in X_{F' \otimes F''}$ and we proceed by cases on the definition of composite assumption. Note that, by the sets of output variables being disjoint, we are only interested in the cases where $s \in Y_{F}$. If $(z,s) \in {\mathcal {A}}_{F'}$, then it is analogous to the previous case where $(z,s) \in {\mathcal {A}}_{F}$ and $s\in Y_{F'}$. If $(z,s) \in {\mathcal {A}}_{F''}$, then previous case where $(z,s) \in {\mathcal {A}}_{F}$ and $s\in Y_{F''}$. Otherwise, $(z,s) \in {\hat{\mathcal {A}}}_{F',F''}$, and, by monotonicity of derived assumptions (Lemma 7), $(z,s) \in {\hat{\mathcal {A}}}_{F\otimes F',F''}$. Then, by (ii) and $s \in Y_{F\otimes F', F''}$, $(z,s) \notin \overline{{\mathcal {G}}}_{F\otimes F', F''}$, i.e., $(z,s) \notin \overline{{\mathcal {G}}}_{F, F'\otimes F''}$ (Lemma 6). $\square$

We prove now that, for compatible interfaces, composition between information-flow interfaces is associative. This is a stronger property than incremental design, as the latter only guarantees that compatibility is independent of the order in which we compose interfaces with no guarantee of the outcome of the composition itself.

#### Theorem 9

If $F\sim F'$ and $F\otimes F' \sim F''$, then $(F\otimes F') \otimes F'' = F\otimes (F' \otimes F'').$

#### Proof

Consider arbitrary interfaces $F$, $F'$ and $F''$. Assume that $F\sim F'$ and $F\otimes F' \sim F''$. Then, by Theorem 8, $(\star )$ $F' \sim F''$ and $F\sim F' \otimes F''$. By definition of composition, ($\dagger$) $X_{F\otimes F',F''} = X_{F, F' \otimes F''}$, $Y_{F\otimes F',F''} = Y_{F, F' \otimes F''}$, and $Z_{F\otimes F',F''} = Z_{F, F' \otimes F''}$. And, by Lemma 6, ${\mathcal {G}}_{F\otimes F', F''} = {\mathcal {G}}_{F, F' \otimes F''}$. Using our initial assumptions, $(\star )$, Lemma 7 and ($\dagger$), it follows: ${\mathcal {A}}_{F\otimes F',F''} ={\mathcal {A}}_{F, F' \otimes F''}.$ Then, $(\star \star )\ \hat{{\mathcal {P}}}_{{\mathcal {A}}_{F\otimes F',F''}, {\mathcal {G}}_{F\otimes F',F''}} = \hat{{\mathcal {P}}}_{{\mathcal {A}}_{F,F' \otimes F''}, {\mathcal {G}}_{F,F' \otimes F''}}$. And, by our initial assumptions and $(\star )$, it follows:

$$\begin{aligned} \begin{aligned} {\mathcal {P}}_{F\otimes F', F''}&= {\mathcal {P}}_{F} \cup {\mathcal {P}}_{F'} \cup {\mathcal {P}}_{F''} \cup \hat{{\mathcal {P}}}_{{\mathcal {A}}_{F' \otimes F''}, {\mathcal {P}}_{F' \otimes F''}} \\&\quad \cup \hat{{\mathcal {P}}}_{{\mathcal {A}}_{F, F' \otimes F''}, {\mathcal {P}}_{F,F' \otimes F''}} = {\mathcal {P}}_{F, F' \otimes F''}. \end{aligned} \end{aligned}$$

Note that by $(\star \star )$ and definition of derived closed-guarantees, $\hat{{\mathcal {P}}}_{{\mathcal {A}}_{F' \otimes F''}, {\mathcal {P}}_{F' \otimes F''}} \subseteq \hat{{\mathcal {P}}}_{{\mathcal {A}}_{F, F' \otimes F''}, {\mathcal {P}}_{F,F' \otimes F''}}$. $\square$

### Refinement and independent implementability

We now define a refinement relation between information-flow interfaces. Intuitively, an interface $F'$ refines $F$ iff $F'$ admits more environments than $F$, while possibly constraining its implementations.

#### Definition 9

An information-flow interface $F'= (X, Y, {\mathcal {A}}', {\mathcal {G}}', {\mathcal {P}}')$ *refines* $F= (X, Y, {\mathcal {A}}, {\mathcal {G}}, {\mathcal {P}})$, written $F' \preceq F$, when ${{\mathcal {A}}_{F'} \subseteq {\mathcal {A}}_{F}}$, ${\mathcal {G}}_{F} \subseteq {\mathcal {G}}_{F'}$ and ${\mathcal {P}}_{F} \subseteq {\mathcal {P}}_{F'}$.

It follows, by definition of implementations and refinement, that for all components $f$ that implement refinements of $F$, $F' \preceq F$ and $f\models _{{\mathcal {G}}'} F'$, then they are an implementation of $F$, $f\models _{{\mathcal {G}}} F$, too. Likewise, for permissible environments: for all components $f_{{\mathcal {E}}}$ that are permissible environments of $F$, $f_{{\mathcal {E}}} \models _{\!\!{\mathcal {A}}} F$, and all of $F$’s refinements, $F' \preceq F$, then the component $f_{{\mathcal {E}}}$ is permissible environment of $F'$, $f_{{\mathcal {E}}} \models _{\!\!{\mathcal {A}}'} F'$, too. We remark that the other direction of both assertions naturally does not hold. Consider the case for refinement and implementations. An interface $F'$ that refines $F$ may add new constraints on its open-guarantees. For example, it may include the pair (*x*, *y*). Then, while a component with a flow from *x* to *y* implements $F$, the same component does not implement $F'$. This is the expected behavior from the refinement process: reducing the set of implementations while permitting more environments. We show next that refinement and composition support the independent implementability property.

#### Theorem 10

For all well-formed information-flow interfaces $F_1'$, $F_1$ and $F_2$, if $F_1' \preceq F_1$ and $F_1 \sim F_2$, then $F_1' \sim F_2$ and $F_1' \otimes F_2 \preceq F_1 \otimes F_2$.

#### Proof

Consider arbitrary interfaces $F_1$, $F_1'$ and $F_2$. Assume that $F_1' \preceq F_1$ and $F_1 \sim F_2$. By $F_1' \preceq F_1$, (i) ${\mathcal {A}}_{F_1'} \subseteq {\mathcal {A}}_{F_1}$, (ii) ${\mathcal {G}}_{F_1} \subseteq {\mathcal {G}}_{F_1'}$, and (iii) ${\mathcal {P}}_{F_1} \subseteq {\mathcal {P}}_{F_1'}$.

By (i) and (ii), we can directly infer that $({\mathcal {A}}_{F_1'} \cup {\mathcal {A}}_{F_2}) \subseteq ({\mathcal {A}}_{F_1} \cup {\mathcal {A}}_{F_2})$ and $\overline{{\mathcal {G}}}_{F'_1} \subseteq \overline{{\mathcal {G}}}_{F_1}$. And, by definition of no-flows composition, ${\mathcal {G}}_{F'_1} \bullet {\mathcal {G}}_{F_2} \subseteq {\mathcal {G}}_{F_1} \bullet {\mathcal {G}}_{F_2}$. So, by definition of composite open-guarantees, ${\mathcal {G}}_{F_1\otimes F_2} \subseteq {\mathcal {G}}_{F_1' \otimes F_2}$. Finally, by $F_1 \sim F_2$ and $F_1'$ refining $F_1$, it follows $(({\mathcal {A}}_{F_1'} \cup {\mathcal {A}}_{F_2}) \cap (X_{F_1, F_2} \times Y_{F_1, F_2}))\subseteq {\mathcal {G}}_{F_1'\otimes F_2}$ and the set of output variables of $F_1'$ (note that $Y_{F_1} = Y_{F_1'}$) are disjoint from the set of output variables of $F_2$ (i.e., $Y_{F_1'} \cap Y_{F_2} = \emptyset$). Hence $F_1' \sim F_2$.

We prove now that $F_1' \otimes F_2 \preceq F_1 \otimes F_2$. First, we note that above we proved that $(\star ) \ {\mathcal {G}}_{F_1 \otimes F_2} \subseteq {\mathcal {G}}_{F_1' \otimes F_2}$. Then, by (i) and definition of derived assumptions, ${\hat{\mathcal {A}}}_{F_1',F_2} \subseteq {\hat{\mathcal {A}}}_{F_1,F_2}$. So, $(\star \star )\ {\mathcal {A}}_{F_1' \otimes F_2} \subseteq {\mathcal {A}}_{F_1 \otimes F_2}$. We are just missing to prove that composite closed-guarantees satisfy ${\mathcal {P}}_{F_1 \otimes F_2} \subseteq {\mathcal {P}}_{F_1' \otimes F_2}$. By (iii) ${\mathcal {P}}_{F_1} \cup {\mathcal {P}}_{F_2} \subseteq {\mathcal {P}}_{F_1'} \cup {\mathcal {P}}_{F_2}$. Thus, by definition of composite closed-guarantees, we still need to prove that $\hat{{\mathcal {P}}}_{{\mathcal {A}}_{F_1 \otimes F_2}, {\mathcal {G}}_{F_1 \otimes F_2}} \subseteq \hat{{\mathcal {P}}}_{{\mathcal {A}}_{F_1' \otimes F_2}, {\mathcal {G}}_{F_1' \otimes F_2}}$. By $(\star )$ and definition of derived closed-guarantees, proving our goal is equivalent to proving:

$$\begin{aligned} \begin{aligned} \forall (z,y) \in {\mathcal {G}}_{F_1 \otimes F_2}:&\text { if }(z,y) \in {\mathcal {A}}_{F_1' \otimes F_2} \bullet {\mathcal {A}}_{F_1' \otimes F_2}, \text { then } (z,y) \in {\mathcal {A}}_{F_1 \otimes F_2} \bullet {\mathcal {A}}_{F_1 \otimes F_2}. \end{aligned} \end{aligned}$$

Which, by Lemma 2 and both $F_1$ and $F_1'$ having a disjoint set of output variables from $F_2$, is equivalent to:

$$\begin{aligned} \begin{aligned}&\forall (z,y) \in {\mathcal {G}}_{F_1 \otimes F_2} \forall n \in {\mathbb {N}}: \text { if }(z,y) \in (\textrm{Id}_{Z} \cup \overline{{\mathcal {A}}}_{F_1'\otimes F_2}) \circ (\overline{{\mathcal {G}}}_{F_1'\otimes F_2} \circ \overline{{\mathcal {A}}}_{F_1'\otimes F_2})^n \circ \overline{{\mathcal {G}}}_{F_1'\otimes F_2},\\&\quad \text {then } \exists m \in {\mathbb {N}}\text { s.t.\ }(z,y) \in (\textrm{Id}_{Z} \cup \overline{{\mathcal {A}}}_{F_1\otimes F_2}) \circ (\overline{{\mathcal {G}}}_{F_1\otimes F_2} \circ \overline{{\mathcal {A}}}_{F_1\otimes F_2})^m \circ \overline{{\mathcal {G}}}_{F_1\otimes F_2}. \end{aligned} \end{aligned}$$

We can prove this statement by proving the following stronger property, where *m* is always equal to *n*:

$$\begin{aligned} \begin{aligned}&\forall (z,y) \in {\mathcal {G}}_{F_1 \otimes F_2} \forall n \in {\mathbb {N}}: \text { if }(z,y) \in (\textrm{Id}_{Z} \cup \overline{{\mathcal {A}}}_{F_1'\otimes F_2}) \circ (\overline{{\mathcal {G}}}_{F_1'\otimes F_2} \circ \overline{{\mathcal {A}}}_{F_1'\otimes F_2})^n \circ \overline{{\mathcal {G}}}_{F_1'\otimes F_2},\\&\quad \text {then } (z,y) \in (\textrm{Id}_{Z} \cup \overline{{\mathcal {A}}}_{F_1\otimes F_2}) \circ (\overline{{\mathcal {G}}}_{F_1\otimes F_2} \circ \overline{{\mathcal {A}}}_{F_1\otimes F_2})^n \circ \overline{{\mathcal {G}}}_{F_1\otimes F_2}. \end{aligned} \end{aligned}$$

Finally, the property above is equivalent to the statement below, where we change the inner implication for its contrapositive:

$$\begin{aligned} \begin{aligned}&\forall (z,y) \in {\mathcal {G}}_{F_1 \otimes F_2} \forall n \in {\mathbb {N}}: \text { if }(z,y) \notin (\textrm{Id}_{Z} \cup \overline{{\mathcal {A}}}_{F_1\otimes F_2}) \circ (\overline{{\mathcal {G}}}_{F_1\otimes F_2} \circ \overline{{\mathcal {A}}}_{F_1\otimes F_2})^n \circ \overline{{\mathcal {G}}}_{F_1\otimes F_2},\\&\quad \text {then } (z,y) \notin (\textrm{Id}_{Z} \cup \overline{{\mathcal {A}}}_{F_1'\otimes F_2}) \circ (\overline{{\mathcal {G}}}_{F_1'\otimes F_2} \circ \overline{{\mathcal {A}}}_{F_1'\otimes F_2})^n \circ \overline{{\mathcal {G}}}_{F_1'\otimes F_2}. \end{aligned} \end{aligned}$$

We proceed now by proving the statement above for all $(z,y) \in {\mathcal {G}}_{F_1 \otimes F_2}$ by natural induction over $n \in {\mathbb {N}}$. We start with the *base case* $n=0$. Consider arbitrary $(z,y) \in {\mathcal {G}}_{F_1 \otimes F_2}$ s.t. $(z,y) \notin (\overline{{\mathcal {A}}}_{F_1\otimes F_2}\circ \overline{{\mathcal {G}}}_{F_1\otimes F_2}) \cup \overline{{\mathcal {G}}}_{F_1\otimes F_2}$. If $(z,y) \notin \overline{{\mathcal {G}}}_{F_1\otimes F_2}$, then, by the domain of (*z*, *y*) and $(\star )$, $(z,y) \in {\mathcal {G}}_{F_1' \otimes F_2}$. Hence $(z,y) \notin \overline{{\mathcal {G}}}_{F_1' \otimes F_2}$. If $(z,y) \notin (\overline{{\mathcal {A}}}_{F_1\otimes F_2}\circ \overline{{\mathcal {G}}}_{F_1\otimes F_2})$, then, for all $(z,s) \in \overline{{\mathcal {A}}}_{F_1\otimes F_2}$ it must be the case that $(s,y)\notin \overline{{\mathcal {G}}}_{F_1\otimes F_2}$. And, by $s \in Z$ and $y \in Y_{F_{1}}$, we know that $(s,y) \in {\mathcal {G}}_{F_1\otimes F_2}$. Then, by ($\star$) and $(\star \star )$, for all $(z,s) \in \overline{{\mathcal {A}}}_{F_1'\otimes F_2}$ we have $(s,y)\notin \overline{{\mathcal {G}}}_{F_1'\otimes F_2}$. Thus, $(z,y) \notin \overline{{\mathcal {A}}}_{F_1'\otimes F_2}\circ \overline{{\mathcal {G}}}_{F_1'\otimes F_2}$, as well.

For the *induction step*, we assume as induction hypothesis (IH) that the statement holds for *n*. Let $(z,y) \notin (\textrm{Id}_{Z} \cup \overline{{\mathcal {A}}}_{F_1 \otimes F_2}) \circ (\overline{{\mathcal {G}}}_{F_1 \otimes F_2} \circ \overline{{\mathcal {A}}}_{F_1 \otimes F_2})^{n+1} \circ \overline{{\mathcal {G}}}_{F_1\otimes F_2}.$ By (IH), $(z,y) \notin (\textrm{Id}_{Z} \cup \overline{{\mathcal {A}}}_{F_1'\otimes F_2}) \circ (\overline{{\mathcal {G}}}_{F_1'\otimes F_2} \circ \overline{{\mathcal {A}}}_{F_1'\otimes F_2})^{n} \circ \overline{{\mathcal {G}}}_{F_1'\otimes F_2} \circ \overline{{\mathcal {A}}}_{F_1\otimes F_2} \circ \overline{{\mathcal {G}}}_{F_1\otimes F_2}.$ Then, for all $(z,s) \in (\textrm{Id}_{Z} \cup \overline{{\mathcal {A}}}_{F_1'\otimes F_2}) \circ (\overline{{\mathcal {G}}}_{F_1'\otimes F_2} \circ \overline{{\mathcal {A}}}_{F_1'\otimes F_2})^{n} \circ \overline{{\mathcal {G}}}_{F_1'\otimes F_2}$ we have $(s,y) \notin \overline{{\mathcal {A}}}_{F_1\otimes F_2} \circ \overline{{\mathcal {G}}}_{F_1\otimes F_2}$. By the same reasoning applied to the base case, then for all $(z,s) \in (\textrm{Id}_{Z} \cup \overline{{\mathcal {A}}}_{F_1'\otimes F_2}) \circ (\overline{{\mathcal {G}}}_{F_1'\otimes F_2} \circ \overline{{\mathcal {A}}}_{F_1'\otimes F_2})^{n} \circ \overline{{\mathcal {G}}}_{F_1'\otimes F_2}$ with $(s,y) \notin \overline{{\mathcal {A}}}_{F_1'\otimes F_2} \circ \overline{{\mathcal {G}}}_{F_1'\otimes F_2}$. Thus, $(z,y) \notin (\textrm{Id}_{} \cup \overline{{\mathcal {A}}}_{F_1'\otimes F_2}) \circ (\overline{{\mathcal {G}}}_{F_1'\otimes F_2} \circ \overline{{\mathcal {A}}}_{F_1'\otimes F_2})^{n+1}$ $\circ \overline{{\mathcal {G}}}_{F_1'\otimes F_2}$. Hence by definition of derived closed-guarantees, ${\mathcal {P}}_{F_1 \otimes F_2} \subseteq {\mathcal {P}}_{F_1' \otimes F_2}$. And, by definition of refinement, $F_1' \otimes F_2 \preceq F_1 \otimes F_2$. $\square$

### Semantics

Information-flow interfaces are a purely syntactical design formalism. All operators and predicates are over sets of pairs of variables specifying no-flow requirements, independent of how the systems’ implementations are modelled or flows of information can be observed. From a theoretical point of view, their syntactic nature allows us to identify problems that are transversal to frameworks that support the compositional design of security requirements. While from a practical point of view, it supports decoupling security requirements from their orthogonal semantic concerns at design time.

The most natural models for information-flow interfaces are sets of information-flow components containing all permissible environments and implementations. Note that at a semantic level, when we are reasoning about concrete implementations, we no longer need closed-guarantees, as their primary purpose is to express properties about the complete system. Building from this idea, in this section, we introduce a natural semantic interpretation for information-flow interfaces as Assume/Guarantee contracts [15, 24] that specify assumptions and guarantees as sets of flow relations. As for interfaces before, $X$ and $Y$ are disjoint sets of input and output variables, respectively, with the set of all variables being defined as $Z= X\cup Y$. An *information-flow contract* is a tuple $(X, Y, A, G)$ where $A\subseteq 2^{Z\times X}$ is a set of flow relations to input variables, called *(contract) assumption*; and $G\subseteq 2^{Z\times Y}$ is a set of flow relations to output variables, called *(contract) guarantee*.

A contract guarantee (assumption) is the set with all of contract’s implementations (permissible environments). Formally, given the information-flow contract $C= (X, Y, A, G)$, an information-flow component $(X, Y, {\mathcal {M}})$ is an implementation of $C$ iff ${\mathcal {M}}\in G$; while a component $(Y, X, {\mathcal {E}})$ is a permissible environment of $C$ iff ${\mathcal {E}}\in A$.

We start by observing that every no-flow relation ${\mathcal {N}}$ defines a set with all flow-relations allowed by ${\mathcal {N}}$, denoted by $\llbracket {\mathcal {N}} \rrbracket$, and defined as $\llbracket {\mathcal {N}} \rrbracket =\{S \text { is flow relation} \mid S \subseteq {\overline{{\mathcal {N}}}}\}$. Then, the *information-flow contract* derived of the information-flow interface $F= (X, Y, {\mathcal {A}}, {\mathcal {G}}, {\mathcal {P}})$ is $\llbracket F \rrbracket = (X, Y, \llbracket {\mathcal {A}} \rrbracket , \llbracket {\mathcal {G}} \rrbracket ).$ In this section, we are only interested in information-flow interfaces with assumptions and guarantees defined with no-flow relations. Note that, otherwise, the translation to information-flow contracts is not meaningful, as they are defined over sets of flow relations.

#### Example 8

**Fig. 11:**
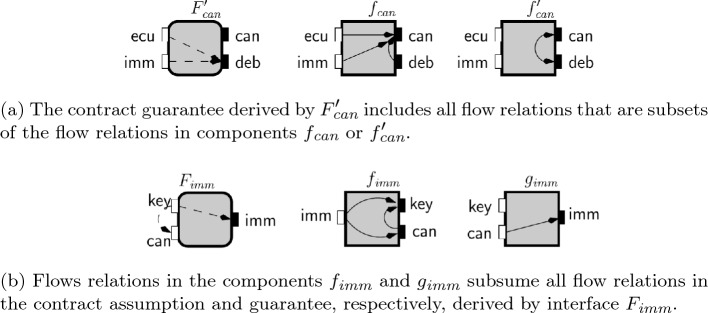
Example of components with flow relations that subsume all flow relations derived from interfaces $F'_{can }$ and $F_{imm }$

Consider the information-flow interface $F'_{can }$ in Fig. 11a and let $\llbracket F'_{can } \rrbracket = (\{\textsf {ecu},\textsf {imm}\}, \{\textsf {can}, \textsf {deb}\}, A_{can }, G_{can })$ where $A_{can }$ and $G_{can }$ are explained next. The interface $F'_{can }$ has an empty assumption, then the contract assumption $A_{can }$ contains all components with all possible flow relations to $\{\textsf {ecu},\textsf {imm}\}$. Formally, $A_{can } = \{{\mathcal {E}}\text { flow relation } \mid \ {\mathcal {E}}\subseteq \{\textsf {ecu}, \textsf {imm}, \textsf {can}, \textsf {deb}\} \times \{\textsf {ecu}, \textsf {imm}\} \}$. The components depicted in Fig. 11a are the two maximal implementations for $F'_{can }$, i.e., all other implementations refine one of them. Let ${\mathcal {M}}_{can }$ be the flow relation in $f_{can }$ and ${\mathcal {M}}'_{can }$ be the flow relation in $f'_{can }$, then the contract guarantee for $F_{can }$ is $G_{can } = \{ {\mathcal {M}}\text { flow relation } \mid {\mathcal {M}}\subseteq {\mathcal {M}}_{can } \text { or }{\mathcal {M}}\subseteq {\mathcal {M}}'_{can }\}$.

Now, consider the information-flow interface $F_{imm }$ in the Fig. 11b. Its derived contract assumption contains all refinements of the flow relation depicted in the component $f_{imm }$; while the derived contract guarantee has all flows that subsume the flow relation in $g_{imm }$. Formally, $\llbracket F_{imm } \rrbracket = (\{\textsf {key},\textsf {can}\}, \{\textsf {imm}\}, A_{imm }, G_{imm })$ where

$$\begin{aligned} \begin{aligned} A_{imm }&= \{{\mathcal {E}}\text { flow relation} \mid {\mathcal {E}}\subseteq \{(\textsf {imm},\textsf {key}), (\textsf {imm}, \textsf {can}), (\textsf {can}, \textsf {key}), (\textsf {can},\textsf {can}),(\textsf {key},\textsf {key})\} \} \\ G_{imm }&= \{{\mathcal {M}}\text { flow relation} \mid {\mathcal {M}}\subseteq \{(\textsf {can},\textsf {imm}), (\textsf {imm}, \textsf {imm})\}\}. \end{aligned} \end{aligned}$$

We will now introduce composition, compatibility, and refinement for information-flow contracts and prove their relation to the matching notions in the interface theory. We first extend the composition of flow relations to sets of flow relations straightforwardly. Let $S,S'$ be two sets of flow relations, then their composition defines the following set of flow relations:

$$\begin{aligned} S \blacksquare S' = \{ ({\mathcal {S}} \cup {\mathcal {S}}')^* \ \mid \ {\mathcal {S}} \in S \text { and }{\mathcal {S}}' \in S'\}. \end{aligned}$$

This definition is akin to the composition of no-flow relations. In particular, for two no-flow relations ${\mathcal {N}}$ and ${\mathcal {N}}'$, we have ${\mathcal {N}}\bullet {\mathcal {N}}= \{(z,z') \in {\mathcal {S}} \ \mid \ {\mathcal {S}} \in \llbracket {\mathcal {N}} \rrbracket \scriptscriptstyle \blacksquare \llbracket {\mathcal {N}} \rrbracket \}$.

When we compose two contracts, their composite guarantee is the pairwise composition of all their implementations. While their composite assumption is the set of all flow relations that allow the contracts to work together. In particular, composite assumptions is the set of environments such that their composition with any implementation of the contracts’ composite is part of a permissible environment in one of the contracts’ assumptions.

#### Definition 10

The composition of two information-flow contracts $C$ and $C'$ is defined as $C\boxtimes C' = (X_{C, C'}, Y_{C, C'}, A_{C\boxtimes C'}, G_{C\boxtimes C'})$ where $Y_{C, C'} = Y_{C} \cup Y_{C'}$, $X_{C, C'} = (X_{C} \cup X_{C'}) {\setminus } Y_{C, C'}$, $G_{C\boxtimes C'} = G_{C} \scriptscriptstyle \blacksquare G_{C'}$ and

$$\begin{aligned} \begin{aligned} A_{C\boxtimes C'}&= \{{\mathcal {E}}\subseteq Z_{C, C'} \times X_{C, C'} \mid \, {\mathcal {E}}\text { is a flow relation} \text { and }\forall {\mathcal {M}}\in G_{C\boxtimes C'} \cup \{\textrm{Id}_{X_{C, C'}}\}\\&\quad \exists {\mathcal {E}}_{C} \in A_{C}: \big (({\mathcal {E}}\circ {\mathcal {M}})\cap \ Z_{C} \times X_{C}\big ) \subseteq {\mathcal {E}}_{C} \text { and }\\&\quad \exists {\mathcal {E}}_{C'} \in A_{C'}: \big (({\mathcal {E}}\circ {\mathcal {M}}) \cap \ Z_{C'} \times X_{C'}\big ) \subseteq {\mathcal {E}}_{C'}\}. \end{aligned} \end{aligned}$$

#### Example 9

**Fig. 12:**
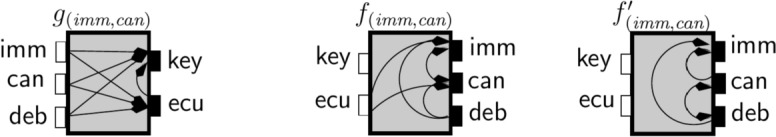
Maximal environment – $g_{imm , can }$ – and maximal implementations – $f_{imm , can }$ and $f'_{imm , can }$ – of the composition of the derived contracts of interfaces $F'_{can }$ and $F_{imm }$ in Fig. 11

Consider the flow contracts $\llbracket F'_{can } \rrbracket$ and $\llbracket F_{imm } \rrbracket$ introduced in the previous example. In Fig. 12, we depict components with flow relations that subsume all flow relations in the composition of those contracts, i.e., in $\llbracket F'_{can } \rrbracket \boxtimes \llbracket F_{imm } \rrbracket$. In particular, we denote by ${\mathcal {E}}_{(\textsf {imm},\textsf {can})}$ the flow relation defined by the component $g_{(\textsf {imm}, \textsf {can})}$, and by ${\mathcal {M}}_{(\textsf {imm}, \textsf {can})}$ and ${\mathcal {M}}'_{(\textsf {imm}, \textsf {can})}$ the flow relations defined by the components $f_{(\textsf {imm}, \textsf {can})}$ and $f'_{(\textsf {imm}, \textsf {can})}$, respectively. Then, $\llbracket F'_{can } \rrbracket \boxtimes \llbracket F_{imm } \rrbracket = (\{\textsf {key}, \textsf {ecu}\},\{\textsf {imm}, \textsf {can},\textsf {deb}\}, A_{(\textsf {imm}, \textsf {can})}, G_{(\textsf {imm},\textsf {can})})$ with:

$$\begin{aligned} \begin{aligned} A_{(\textsf {imm}, \textsf {can})}&= \{{\mathcal {E}}\text { flow relation} \mid {\mathcal {E}}\subseteq {\mathcal {E}}_{(\textsf {imm},\textsf {can})}\} \\ G_{(\textsf {imm}, \textsf {can})}&= \{{\mathcal {M}}\text { flow relation} \mid {\mathcal {M}}\subseteq {\mathcal {M}}_{(\textsf {imm}, \textsf {can})} \text { and }{\mathcal {M}}\subseteq {\mathcal {M}}'_{(\textsf {imm}, \textsf {can})}\}. \end{aligned} \end{aligned}$$

Computing the composite guarantees is straightforward. We focus now on the composite assumptions and, particularly, on the flow from $\textsf {key}$ to $\textsf {ecu}$ that is not in the flow relation of the component $g_{(\textsf {imm}, \textsf {can})}$. For this, we look into the flow relation ${\mathcal {E}}'=\{(\textsf {key},\textsf {ecu}), (\textsf {ecu}, \textsf {ecu}), (\textsf {key},\textsf {key})\}$, which is the smallest environment for $\llbracket F'_{can } \rrbracket \boxtimes \llbracket F_{imm } \rrbracket$ containing $(\textsf {key},\textsf {ecu})$. Then, according to Definition 10, ${\mathcal {E}}'$ is in the composite permissible environments, iff when composed with all possible composite implementations (i.e., all flow relations ${\mathcal {M}}\in G_{(\textsf {imm}, \textsf {can})}\cup \{(\textsf {key},\textsf {key}),(\textsf {ecu},\textsf {ecu})\}$) then the result is either part of a permissible environment of $\llbracket F'_{can } \rrbracket$ or $\llbracket F_{imm } \rrbracket$. Now, consider the flow relation ${\mathcal {M}}'=\{(\textsf {ecu},\textsf {can}),(\textsf {imm},\textsf {imm}),(\textsf {can},\textsf {can}),(\textsf {deb},\textsf {deb})\}$ that is in the composite guarantee $G_{(\textsf {imm}, \textsf {can})}$ and includes a flow from $\textsf {ecu}$ to $\textsf {can}$. Then, $(\textsf {key},\textsf {can}) \in {\mathcal {E}}' \circ {\mathcal {M}}'$ because $(\textsf {key},\textsf {can}) = (\textsf {key},\textsf {ecu})\cdot (\textsf {ecu},\textsf {can})$. By $\textsf {can}$ being an input variable of $\llbracket F_{\textsf {imm}} \rrbracket$ that has an assumption preventing a flow from $\textsf {key}$ to $\textsf {can}$, there is no permissible environment of $\llbracket F_{\textsf {imm}} \rrbracket$ containing $(\textsf {key},\textsf {can})$. Hence no flow relation with $(\textsf {key},\textsf {ecu})$ is a permissible environment of the composite.

We prove below that the contract defined by the composition of two information-flow interfaces is the same as if we first translate the interfaces to a contract separately and then compose them using the contract composition operator. This theorem allows us to transfer information-flow interface composition results to our contract theory.

#### Theorem 11

Let $F$ and $F'$ be information-flow interfaces defined with no-flow relations. $\llbracket F\otimes F' \rrbracket = \llbracket F \rrbracket \boxtimes \llbracket F' \rrbracket$.

#### Proof

Consider arbitrary information-flow interfaces $F$ and $F'$ with assumption and guarantees defined with no-flow relations. Let $\llbracket F\otimes F' \rrbracket = (X_{F, F'}, Y_{F, F'}, A_{\llbracket F\otimes F' \rrbracket }, G_{\llbracket F\otimes F' \rrbracket })$. The flow contracts $\llbracket F \rrbracket$ and $\llbracket F' \rrbracket$ have the same sets of input and output variables as the interfaces $F$ and $F'$, respectively. Then, it follows directly from definitions, that $X_{\llbracket F\otimes F' \rrbracket } = X_{\llbracket F \rrbracket \boxtimes \llbracket F' \rrbracket }$ and $Y_{\llbracket F\otimes F' \rrbracket } = Y_{\llbracket F \rrbracket \boxtimes \llbracket F' \rrbracket }$.

We start by proving that $G_{\llbracket F\otimes F' \rrbracket } \subseteq G_{\llbracket F \rrbracket } \blacksquare G_{\llbracket F' \rrbracket }$. For all flow relations ${\mathcal {M}}\in G_{\llbracket F\otimes F' \rrbracket }$, by definition of composite open-guarantees, this is equivalent to ${\mathcal {M}}\subseteq {\mathcal {G}}_{F} \bullet {\mathcal {G}}_{F'}$. Now consider ${\mathcal {M}}_{F} = {\mathcal {M}}\cap \overline{{\mathcal {G}}}_{F}$ and ${\mathcal {M}}_{F'} = {\mathcal {M}}\cap \overline{{\mathcal {G}}}_{F'}$. Then, ${\mathcal {M}}_{F}^*$ and ${\mathcal {M}}_{F'}^*$ are flow relations, and, by ${\mathcal {M}}$ being a flow relation, $({\mathcal {M}}_{F}^* \cup {\mathcal {M}}_{F'}^*)^* = ({\mathcal {M}}_{F} \cup {\mathcal {M}}_{F'})^* = {\mathcal {M}}$. So, by definition of composition of flow relations sets, ${\mathcal {M}}\in G_{\llbracket F \rrbracket } \scriptscriptstyle \blacksquare G_{\llbracket F' \rrbracket }$. To prove the other direction (i.e., $G_{\llbracket F\otimes F' \rrbracket } \supseteq G_{\llbracket F \rrbracket } \blacksquare G_{\llbracket F' \rrbracket }$), we consider arbitrary ${\mathcal {S}} \in G_{\llbracket F \rrbracket } \blacksquare G_{\llbracket F' \rrbracket }$. Then, by definition of derived contracts and flow relations set composition, there exists flow relations ${\mathcal {M}}_{F} \subseteq \overline{{\mathcal {G}}}_{F}$ and ${\mathcal {M}}_{F'} \subseteq \overline{{\mathcal {G}}}_{F'}$ s.t. ${\mathcal {S}} = ({\mathcal {M}}_{F} \cup {\mathcal {M}}_{F'})^*$. By definition of composite open-guarantees, $({\mathcal {M}}_{F} \cup {\mathcal {M}}_{F'})^* \subseteq {\mathcal {G}}_{F} \bullet {\mathcal {G}}_{F'}$. Hence $({\mathcal {M}}_{F} \cup {\mathcal {M}}_{F'})^* \in G_{\llbracket F\otimes F' \rrbracket }$.

We are missing to prove that $A_{\llbracket F\otimes F' \rrbracket } = A_{\llbracket F \rrbracket \boxtimes \llbracket F' \rrbracket }$. We start with the case $A_{\llbracket F\otimes F' \rrbracket } \subseteq A_{\llbracket F \rrbracket \boxtimes \llbracket F' \rrbracket }$ and consider an arbitrary flow relation ${\mathcal {E}}\in A_{\llbracket F\otimes F' \rrbracket }$. By definition of composite assumptions, ${\mathcal {E}}$ is a flow relation over $(Z_{F, F'}\times X_{F, F'})$ s.t. ${\mathcal {E}}\subseteq \overline{{\mathcal {A}}_{F} \cup {\mathcal {A}}_{F'} \cup {\hat{\mathcal {A}}}_{F, F'}}$. Note that by ${\mathcal {E}}\subseteq \overline{{\hat{\mathcal {A}}}_{F,F'}}$, then, for all $(z,z') \in {\mathcal {E}}$, $(z,z') \notin {\hat{\mathcal {A}}}_{F, F'}$, and, by definition of derived assumptions,:

$$\begin{aligned} (\star \star ) \text { for all } (z',s) \in \overline{{\mathcal {G}}}_{F\otimes F'},\text { then } (z,s) \notin {\mathcal {A}}_{F} \cup {\mathcal {A}}_{F'}. \end{aligned}$$

By definition of composition between contracts, we are missing prove that for all flow relations ${\mathcal {M}}\in G_{\llbracket F\otimes F' \rrbracket } \cup \{\textrm{Id}_{X_{F, F'}}\}$:

$$\begin{aligned} \begin{aligned} (\dagger )&\exists {\mathcal {E}}_{F} \in A_{\llbracket F \rrbracket }: \big (({\mathcal {E}}\circ {\mathcal {M}})\cap \ Z_{F} \times X_{F}\big ) \subseteq {\mathcal {E}}_{F} \text { and }\\&\exists {\mathcal {E}}_{F'} \in A_{\llbracket F' \rrbracket }: \big (({\mathcal {E}}\circ {\mathcal {M}})\ \cap \ Z_{F'} \times X_{F'}\big ) \subseteq {\mathcal {E}}_{F'}. \end{aligned} \end{aligned}$$

Assume that ${\mathcal {M}}= \textrm{Id}_{X_{F, F'}}$, then the condition $(\dagger )$ follows from our assumption that ${\mathcal {E}}$ is a flow relation and ${\mathcal {E}}\subseteq \overline{{\mathcal {A}}}_{F} \cap \overline{{\mathcal {A}}}_{F'}$. Consider now arbitrary ${\mathcal {M}}\in G_{\llbracket F\otimes F' \rrbracket }$ and, assume towards a contradiction that (a) for all ${\mathcal {E}}_{F} \in A_{\llbracket F \rrbracket }$ we have $\big (({\mathcal {E}}\circ {\mathcal {M}})\cap \ Z_{F} \times X_{F}\big ) \not \subseteq {\mathcal {E}}_{F}$. Let $(z,s) \in ({\mathcal {E}}\circ {\mathcal {M}})\cap \ Z_{F} \times X_{F}$. Then, (*z*, *s*) can be defined by the composition of two pairs of variables, $(z,s) = (z,z') \cdot (z',s)$, where $(z,z') \in {\mathcal {E}}$ and $(z',s) \in {\mathcal {M}}$. By $(z',s) \in {\mathcal {M}}$ and ${\mathcal {M}}\in G_{\llbracket F\otimes F' \rrbracket }$, then $(z',s)$ must be an allowed flow by the composition, i.e., $(z',s) \in \overline{{\mathcal {G}}}_{F\otimes F'}$. By $(\star \star )$, it follows that $(z,s) \notin {\mathcal {A}}_{F} \cup {\mathcal {A}}_{F'}$. As $(z,s) \in Z_{F} \times X_{F}$, then, by $(z,s) \notin {\mathcal {A}}_{F}$, there exists ${\mathcal {E}}_{F} = \{(z,s)\} \cup \textrm{Id}_{X_{F}}$ that is a flow relation s.t. ${\mathcal {E}}_{F} \subseteq \overline{{\mathcal {A}}}_{F}$, i.e., ${\mathcal {E}}_{F}\in A_{\llbracket F \rrbracket }$. This contradicts assumption (a).

We prove analogously for the case of ${\mathcal {E}}_{F'} \in A_{\llbracket F' \rrbracket }$. We prove now that $A_{\llbracket F \rrbracket \boxtimes \llbracket F' \rrbracket } \subseteq A_{\llbracket F\otimes F' \rrbracket }$. Let ${\mathcal {E}}$ be an arbitrary flow relation over $Z_{C, C'} \times X_{C, C'}$ that is an element of $A_{\llbracket F \rrbracket \boxtimes \llbracket F' \rrbracket }$, i.e., (b) ${\mathcal {E}}\in A_{\llbracket F \rrbracket \boxtimes \llbracket F' \rrbracket }$. Assume towards a contradiction that ${\mathcal {E}}$ is not an element of $A_{\llbracket F\otimes F' \rrbracket }$, i.e., ${\mathcal {E}}\not \subseteq \overline{{\mathcal {A}}}_{F\otimes F'}$. Then, by definition of composite assumptions, there exists $(z,z') \in {\mathcal {E}}$ s.t. $(z,z') \in {\mathcal {A}}_{F} \cup {\mathcal {A}}_{F'} \cup {\hat{\mathcal {A}}}_{F,F'}$. Consider the case that $(z,z') \in {\mathcal {A}}_{F}$ and let ${\mathcal {M}}= \textrm{Id}_{X_{F,F'}}$. Then, $({\mathcal {E}}\circ {\mathcal {M}}) = {\mathcal {E}}$, and for all flow relations ${\mathcal {E}}_{F} \subseteq \overline{{\mathcal {A}}}_{F}$ allowed by the assumptions of $F$, then ${\mathcal {E}}\cap (Z_{F, F'} \times X_{F,F'}) \not \subseteq {\mathcal {E}}_{F}$ due to $(z,z') \in {\mathcal {A}}_{F} \cap {\mathcal {E}}$. This contradicts our assumption (b), by definition of composite assumptions between contracts. The same reasoning applies for the case that $(z,z') \in {\mathcal {A}}_{F'}$. Finally, consider the case that $(z,z') \in {\hat{\mathcal {A}}}_{F, F'}$. By definition of derived assumptions, there exists $(z,s) \in {\mathcal {A}}_{F} \cup {\mathcal {A}}_{F'}$ and $(z',s) \in \overline{{\mathcal {G}}}_{F\otimes F'}$. As $(z',s) \in \overline{{\mathcal {G}}}_{F\otimes F'}$, then there exists a flow relation ${\mathcal {M}}\subseteq \overline{{\mathcal {G}}}_{F\otimes F'}$ s.t. $(z',s) \in {\mathcal {M}}$. Then, by $(z,z') \in {\mathcal {E}}$ and $(z',s) \in {\mathcal {M}}$, $(z,s) \in ({\mathcal {E}}\circ {\mathcal {M}})$. And, by $(z,s) \in {\mathcal {A}}_{F} \cup {\mathcal {A}}_{F'}$, it follows that there are no flow relations allowed by the assumptions of both interfaces that satisfy the condition in assumption (b). Formally, for all ${\mathcal {E}}_{{\mathcal {M}}} \subseteq \overline{{\mathcal {A}}}_{F}$ and ${\mathcal {E}}_{{\mathcal {M}}'} \subseteq \overline{{\mathcal {A}}}_{F'}$, then $\big (({\mathcal {E}}\circ {\mathcal {M}})\cap \ Z_{F} \times X_{F}\big ) \not \subseteq {\mathcal {E}}_{F}$ and $\big (({\mathcal {E}}\circ {\mathcal {M}})\cap \ Z_{F'} \times X_{F'}\big ) \not \subseteq {\mathcal {E}}_{F'}$. And this contradicts (b). $\square$

Two information-flow contracts $C$ and $C'$ are compatible if when one of them provides inputs to the other, then flows to shared variables in implementations of one of the contracts are in the permissible environments of the other contract.

#### Definition 11

Two information-flow contracts $C$ and $C'$ are compatible, denoted $C\approx C'$, iff they are composable, i.e., their output variables are disjoint, $Y_{C} \cap Y_{C'} = \emptyset$; and their implemented flows related to shared variables are in the permissible environments of the respective contract:

$$\begin{gathered} \forall {\mathcal{M}} \in (G_{C} {\blacksquare }G_{{C^{\prime}}} )\;\exists {\rm E}_{C} \in A_{C} :{\mathcal{M}} \cap (Z_{C} \times X_{C} ) \subseteq {\rm E}_{C} {\text{ and}} \hfill \\ \quad \quad \quad \quad \quad \quad \quad \exists {\rm E}_{{C^{\prime}}} \in A_{{C^{\prime}}} :{\mathcal{M}} \cap (Z_{{C^{\prime}}} \times X_{{C^{\prime}}} ) \subseteq {\rm E}_{{C^{\prime}}} . \hfill \\ \end{gathered}$$

We prove that checking the compatibility between information-flow interfaces is equivalent to checking the compatibility between the contracts derived by the given interfaces.

#### Theorem 12

Let $F$ and $F'$ be information-flow interfaces defined with no-flow relations. $F\sim F'$ iff $\llbracket F \rrbracket \approx \llbracket F' \rrbracket .$

#### Proof

We start by proving the $\Rightarrow$-direction of the statement. Assume that $F\sim F'$. Then, by definition of compatibility, $({\mathcal {A}}_{F} \cup {\mathcal {A}}_{F'}) \cap (Z_{F, F'} \times Y_{F, F'})\subseteq {\mathcal {G}}_{F\otimes F'}.$ To prove that $\llbracket F \rrbracket \approx \llbracket F' \rrbracket$, we start by considering an arbitrary flow relation ${\mathcal {M}}\in G_{\llbracket F \rrbracket } \blacksquare G_{\llbracket F' \rrbracket }$. Then, by definition of derived contract and no-flows composition, ${\mathcal {M}}\subseteq {\mathcal {G}}_{F} \bullet {\mathcal {G}}_{F'}$. By definition of composite guarantees, ${\mathcal {M}}\subseteq (Z_{F, F'} \times Y_{F, F'}) {\setminus } {\mathcal {G}}_{F\otimes F'}$ and, by our initial assumption, ${\mathcal {M}}\subseteq (Z_{F, F'} \times Z_{F, F'}) {\setminus } ({\mathcal {A}}_{F} \cup {\mathcal {A}}_{F'})$. Then, for ${\mathcal {M}}_{F} = {\mathcal {M}}\cap (Z_{F} \times X_{F})$ and ${\mathcal {M}}_{F'} = {\mathcal {M}}\cap (Z_{F'} \times X_{F'})$, it follows that ${\mathcal {M}}_{F} \subseteq \overline{{\mathcal {A}}}_{F}$ and ${\mathcal {M}}_{F'} \subseteq \overline{{\mathcal {A}}}_{F'}$.

For the $\Leftarrow$-direction, assume that $\llbracket F \rrbracket \approx \llbracket F' \rrbracket$, and assume towards a contradiction that (a) $({\mathcal {A}}_{F} \cup {\mathcal {A}}_{F'}) \cap (Z_{F, F'} \times Y_{F, F'})\not \subseteq {\mathcal {G}}_{F\otimes F'}.$ Consider arbitrary $(z,z') \in ({\mathcal {A}}_{F} \cup {\mathcal {A}}_{F'}) \cap (Z_{F, F'} \times Y_{F, F'})$ s.t. $(z,z') \notin {\mathcal {G}}_{F\otimes F'}$. Let ${\mathcal {M}}= \{(z,z')\} \cup \textrm{Id}_{Y_{F, F'}}$, which clearly is a flow relation. By $(z,z') \notin {\mathcal {G}}_{F\otimes F'}$, then ${\mathcal {M}}\in G_{\llbracket F\otimes F' \rrbracket }$ and, by $G_{\llbracket F\otimes F' \rrbracket } = G_{\llbracket F \rrbracket } \blacksquare G_{\llbracket F' \rrbracket }$ proved in Theorem 11, ${\mathcal {M}}\in G_{\llbracket F \rrbracket } \blacksquare G_{\llbracket F' \rrbracket }$. By $\llbracket F \rrbracket \approx \llbracket F' \rrbracket$, then ${\mathcal {M}}\cap (Z_{F} \times X_{F}) \subseteq \overline{{\mathcal {A}}}_{F}$ and ${\mathcal {M}}\cap (Z_{F'} \times X_{F'}) \subseteq \overline{{\mathcal {A}}}_{F'}$. So, $(z,z') \notin {\mathcal {A}}_{F} \cup {\mathcal {A}}_{F'}$, which contradicts (a). $\square$

The definition of refinement is straightforward, as it may allow more environments and fewer implementations.

#### Definition 12

An information-flow contract $C' = (X, Y, A', G')$ refines the contract $C=(X, Y, A, G)$, denoted $C' \le C$, iff $A \subseteq A'$ and $G' \subseteq G$.

We prove below that, for two given interfaces, the contract refinement over their derived contracts is equivalent to the refinement over the interfaces (modulo the closed-guarantees being a refinement already).

#### Theorem 13

Let $F$ and $F'$ be information-flow interfaces. If $F' \preceq F$, then $\llbracket F' \rrbracket \le \llbracket F \rrbracket$. And, if ${\mathcal {P}}_{F} \subseteq {\mathcal {P}}_{F'}$ and $\llbracket F' \rrbracket \le \llbracket F \rrbracket$, then $F' \preceq F$.

#### Proof

Let $F$ and $F'$ be two interfaces. Consider first that $F' \preceq F$ Then, ${\mathcal {A}}_{F'} \subseteq {\mathcal {A}}_{F}$ and ${\mathcal {G}}_{F} \subseteq {\mathcal {G}}_{F'}$, and so:

$$\begin{aligned} \begin{aligned} \{{\mathcal {E}}\text { is flow relation}\, \mid \,{\mathcal {E}}\subseteq \overline{{\mathcal {A}}}_{F}\}&\subseteq \{{\mathcal {E}}\text { is flow relation}\, \mid \,{\mathcal {E}}\subseteq \overline{{\mathcal {A}}}_{F'}\}\\ \{{\mathcal {M}}\text { is flow relation}\, \mid \,{\mathcal {M}}\subseteq \overline{{\mathcal {G}}}_{F'}\}&\subseteq \{{\mathcal {M}}\text { is flow relation}\, \mid \,{\mathcal {M}}\subseteq \overline{{\mathcal {G}}}_{F}\}. \end{aligned} \end{aligned}$$

Hence $A_{\llbracket F \rrbracket } \subseteq A_{\llbracket F' \rrbracket }$ and $G_{\llbracket F' \rrbracket } \subseteq G_{\llbracket F \rrbracket }$. For the other case, with ${\mathcal {P}}_{F} \subseteq {\mathcal {P}}_{F'}$ and $\llbracket F' \rrbracket \le \llbracket F \rrbracket$, it follows from definitions that ${\mathcal {A}}_{F'} \subseteq {\mathcal {A}}_{F}$ and ${\mathcal {G}}_{F} \subseteq {\mathcal {G}}_{F'}$. Then, $F' \preceq F$. $\square$

From Theorem 11 and Proposition 13, it follows that information-flow contracts derived from an information-flow interface satisfy both incremental design and independent implementability.

An important difference between information-flow interfaces and contracts is that while interfaces organize a system’s design and verification tasks, contracts enable reasoning about interface implementations and environments, focusing on the different parts of the system design. We include closed-guarantees in our interfaces to enable the specification of global no-flow requirements, which are enforced by a combination of open-guarantees and assumptions (the objects of interest in information-flow contracts). The proposition below illustrates the role of the closed-guarantees as a higher-level guarantee for an information-flow contract. In particular, we prove that the closed system defined by an interface (i.e., the composition of all of its derived contract’s environments with all of the derived contract’s implementations) refines the contract derived from the interface’s closed-guarantee.

#### Proposition 14

A stateless information-flow interface $F= (X, Y, {\mathcal {A}}, {\mathcal {G}}, {\mathcal {P}})$, with $\llbracket F \rrbracket = (X, Y, A_{\llbracket F \rrbracket }, G_{\llbracket F \rrbracket })$, is well-formed, if $(C_{{\mathcal {G}}}\boxtimes C_{{\mathcal {A}}})\le C_{{\mathcal {P}}}$, where $C_{{\mathcal {P}}} = (\emptyset , X\cup Y, \emptyset , \{{\mathcal {M}}\text { is flow relation}\, \mid \,{\mathcal {M}}\subseteq {\overline{{\mathcal {P}}}}_{F}\})$, $C_{{\mathcal {G}}} = (X, Y, \emptyset , G_{\llbracket F \rrbracket })$ and $C_{{\mathcal {A}}} = (Y, X, \emptyset , A_{\llbracket F \rrbracket })$.

#### Proof

Follows from definition of contract composition, refinement and Proposition 1. $\square$

## Stateful information-flow interfaces

We extend our theory with stateful components and interfaces. These are transition systems in which each state is a stateless component or interface, respectively. It is important to note that a stateless interface specifies requirements a system needs to satisfy over the time the interface specification is required. Stateful interfaces formalize how to transition between different specification states. Thus, each state in the stateful interface matches a specification state (specified with a stateless interface), where states are not necessarily in a 1-to-1 relation with single steps in a system execution.

### Definition 13

Let $X$ and $Y$ be disjoint sets of *input* and *output* variables, respectively, with $Z= X\cup Y$ the set of all variables. Let $Q$ be a set of states with $\hat{q}\in Q$ being the initial state and $\delta : Q\rightarrow 2^{Q}$ be a transition relation. A *stateful information-flow component* $\mathbbm {f}$ is a tuple $(X,Y,Q, \hat{q}, \delta , {\mathbb {M}})$, where ${\mathbb {M}}: Q\rightarrow 2^{Z\times Y}$ is a state labeling such that for all states $q\in Q$, ${\mathbb {M}}(q)$ defines a flow relation. We denote by $\mathbbm {f}(q) = (X, Y, {\mathbb {M}}(q))$ the stateless component implied by the labeling of *q*. A *stateful information-flow interface* ${\mathbb {F}}$ is a tuple $(X,Y,Q, \hat{q}, \delta , {\mathbb {A}},{\mathbb {G}}, {\mathbb {P}})$, where ${\mathbb {A}}: Q\rightarrow 2^{Z\times X}$ is called *assumption*; ${\mathbb {G}}: Q\rightarrow 2^{Z\times Y}$ is called *open-guarantee*; and ${\mathbb {P}}: Q\rightarrow 2^{Z\times Y}$ is called *closed-guarantee*. For each state $q\in Q$ we denote by ${\mathbb {F}}(q) = (X, Y, {\mathbb {A}}(q), {\mathbb {G}}(q), {\mathbb {P}}(q))$ the stateless interface defined by the assumption, open-guarantee and closed-guarantee of $q$.

A *stateful interface* ${\mathbb {F}}$ is well-formed iff ${\mathbb {F}}(\hat{q})$ is a well-formed stateless interface, and for all states $q\in Q$ reachable from the intial state $\hat{q}$ the stateless interface defined by the state $q$, ${\mathbb {F}}(q)$, is well-formed. In what follows, ${\mathbb {F}}= (X,Y,Q, \hat{q}, \delta , {\mathbb {A}},{\mathbb {G}}, {\mathbb {P}})$ and ${\mathbb {F}}' = (X',Y',Q', \hat{q}', \delta ', {\mathbb {A}}',{\mathbb {G}}', {\mathbb {P}}')$ are stateful interfaces, and $\mathbbm {f}= (X,Y,Q_{\mathbbm {f}}, \hat{q}_{\mathbbm {f}}, \delta _{\mathbbm {f}}, {\mathbb {M}})$ and $\mathbbm {f}_{{\mathcal {E}}} = (Y, X, Q_{{\mathcal {E}}}, \hat{q}_{{\mathcal {E}}}, \delta _{{\mathcal {E}}}, {\mathbb {E}})$ are stateful components.

A stateful component $\mathbbm {f}$ implements a stateful interface ${\mathbb {F}}$ if there exists a simulation relation from $\mathbbm {f}$ to ${\mathbb {F}}$ such that the stateless components in the relation implement the stateless interfaces they are related to. Permissible environments require a simulation relation from the interface they are permissible on to them.

### Definition 14

Let ${\mathbb {F}}= (X,Y,Q, \hat{q}, \delta , {\mathbb {A}},{\mathbb {G}}, {\mathbb {P}})$ be a stateful information-flow interface. A component $\mathbbm {f}= (X,Y,Q_{\mathbbm {f}}, \hat{q}_{\mathbbm {f}}, \delta _{\mathbbm {f}}, {\mathbb {M}})$ *implements* the interface ${\mathbb {F}}$, denoted by $\mathbbm {f}\models _{{\mathbb {G}}} {\mathbb {F}}$, iff there exists $H\subseteq Q_\mathbbm {f}\times Q$ s.t. $(\hat{q}_\mathbbm {f}, \hat{q}) \in H$ and for all $(q_\mathbbm {f}, q) \in H$:

- $$\mathbbm {f}(q_\mathbbm {f}) \models _{{\mathbb {G}}(q)} {\mathbb {F}}(q)$$ with $${\mathbb {F}}(q) = (X, Y, {\mathbb {A}}(q), {\mathbb {G}}(q), {\mathbb {P}}(q))$$; and
- if $q_\mathbbm {f}' \in \delta _\mathbbm {f}(q_\mathbbm {f})$, then there exists a state $q' \in \delta (q)$ s.t. $(q_\mathbbm {f}', q') \in H$.

A component $\mathbbm {f}_{{\mathcal {E}}} = (Y, X, Q_{{\mathcal {E}}}, \hat{q}_{{\mathcal {E}}}, \delta _{{\mathcal {E}}}, {\mathbb {E}})$ is an *permissible environment* for the interface ${\mathbb {F}}$, denoted by $\mathbbm {f}_{{\mathcal {E}}} \models _{\!\!{\mathbb {A}}} {\mathbb {F}}$, iff there exists a relation $H\subseteq Q\times Q_{\mathcal {E}}$ s.t. $(\hat{q}, \hat{q}_{\mathcal {E}}) \in H$ and for all $(q, q_{\mathcal {E}}) \in H$:

- $$\mathbbm {f}(q_{\mathcal {E}}) \models _{\!\!{\mathbb {A}}(q)} {\mathbb {F}}(q)$$ with $${\mathbb {F}}(q) = (X, Y, {\mathbb {A}}(q), {\mathbb {G}}(q), {\mathbb {P}}(q))$$; and
- if $q' \in \delta _{\mathbb {F}}(q)$, then there exists a state $q_{\mathcal {E}}' \in \delta _{\mathcal {E}}(q_{\mathcal {E}})$ s.t. $(q',q_{\mathcal {E}}') \in H$.

As for stateless interfaces, a well-formed stateful interface guarantees that its closed-guarantee holds under the composition between any of its implementations $\mathbbm {f}$ with any of its permissible environments $\mathbbm {f}_{{\mathcal {E}}}$.

### Proposition 15

For all well-formed interfaces ${\mathbb {F}}$, and all relations $H\subseteq Q_{\mathbbm {f}} \times Q$ and $H_{{\mathcal {E}}} \subseteq Q\times Q_{{\mathcal {E}}}$ that witness $\mathbbm {f}\models _{{\mathbb {G}}} {\mathbb {F}}$ and $\mathbbm {f}_{{\mathcal {E}}} \models _{\!\!{\mathbb {A}}} {\mathbb {F}}$, respectively, it holds:

- $$({\mathbb {M}}(\hat{q}_\mathbbm {f}) \cup {\mathbb {E}}(\hat{q}_{\mathcal {E}}))^* \cap {\mathbb {P}}(\hat{q}) = \emptyset$$; and
- for all $q\in Q$ that are reachable from $\hat{q}$, if $(q_\mathbbm {f}, q) \in H$ and $(q, q_{\mathcal {E}}) \in H_{{\mathcal {E}}}$, then $({\mathbb {M}}(q_\mathbbm {f}) \cup {\mathbb {E}}(q_{\mathcal {E}}))^* \cap {\mathbb {P}}(q) = \emptyset .$

### Proof

Consider arbitrary well-formed interface ${\mathbb {F}}= (X,Y,Q, \hat{q}, \delta , {\mathbb {A}},{\mathbb {G}}, {\mathbb {P}})$, and components $\mathbbm {f}= (X,Y,Q_{\mathbbm {f}}, \hat{q}_{\mathbbm {f}}, \delta _{\mathbbm {f}}, {\mathbb {M}})$ and $\mathbbm {f}_{{\mathcal {E}}} = (Y, X, Q_{{\mathcal {E}}}, \hat{q}_{{\mathcal {E}}}, \delta _{{\mathcal {E}}}, {\mathbb {E}})$. We assume that (i) $\mathbbm {f}\models _{{\mathbb {G}}} {\mathbb {F}}$ and $H\subseteq Q_{\mathbbm {f}} \times Q$ witnesses it; and (ii) $\mathbbm {f}_{{\mathcal {E}}} \models _{\!\!{\mathbb {A}}} {\mathbb {F}}$ and $H_{{\mathcal {E}}} \subseteq Q\times Q_{{\mathcal {E}}}$ is a relation witnessing it. Item (a) follows from Proposition 1 for stateless interfaces. For the item (b), consider arbitrary state $q\in Q$ that is reachable from the initial state $\hat{q}$. Additionally, consider arbitrary $q_\mathbbm {f}$ and $q_{\mathcal {E}}$ s.t. $(q_\mathbbm {f}, q) \in H\text { and }(q, q_{\mathcal {E}}) \in H_{{\mathcal {E}}}$. By our initial assumptions (i) and (ii), $\mathbbm {f}(q_\mathbbm {f}) \models _{{\mathbb {G}}(q)} {\mathbb {F}}(q)$ and $\mathbbm {f}_{{\mathcal {E}}}(q_{\mathcal {E}}) \models _{{\mathbb {A}}(q)} {\mathbb {F}}(q)$. By ${\mathbb {F}}$ being well-formed and by $q$ being accessible from the initial state $\hat{q}$, then ${\mathbb {F}}(q)$ is a well-formed (stateless) interface. Hence, by Proposition 1 for stateless interfaces, it follows that $({\mathbb {M}}(q_\mathbbm {f}) \cup {\mathbb {E}}(q_{\mathcal {E}}))^* \cap {\mathbb {P}}(q) = \emptyset$. $\square$

### Composition and incremental design

We compose stateful interfaces (components) as a synchronous product between the interfaces (components) in their states. For interfaces, we only keep the states defined by the composition of two compatible stateless interfaces.

#### Definition 15

Let ${\mathbb {F}}= (X,Y,Q, \hat{q}, \delta , {\mathbb {A}},{\mathbb {G}}, {\mathbb {P}})$ and ${\mathbb {F}}= (X',Y',Q', \hat{q}', \delta ', {\mathbb {A}}',{\mathbb {G}}', {\mathbb {P}}')$ be stateful information-flow interfaces. Their *composition* is defined as the tuple: ${\mathbb {F}}\otimes {\mathbb {F}}' = (X_{{\mathbb {F}}, {\mathbb {F}}'}, Y_{{\mathbb {F}}, {\mathbb {F}}'},Q_{{\mathbb {F}}, {\mathbb {F}}'}, \hat{q}_{{\mathbb {F}}, {\mathbb {F}}'}, \delta _{{\mathbb {F}}, {\mathbb {F}}'}, {\mathbb {A}}_{{\mathbb {F}}, {\mathbb {F}}'}, {\mathbb {G}}_{{\mathbb {F}}, {\mathbb {F}}'},{\mathbb {P}}_{{\mathbb {F}}, {\mathbb {F}}'} ),$ where: $\hat{q}_{{\mathbb {F}}, {\mathbb {F}}'} = (\hat{q}, \hat{q}')$ and $Q_{{\mathbb {F}}, {\mathbb {F}}'} = \{\hat{q}_{{\mathbb {F}}, {\mathbb {F}}'}\} \cup \{(q,q') \mid \ {\mathbb {F}}(q) \sim {\mathbb {F}}'(q')\}$; $(q_2, q_2') \in \delta _{{\mathbb {F}}, {\mathbb {F}}'}(q_1, q'_1)$ iff $q_2 \in \delta (q_1)$ and $q_2' \in \delta '(q_1')$; assumption and guarantees are defined by the stateless composition of their respective states, formally for all $(q, q') \in Q_{{\mathbb {F}}, {\mathbb {F}}'}$ we have $(X_{{\mathbb {F}}, {\mathbb {F}}'},Y_{{\mathbb {F}}, {\mathbb {F}}'},{\mathbb {A}}(q, q'),{\mathbb {G}}(q, q'), {\mathbb {P}}(q, q')) = {\mathbb {F}}(q) \otimes {\mathbb {F}}'(q')$.

In the proposition below, we prove that the composition of implementations of two given stateful information-flow interfaces is an implementation of the composition of the given interfaces. In the proof below, we show how to define the relation witnessing the implements relation for the composition from any witness relation from two implementations. We then use the Proposition 3 for stateless interfaces to prove that the implements relation holds for each pair in the witness relation.

#### Proposition 16

Let ${\mathbb {F}}$ and ${\mathbb {F}}'$ be stateful information-flow interfaces with open-guarantees ${\mathbb {G}}$ and ${\mathbb {G}}'$, respectively. If $\mathbbm {f}\models _{{\mathbb {G}}} {\mathbb {F}}$ and $\mathbbm {f}' \models _{{\mathbb {G}}} {\mathbb {F}}'$, then $\mathbbm {f}\otimes \mathbbm {f}' \models _{{\mathbb {G}}_{{\mathbb {F}}, {\mathbb {F}}'}} {\mathbb {F}}\otimes {\mathbb {F}}'$, where ${\mathbb {G}}_{{\mathbb {F}}, {\mathbb {F}}'}$ is the composite open-guarantee of ${\mathbb {F}}$ and ${\mathbb {F}}'$.

#### Proof

Assume that: (i) $\mathbbm {f}\models _{{\mathbb {G}}} {\mathbb {F}}$ and (ii) $\mathbbm {f}' \models _{{\mathbb {G}}} {\mathbb {F}}'$. Then, there exists $H_\mathbbm {f}$ and $H_{\mathbbm {f}'}$ that witnesses (i) and (ii), respectively. Consider the relation: $H= \{((q_\mathbbm {f}, q_{\mathbbm {f}'}), (q_{\mathbb {F}}, q_{{\mathbb {F}}'})) \ \mid \ q_{\mathbb {F}}\in H_\mathbbm {f}(q_\mathbbm {f}) \text { and }q_{{\mathbb {F}}'} \in H_{\mathbbm {f}'}(q_{\mathbbm {f}'})\}.$ Clearly, by (i) and (ii), $((\hat{q}_\mathbbm {f}, \hat{q}_{\mathbbm {f}'}), (\hat{q}_{\mathbb {F}}, \hat{q}_{{\mathbb {F}}'})) \in H$. Then, $\mathbbm {f}(\hat{q}_\mathbbm {f}) \models _{{\mathbb {G}}(\hat{q}_{\mathbb {F}})} {\mathbb {F}}(\hat{q}_{\mathbb {F}})$ and $\mathbbm {f}(\hat{q}_{\mathbbm {f}'}) \models _{{\mathbb {G}}'(\hat{q}_{{\mathbb {F}}'})} {\mathbb {F}}'(\hat{q}_{{\mathbb {F}}'})$. So, by Proposition 3 for stateless interfaces, it follows that $\mathbbm {f}(\hat{q}_\mathbbm {f}) \otimes \mathbbm {f}'(\hat{q}_{\mathbbm {f}'}) \models _{{\mathbb {G}}_{{\mathbb {F}},{\mathbb {F}}'}(\hat{q}_{{{\mathbb {F}},{\mathbb {F}}'}})} {\mathbb {F}}(\hat{q}_{\mathbb {F}}) \otimes {\mathbb {F}}'(\hat{q}_{{\mathbb {F}}'})$. Consider arbitrary $((q_\mathbbm {f}, q_{\mathbbm {f}'}), (q_{\mathbb {F}}, q_{{\mathbb {F}}'})) \in H$. Then, by (i) and (ii), there exists $({\overline{q}}_\mathbbm {f}, {\overline{q}}_{\mathbb {F}}) \in H_\mathbbm {f}$ s.t. $\mathbbm {f}({\overline{q}}_\mathbbm {f}) \models _{{\mathbb {G}}({\overline{q}}_{\mathbb {F}})} {\mathbb {F}}({\overline{q}}_{\mathbb {F}})$, and there exists $({\overline{q}}_{\mathbbm {f}'}, {\overline{q}}_{{\mathbb {F}}'}) \in H_{\mathbbm {f}'}$ s.t. $\mathbbm {f}({\overline{q}}_{\mathbbm {f}'}) \models _{{\mathbb {G}}({\overline{q}}_{{\mathbb {F}}'})} {\mathbb {F}}({\overline{q}}_{{\mathbb {F}}'})$. Thus, by definition of H, $(({\overline{q}}_{\mathbbm {f}}, {\overline{q}}_{\mathbbm {f}'}), ({\overline{q}}_{{\mathbb {F}}}, {\overline{q}}_{{\mathbb {F}}'})) \in H$. And, by by Proposition 3, ${\overline{q}}_{\mathbbm {f}} \otimes {\overline{q}}_{\mathbbm {f}'} \models _{{\mathbb {G}}_{{\mathbb {F}}, {\mathbb {F}}'}({\overline{q}}_{{\mathbb {F}}},{\overline{q}}_{{\mathbb {F}}'})} {\mathbb {F}}({\overline{q}}_{{\mathbb {F}}}) \otimes {\mathbb {F}}'({\overline{q}}_{{\mathbb {F}}'})$. Hence, $H$ is a simulation relation for $\mathbbm {f}\otimes \mathbbm {f}' \models _{{\mathbb {G}}'} {\mathbb {F}}\otimes {\mathbb {F}}'$. $\square$

Two stateful interfaces are compatible if the stateless interfaces defined by their initial states are compatible, i.e. ${\mathbb {F}}(\hat{q}) \sim {\mathbb {F}}'(\hat{q}')$. As we only keep states defined by compatible stateless information-flow interfaces during a stateful interface composition, the condition above effectively guarantees that all states reachable from the initial state are defined from compatible interfaces. By following a proof strategy analogous to the proof of Proposition 16, we can easily lift results proved for the stateless interfaces related to composition and compatibility. In particular, we can prove that compatibility is commutative, composition preserves well-formedness, and stateful interfaces support incremental design of systems.

#### Theorem 17

Let ${\mathbb {F}}$, ${\mathbb {F}}'$ and ${\mathbb {F}}''$ be stateful information-flow interfaces. If ${\mathbb {F}}\sim {\mathbb {F}}'$ and $({\mathbb {F}}\otimes {\mathbb {F}}') \sim {\mathbb {F}}''$, then ${\mathbb {F}}' \sim {\mathbb {F}}''$ and ${\mathbb {F}}\sim ({\mathbb {F}}' \otimes {\mathbb {F}}'')$.

#### Proof

By definition of compatibility between stateful information-flow interfaces, we only need to prove the following statement for the intial states $\hat{q}$, $\hat{q}'$ and $\hat{q}''$ of arbitrary interfaces ${\mathbb {F}}$, ${\mathbb {F}}'$ and ${\mathbb {F}}''$:

If $${\mathbb {F}}(\hat{q}) \sim {\mathbb {F}}'(\hat{q}')$$ and $$({\mathbb {F}}(\hat{q}) \otimes {\mathbb {F}}'(\hat{q}')) \sim {\mathbb {F}}''(\hat{q}'')$$,

then $${\mathbb {F}}'(\hat{q}') \sim {\mathbb {F}}''(\hat{q}'')$$ and $${\mathbb {F}}(\hat{q}) \sim ({\mathbb {F}}'(\hat{q}') \otimes {\mathbb {F}}''(\hat{q}''))$$.

Which follows from Theorem 8 for stateless information-flow interfaces. $\square$

The composition operation on stateful information-flow interfaces can be generalized to distinguish between compatible and incompatible transitions of interfaces when they are composed. Usually this is done by labeling transitions with letters from an alphabet, so that only transitions with the same letter can be synchronized. While necessary for practical modeling, we omit this technical generalization to allow the reader to focus on the novelty of our formalism, which is the ability to specify information-flow constraints at each state of an interface.

### Refinement and independent implementability

A stateful interface ${\mathbb {F}}_R$ refines ${\mathbb {F}}_A$, if all output steps of ${\mathbb {F}}_R$ can be simulated by ${\mathbb {F}}_A$, while all input steps of ${\mathbb {F}}_A$ can be simulated by ${\mathbb {F}}_R$. To formalize this definition, we first define functions that, for each state $q$ of a given stateful interface with a transition relation $\delta$, return the set with all states that can be reached in one step from $q$ using $\delta$. Our final goal is to compare assumptions and guarantees that can be reached from each state in different stateful interfaces. Thus, the computed set is, in fact, a set of states’ sets, with one state set for each reachable assumption, and reachable open- and closed-guarantee, effectively defining *input* and *output* steps, respectively.

#### Definition 16

Let ${\mathbb {F}}= (X,Y,Q, \hat{q}, \delta , {\mathbb {A}},{\mathbb {G}}, {\mathbb {P}})$ be an stateful information-flow interface. *Input steps* from a given state $q\in Q$ are defined as:

$$\begin{aligned} \delta ^{X}(q) = \{ \delta ^{X}(q, {\mathcal {A}}) \ \mid \ {\mathcal {A}}\subseteq Z\times X\} \text { with } \delta ^{X}(q, {\mathcal {A}}) = \{q' \in \delta (q) \ \mid \ {\mathbb {A}}(q') = {\mathcal {A}}\}. \end{aligned}$$

While *output steps* from a given state $q\in Q$ are defined as:

$$\begin{aligned} \begin{aligned} \delta ^{Y}(q)&= \{\delta ^{Y}(q, {\mathcal {G}}, {\mathcal {P}}) \ \mid \ {\mathcal {G}}\subseteq Z\times Y\text { and }{\mathcal {P}}\subseteq Z\times Y\}\\&\quad \text {with } \delta ^{Y}(q, {\mathcal {G}}, {\mathcal {P}}) = \{q' \in \delta (q) \ \mid \ {\mathbb {G}}(q') = {\mathcal {G}}\text { and }{\mathbb {P}}(q') = {\mathcal {P}}\}. \end{aligned} \end{aligned}$$

We define below refinement between stateful information-flow interfaces as an alternating refinement relation [25].

#### Definition 17

The stateful information-flow interface ${\mathbb {F}}_R = (X,Y,Q_R, \hat{q}_R, \delta _R,$ ${\mathbb {A}}_R,{\mathbb {G}}_R, {\mathbb {P}}_R)$ *refines* ${\mathbb {F}}_A = (X,Y,Q_A, \hat{q}_A, \delta _A, {\mathbb {A}}_A,{\mathbb {G}}_A, {\mathbb {P}}_A)$, written ${\mathbb {F}}_R \preceq {\mathbb {F}}_A$, iff there exists a relation $H\subseteq Q_R \times Q_A$ s.t. $(\hat{q}_R, \hat{q}_A)\in H$ and for all $(q_R, q_A) \in H$:

- $${\mathbb {F}}_R(q_R) \preceq {\mathbb {F}}_A(q_A)$$;
- for all set of states $O \in \delta ^{Y}_R(q_R)$, there exists ${O' \in \delta ^{Y}_A(q_A)}$ s.t. for all set of states $I' \in \delta ^{X}_A(q_A)$, there exists $I \in \delta ^{X}_R(q_R)$ s.t. $(O\cap I) \times (O'\cap I') \subseteq H$.

#### Example 10

**Fig. 13:**
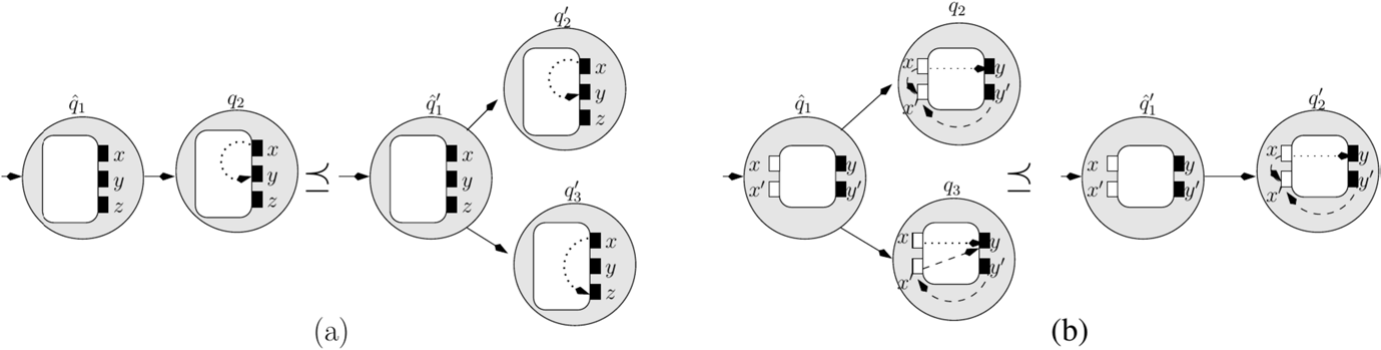
Refined interfaces with witness: (a) relation $\{(\hat{q}_1, \hat{q}_1'), (q_2, q_2')\}$; and (b) relation $\{(\hat{q}_1, \hat{q}_1'), (q_2, q_2'), (q_3, q_2')\}$

In Fig. 13 we depict two examples of refined stateful interfaces.

In Fig. 13(a) the stateless interface in each state only uses output ports and it only specifies closed-guarantees. The initial state of both stateful interfaces is the same, so they clearly refine each other. As there are no assumptions and open-guarantees, then, by Definition 17, we need to check that for all successors of the initial state in the refined interface $q_s$, there exists a successor of the initial state in the abstract interface $q'_s$ such that ${\mathbb {P}}_A(q'_s) \subseteq {\mathbb {P}}_R(q_s)$. This holds for the states $(q_2, q_2')$. Hence the relation $\{(\hat{q}_1, \hat{q}_1'), (q_2, q_2')\}$ witnesses the refinement. Note that the refined interface is obtained by removing a nondeterministic choice on the transition function.

The witness relation for the refinement depicted in Fig. 13(b) is $\{(\hat{q}_1, \hat{q}_1'),$ $(q_2, q_2'), (q_3, q_2')\}$. The initial states are the same, so the condition (i) in Definition 17 is trivially satisfied. The refined interface has two distinct *output transitions* from the initial state $\hat{q}_1$. It can either go to state $q_2$ by choosing the set of open-guarantees and proposition with only one element (*x*, *y*) or it can transition to state $q_3$ by committing to the set of no-flows $\{(x,y), (x',y)\}$ for the open-guarantees and $\{(x,y)\}$ as closed-guarantee. From the initial state of the abstract interface, there exists only one *input transition* possible, to assume that *x* does not flow to $x'$ and $y'$ does not flow to *x*. The following holds for both states accessible from the initial state in the refined interface: ${\mathbb {A}}_R(q_2) \subseteq {\mathbb {A}}_A(q'_2)$ and ${\mathbb {A}}_R(q_3) \subseteq {\mathbb {A}}_A(q'_2)$. The refined interface specifies an alternative transition from the initial state (represented by state $q_3$) that allows more environments while restricting the implementation and preserving the closed-guarantee.

#### Theorem 18

Let ${\mathbb {F}}_1 = (X_1,Y_1,Q_1, \hat{q}_1, \delta _1, {\mathbb {A}}_1,{\mathbb {G}}_1, {\mathbb {P}}_1)$ and ${\mathbb {F}}_2 = (X_2,Y_2,Q_2, \hat{q}_2, \delta _2,$ ${\mathbb {A}}_2,{\mathbb {G}}_2, {\mathbb {P}}_2)$ be stateful information-flow interfaces s.t. ${\mathbb {F}}_1 \preceq {\mathbb {F}}_2$. For all components $\mathbbm {f}$ and $\mathbbm {f}_{{\mathcal {E}}}$:

- If $$\mathbbm {f}\models _{{\mathbb {G}}_1} {\mathbb {F}}_1$$, then $$\mathbbm {f}\models _{{\mathbb {G}}_2} {\mathbb {F}}_2$$.
- If $$\mathbbm {f}_{{\mathcal {E}}} \models _{\!\!{\mathbb {A}}_2} {\mathbb {F}}_2$$, then $$\mathbbm {f}_{{\mathcal {E}}} \models _{\!\!{\mathbb {A}}_1} {\mathbb {F}}_1$$.

#### Proof

Assume that ${\mathbb {F}}_1 \preceq {\mathbb {F}}_2$. Then, there exists a simulation relation $H_{\preceq } \subseteq Q_1 \times Q_2$ that witnesses it.

For the item (a), assume that $\mathbbm {f}\models _{{\mathbb {G}}_1} {\mathbb {F}}_1$. Then, there exists a simulation relation $H_{\models _{}} \subseteq Q_\mathbbm {f}\times Q_1$ that witnesses it. Consider the relation $H= H_{\models _{}} \circ H_{\preceq }$. By definitions of refinement and implementation, $(\hat{q}_\mathbbm {f}, \hat{q}_1) \in H_{\models _{}}$ and $(\hat{q}_1, \hat{q}_2) \in H_{\preceq }$. So, $(\hat{q}_\mathbbm {f}, \hat{q}_2) \in H$. Additionally, ${\mathbb {F}}_1(\hat{q}_1) \preceq {\mathbb {F}}_2(\hat{q}_2)$ and $\mathbbm {f}(\hat{q}_\mathbbm {f}) \models _{{\mathbb {G}}_1(\hat{q}_1)} {\mathbb {F}}_1(\hat{q}_1)$. Then, $\mathbbm {f}(\hat{q}_\mathbbm {f}) \models _{{\mathbb {G}}_2(\hat{q}_2)} {\mathbb {F}}_2(\hat{q}_2)$. Consider arbitrary $(q_\mathbbm {f}, q_2) \in H$. By construction of $H$ there exists $(q_\mathbbm {f}, q_1) \in H_{\models _{}}$ and $(q_1, q_2) \in H_{\preceq }$. We want to prove that: $\text { if }q_\mathbbm {f}' \in \delta _\mathbbm {f}(q_\mathbbm {f}),\text { then }\text {there exists } q_{2}' \in \delta _{2}(q_{2}) \text { s.t.\ }(q_\mathbbm {f}', q_{2}') \in H\text { and }\mathbbm {f}(q_\mathbbm {f}') \models _{{\mathbb {G}}_2(q_2')} {\mathbb {F}}_2(q_2').$

Assume that $q_\mathbbm {f}' \in \delta _\mathbbm {f}(q_\mathbbm {f})$. By $(q_\mathbbm {f}, q_{1}) \in H_{\models _{}}$, then there exists a state $q_{1}' \in \delta _{1}(q_{1})$ s.t. $(q_\mathbbm {f}', q_{1}') \in H_{\models _{}}$. So, ${\mathbb {M}}(q_\mathbbm {f}') \subseteq \overline{{\mathbb {G}}}_{{\mathbb {F}}_1}(q_{1}')$. Additionally, by $(q_1, q_2) \in H_{\preceq }$ and $q_{1}' \in \delta _{1}(q_{1})$, there exists $q_{2}' \in \delta _{2}(q_{2})$ s.t. ${\mathbb {G}}_{{\mathbb {F}}_2}(q_{2}') \subseteq {\mathbb {G}}_{{\mathbb {F}}_1}(q_{1}')$. Thus, $(q_\mathbbm {f}', q_{2}') \in H$ and ${\mathbb {M}}(q_\mathbbm {f}') \subseteq \overline{{\mathbb {G}}_1}(q_1') \subseteq \overline{{\mathbb {G}}_2}(q_2')$. So, by definition of implements for stateless interfaces, $\mathbbm {f}(q_\mathbbm {f}') \models _{{\mathbb {G}}_2(q_2')} {\mathbb {F}}_2(q_2')$. Hence $H$ witnesses $\mathbbm {f}\models _{{\mathbb {G}}} {\mathbb {F}}$.

For the second item, assume that $\mathbbm {f}_{{\mathcal {E}}} \models _{{\mathcal {A}}_2} {\mathbb {F}}_2$. Then, there exists a simulation relation $H_{\models _{}} \subseteq Q_2 \times Q_{{\mathcal {E}}}$ that witnesses it. Consider the relation $H= H_{\preceq } \circ H_{\models _{}}$. We can prove analogously to the previous case that *H* witnesses $\mathbbm {f}_{{\mathcal {E}}} \models _{\!\!{\mathbb {A}}_1} {\mathbb {F}}_1$. $\square$

We can now prove that stateful information-flow interface satisfies the independent implementation of systems.

#### Theorem 19

For all well-formed interfaces ${\mathbb {F}}_1'$, ${\mathbb {F}}_1$ and ${\mathbb {F}}_2$, if ${\mathbb {F}}_1' \preceq {\mathbb {F}}_1$ and ${\mathbb {F}}_1 \sim {\mathbb {F}}_2$, then ${\mathbb {F}}_1' \sim {\mathbb {F}}_2$ and ${\mathbb {F}}_1' \otimes {\mathbb {F}}_2 \preceq {\mathbb {F}}_1 \otimes {\mathbb {F}}_2$.

#### Proof

Assume that: (i)${\mathbb {F}}_1' \preceq {\mathbb {F}}_1$; and (ii) ${\mathbb {F}}_1 \sim {\mathbb {F}}_2$. ${\mathbb {F}}_1' \sim {\mathbb {F}}_2$ follows from (i) and Theorem 10 for stateless interfaces. We prove now that ${\mathbb {F}}_1' \otimes {\mathbb {F}}_2 \preceq {\mathbb {F}}_1 \otimes {\mathbb {F}}_2$. From (i), there exists a relation $H_\preceq \subseteq Q_1' \times Q_1$ that witnesses the refinement. Consider the relation: $H= \{((q_{{\mathbb {F}}_1'}, q_{{\mathbb {F}}_2}),(q_{{\mathbb {F}}_1}, q_{{\mathbb {F}}_2})) \ \mid \ (q_{{\mathbb {F}}_1'},q_{{\mathbb {F}}_1}) \in H_\preceq \text { and }{\mathbb {F}}_1(q_{{\mathbb {F}}_1}) \sim {\mathbb {F}}_2(q_{{\mathbb {F}}_2}) \}.$

By (i) and (ii), $((\hat{q}_{{\mathbb {F}}_1'}, \hat{q}_{{\mathbb {F}}_2}),(\hat{q}_{{\mathbb {F}}_1}, \hat{q}_{{\mathbb {F}}_2})) \in H$. Additionally, ${\mathbb {F}}_1'(\hat{q}_{{\mathbb {F}}_1'}) \preceq {\mathbb {F}}_1(\hat{q}_{{\mathbb {F}}_1})$. Then, by Theorem 10 for stateless interface, ${\mathbb {F}}_1'(\hat{q}_{{\mathbb {F}}_1'}) \otimes {\mathbb {F}}_2(\hat{q}_{{\mathbb {F}}_2}) \preceq {\mathbb {F}}_1(\hat{q}_{{\mathbb {F}}_1}) \otimes {\mathbb {F}}_2(\hat{q}_{{\mathbb {F}}_2})$.

Consider arbitrary $((q_{{\mathbb {F}}_1'}, q_{{\mathbb {F}}_2}),(q_{{\mathbb {F}}_1}, q_{{\mathbb {F}}_2})) \in H$ and arbitrary $O \in \delta ^{Y}_{{\mathbb {F}}_1' \otimes {\mathbb {F}}_2}((q_{{\mathbb {F}}_1'}, q_{{\mathbb {F}}_2}))$. Then, there exists ${\mathcal {G}}'$ and ${\mathcal {P}}'$ s.t. $O = \delta ^{Y}_{{\mathbb {F}}_1' \otimes {\mathbb {F}}_2}((q_{{\mathbb {F}}_1'}, q_{{\mathbb {F}}_2}), {\mathcal {G}}', {\mathcal {P}}')$. By $H_\preceq$ witnessing (i) and Definition 16, there exists ${\mathcal {G}}\subseteq {\mathcal {G}}'$ and ${\mathcal {P}}\subseteq {\mathcal {P}}'$ s.t. $O' = \delta ^{Y}_{{\mathbb {F}}_1 \otimes {\mathbb {F}}_2}((q_{{\mathbb {F}}_1}, q_{{\mathbb {F}}_2}), {\mathcal {G}}, {\mathcal {P}})$.

Consider arbitrary $I \in \delta ^{X}_{{\mathbb {F}}_1 \otimes {\mathbb {F}}_2}((q_{{\mathbb {F}}_1}, q_{{\mathbb {F}}_2}))$. Then, by $H_\preceq$ witnessing (i) and Definition 16, there exists ${\mathcal {A}}$ s.t. $I' = \delta ^{X}_{{\mathbb {F}}_1 \otimes {\mathbb {F}}_2}((q_{{\mathbb {F}}_1}, q_{{\mathbb {F}}_2}), {\mathcal {A}})$. By $H_\preceq$ witnessing (i), there exists ${\mathcal {A}}' \subseteq {\mathcal {A}}$ s.t. $I = \delta ^{X}_{{\mathbb {F}}_1' \otimes {\mathbb {F}}_2}((q_{{\mathbb {F}}_1'}, q_{{\mathbb {F}}_2}), {\mathcal {A}}')$.

Consider arbitrary $((q'_{{\mathbb {F}}_1'}, q'_{{\mathbb {F}}_2}),(q'_{{\mathbb {F}}_1}, q'_{{\mathbb {F}}_2})) \in (O \cap I) \times (O' \cap I')$. Then, by (i) and *H* definition, ${\mathbb {F}}_1'(q'_{{\mathbb {F}}_1'}) \preceq {\mathbb {F}}_1(q'_{{\mathbb {F}}_1})$ and ${\mathbb {F}}_1(q'_{{\mathbb {F}}_1}) \sim {\mathbb {F}}_2(q'_{{\mathbb {F}}_2})$ So, by Theorem 10 for stateless interfaces, ${\mathbb {F}}_1'(q_1') \otimes {\mathbb {F}}_2(q_2) \preceq {\mathbb {F}}_1(q_1) \otimes {\mathbb {F}}_2(q_2)$.

Hence $H$ is a witness relation for ${\mathbb {F}}_1' \otimes {\mathbb {F}}_2 \preceq {\mathbb {F}}_1 \otimes {\mathbb {F}}_2$. $\square$

## Related work

To the best of our knowledge, we are the first to provide a theory for top-down and bottom-up design of information-flow system requirements that supports both incremental design and independent implementability of systems. The literature closest to our work about information-flow focus on the semantic aspects of it. The novelty of our work lies on explicit separation of the structural concerns from the semantic aspects of information-flow.

Language-based techniques have been proved useful to verify and enforce information flow policies [6]. Examples range from type systems [8] to program analysis using program-dependency graphs (PDGs) [7, 26]. In our approach we aim at composition and refinement notions that are independent of the language adopted for the implementations.

Information-flow properties can be specified with respect to the observed behavior of a system, in which each of its execution runs is abstracted as a trace. In this approach, properties often compare multiple executions of a system to certify that no forbidden flow can be deduced by an observer. Such properties over multiple execution traces are called hyperproperties [11]. Temporal logics [27], like LTL or CTL* are used to specify trace properties of reactive systems. HyperLTL and HyperCTL* [9] extend temporal logics by introducing quantifiers over path variables. They allow relating multiple executions and expressing information-flow security properties [9, 11]. Epistemic temporal logics (ETL) [28] provide the knowledge connective with an implicit quantification over traces. With ETL we can reason about the knowledge gain of agents over time. Then, we can specify which information can be learned by the agents while interacting with the system [29]. All these LTL extensions reason about closed systems while our approach allows compositional reasoning about open systems. Moreover, we focus here on the structural aspect of information-flow, and not yet on its semantic interpretation. Thus, all information-flow trace-based semantics are orthogonal to our approach.

Interface theories belong to the broader area of contract-based design [15], originally popularized by Meyer [30], following earlier ideas introduced by Floyd and Hoare [31, 32]. Our theory follows closely the philosophy for formal frameworks for systems design introduced for Interface automata (IA) [33] and Assume/Guarantee (A/G) [16] interfaces. Interface theories were later extended with extra-functional requirements such as resource [19], timing [20, 21] and security [34] requirements. Unlike in previous interface formalisms, we had to introduce the notion of *properties* which capture the intent of the designer and can be used to steer the refinement of interfaces.

*Interface for structure and security* (ISS) [34] is a variant of IA that enables specification of two types of actions on (1) low and (2) high confidential information. ISS uses a bisimulation-based notion of non-interference that checks whether the system behaves in the same way when high actions are performed or when they are considered hidden actions. Our approach is orthogonal to IA and their extensions: we do not characterise the type of actions of each component, but only their input/output ports, defining explicitly the information-flow relations between variables.

Our approach took inspiration from *relational interfaces* (RIs) [18]. RIs specify the legal inputs that the environment is allowed to provide to the component along with the legal outputs that the component can generate when provided with these input. RIs do not have assumptions and guarantees defined separately. Instead, they have a contract that specifies the desired input–output behavior. A contract in RIs is expressed over individual traces. Then, an RI contract can only relate input and output values in a trace, and not across multiple traces. This restricts considerably RIs expressivity concerning information-flow properties. Besides, RIs are trace-based interfaces, while in our approach we focus on the structural aspect of information-flow, which may change from state to state (in the stateful case). Our approach can be seen as a limited way to introduce relational properties into A/G interfaces, namely solely for guiding refinement. This limited way avoids many of the technical complexities of general relational interfaces [18].

Recently, Incer et al. [35] proposed hypercontracts as a meta-theory for assumption-guarantee contracts that supports the specification of hyperproperties. Hypercontracts are pairs of assumptions and guarantees of the close system, each defining a set of components. Guarantees of the open system are the quotient between guarantees of the closed system and the assumptions. Our contract theory is similar to hypercontracts in that our assumptions and guarantees also specify component sets. However, we decided to keep the closed system requirements at the interface level. By having both open- and closed- guarantees in the definition of our interfaces, we allow the designer to specify further assumptions and open-guarantees, even if they are not needed to support the closed-guarantee.

## Conclusion

We propose a novel interface theory to specify information-flow properties. Our framework includes both *stateless* and *stateful* interfaces and supports both incremental design and independent implementability. To achieve this, and unlike in previous interface formalisms, we introduce the notion of *closed-guarantees* which captures the intent of the designer for the interaction between assumptions and guarantees local to implementations (called open-guarantees). Moreover, closed-guarantees can be used to steer the refinement of interfaces. We provide a semantic interpretation of information-flow interfaces in terms of information-flow contracts, in which assumptions and guarantees are represented as sets of flow relations. The addition of the semantic view to the information-flow interfaces closes the gap between abstract modelling steps during concept design and the actual implementation of the components.

As future work, we will explore how to extend our theory with sets of *must-flows*, i.e. support for modal specifications [36]. This will enable, for example, to specify flows that a state $q$ must implement so that the system can transition to a different state, which is useful to specify declassification of information. Another interesting direction will be to study the introduction of such design-guiding closed-guarantees in the context of other interface languages.

## Data Availability

This manuscript has no associated data.

## Appendix: Proofs

### Stateless Information-flow Interfaces

#### Lemma 6

Let $F$, $F'$ and $F''$ be interfaces with pairwise disjoint set of output variables. Then, ${\mathcal {G}}_{F\otimes (F' \otimes F'')} = {\mathcal {G}}_{(F\otimes F')\otimes F''}$.

#### Proof

Consider arbitrary interfaces $F$, $F'$ and $F''$ with pairwise disjoint set of output variables. By definition of variables between different interfaces:

$$\begin{aligned} Z_{F\otimes F', F''} \times Y_{F\otimes F', F''} = (Z_{F} \cup Z_{F'} \cup Z_{F''}) \times (Y_{F} \cup Y_{F'} \cup Y_{F''}) = Z_{F, F' \otimes F''} \times Y_{F, F' \otimes F''}. \end{aligned}$$

In what follows, we denote the set of all variables over the three interfaces as $Z$ (i.e., $Z= Z_{F} \cup Z_{F'} \cup Z_{F''}$), and the set of output variables as $Y$ (i.e., $Y= Y_{F} \cup Y_{F'} \cup Y_{F''}$). By definition of composite open-guarantees:

$$\begin{aligned} \begin{aligned} {\mathcal {G}}_{(F\otimes F')\otimes F''}&= (Z\times Y) \setminus ({\mathcal {G}}_{F\otimes F'} \bullet {\mathcal {G}}_{F''}) \text { and }\\ {\mathcal {G}}_{F\otimes (F' \otimes F'')}&= (Z\times Y) \setminus ({\mathcal {G}}_{F} \bullet {\mathcal {G}}_{F' \otimes F''}). \end{aligned} \end{aligned}$$

Then, what we want to prove is equivalent to proving:

$$\begin{aligned} {\mathcal {G}}_{F\otimes F'} \bullet {\mathcal {G}}_{F''} = {\mathcal {G}}_{F} \bullet {\mathcal {G}}_{F' \otimes F''}. \end{aligned}$$

We start by proving that ${\mathcal {G}}_{F\otimes F'} \bullet {\mathcal {G}}_{F''} \subseteq {\mathcal {G}}_{F} \bullet {\mathcal {G}}_{F' \otimes F''}$. By Lemma 2, this is equivalent to prove that for all pair of variables $(z,y) \in Z\times Y$ and all $n\in {\mathbb {N}}$:

$$\begin{aligned} \text { if }&(z,y) \in (\textrm{Id}_{Z} \cup \overline{{\mathcal {G}}}_{F''}) \circ ( \overline{{\mathcal {G}}}_{F\otimes F'} \circ \overline{{\mathcal {G}}}_{F''})^n \circ (\textrm{Id}_{Y} \cup \overline{{\mathcal {G}}}_{F\otimes F'}),\\ \text {there exists}\ m \in {\mathbb {N}}\text { s.t.\ }&(z,y) \in (\textrm{Id}_{Z} \cup \overline{{\mathcal {G}}}_{F' \otimes F''}) \circ (\overline{{\mathcal {G}}}_{F} \circ \overline{{\mathcal {G}}}_{F' \otimes F''})^m \circ (\textrm{Id}_{Y} \cup \overline{{\mathcal {G}}}_{F}). \end{aligned}$$

To simplify the presentation of the proof, we start by proving for the case that $y \in Y_{F''}$. Note that, if $y \in Y_{F''}$, by output variables being disjoint, then, for all variables $z'\in Z$, $(z',y) \notin (\overline{{\mathcal {G}}}_{F\otimes F'} \cup \overline{{\mathcal {G}}}_{F})$. Moreover, $\overline{{\mathcal {G}}}_{F''} \circ \textrm{Id}_{Y_{F''}} = \overline{{\mathcal {G}}}_{F''}$. Then, we want to prove by induction on $n\in {\mathbb {N}}$ that:

$$\begin{aligned} \text { if }&(z,y) \in (\textrm{Id}_{Z} \cup \overline{{\mathcal {G}}}_{F''}) \circ ( \overline{{\mathcal {G}}}_{F\otimes F'} \circ \overline{{\mathcal {G}}}_{F''})^n,\\ \text {there exists}\ m \in {\mathbb {N}}\text { s.t.\ }&(z,y) \in (\textrm{Id}_{Z} \cup \overline{{\mathcal {G}}}_{F' \otimes F''}) \circ (\overline{{\mathcal {G}}}_{F} \circ \overline{{\mathcal {G}}}_{F' \otimes F''})^m \circ \textrm{Id}_{Y_{F''}}. \end{aligned}$$

For the *base case,* $n=0$, we consider arbitrary $(z,y) \in (\textrm{Id}_{Z} \cup \overline{{\mathcal {G}}}_{F''})$. By monotonicity of composite open-guarantees $\overline{{\mathcal {G}}}_{F''} \subseteq \overline{{\mathcal {G}}}_{F' \otimes F''}$, and by $y \in Y_{F''}$, it follows $(z,y) \in (\textrm{Id}_{Z} \cup \overline{{\mathcal {G}}}_{F' \otimes F''})\circ \textrm{Id}_{Y_{F''}}$.

For the *induction step*, we assume as induction hypothesis (IH) that the property holds for *n*. Now, consider arbitrary (*z*, *y*) s.t. $(z,y) \in (\textrm{Id}_{Z} \cup \overline{{\mathcal {G}}}_{F''}) \circ ( \overline{{\mathcal {G}}}_{F\otimes F'} \circ \overline{{\mathcal {G}}}_{F''})^{n+1}$. Then, $(z,y) = \{(z,s)\}\circ \{(s,y)\}$ with:

$$\begin{aligned} (\star )\ (z,s) \in (\textrm{Id}_{Z} \cup \overline{{\mathcal {G}}}_{F''}) \circ ( \overline{{\mathcal {G}}}_{F\otimes F'} \circ \overline{{\mathcal {G}}}_{F''})^{n} \ \text { and }\ (s,y) \in \overline{{\mathcal {G}}}_{F\otimes F'} \circ \overline{{\mathcal {G}}}_{F''}. \end{aligned}$$

By induction hypothesis, exists $m\in {\mathbb {N}}$ s.t.:

$$\begin{aligned} (z,s)\in (\textrm{Id}_{Z} \cup \overline{{\mathcal {G}}}_{F' \otimes F''}) \circ (\overline{{\mathcal {G}}}_{F} \circ \overline{{\mathcal {G}}}_{F' \otimes F''})^m \circ \textrm{Id}_{Y_{F''}}. \end{aligned}$$

We proceed by cases on (*z*, *s*).

If $(z,s) \in \textrm{Id}_{Z} \circ \textrm{Id}_{Y_{F''}}$, then $(z,y) \in \textrm{Id}_{Y_{F''}} \circ \overline{{\mathcal {G}}}_{F\otimes F'} \circ \overline{{\mathcal {G}}}_{F''}$ and, more generally, $(z,y) \in \overline{{\mathcal {G}}}_{F\otimes F'} \circ \overline{{\mathcal {G}}}_{F''}$. By definition of composite open-guarantees, $(z,y) \in ({\mathcal {G}}_{F} \bullet {\mathcal {G}}_{F'}) \circ \overline{{\mathcal {G}}}_{F''}$. Then, by Lemma 2:

$$\begin{aligned} (z,y) \in (\textrm{Id}_{Z} \cup \overline{{\mathcal {G}}}_{F}) \circ (\overline{{\mathcal {G}}}_{F'} \circ \overline{{\mathcal {G}}}_{F})^* \circ (\textrm{Id}_{Y} \cup \overline{{\mathcal {G}}}_{F'}) \circ \overline{{\mathcal {G}}}_{F''}. \end{aligned}$$

By monotonicity of open-guarantees, $\overline{{\mathcal {G}}}_{F'} \subseteq \overline{{\mathcal {G}}}_{F' \otimes F''}$, $\overline{{\mathcal {G}}}_{F''} \subseteq \overline{{\mathcal {G}}}_{F' \otimes F''}$ and $\overline{{\mathcal {G}}}_{F'} \circ \overline{{\mathcal {G}}}_{F''} \subseteq \overline{{\mathcal {G}}}_{F' \otimes F''}$, so $(z,y) \in (\textrm{Id}_{} \cup \overline{{\mathcal {G}}}_{F}) \circ (\overline{{\mathcal {G}}}_{F' \otimes F''} \circ \overline{{\mathcal {G}}}_{F})^* \circ \overline{{\mathcal {G}}}_{F' \otimes F''}.$ Equivalently, $(z,y) \in (\textrm{Id}_{} \cup \overline{{\mathcal {G}}}_{F' \otimes F''}) \circ (\overline{{\mathcal {G}}}_{F} \circ \overline{{\mathcal {G}}}_{F' \otimes F''})^*.$

If $(z,s) \notin \textrm{Id}_{Z}$ then, by $(\star )$, $s \in Y_{F''}$. By $F'$ and $F''$ having disjoint sets of output variables, $\overline{{\mathcal {G}}}_{F' \otimes F''} = {\mathcal {G}}_{F'} \bullet {\mathcal {G}}_{F''}$ and Lemma 2:

$$\begin{aligned} (z,s) \in (\textrm{Id}_{Z} \cup \overline{{\mathcal {G}}}_{F' \otimes F''}) \circ (\overline{{\mathcal {G}}}_{F} \circ \overline{{\mathcal {G}}}_{F' \otimes F''})^{m-1} \circ \overline{{\mathcal {G}}}_{F} \circ (\textrm{Id}_{Z} \cup \overline{{\mathcal {G}}}_{F'}) \circ (\overline{{\mathcal {G}}}_{F''} \circ \overline{{\mathcal {G}}}_{F'})^* \circ \overline{{\mathcal {G}}}_{F''}. \end{aligned}$$

By $(\star )$, $\overline{{\mathcal {G}}}_{F\otimes F'} = {\mathcal {G}}_{F} \bullet {\mathcal {G}}_{F'}$ and Lemma 2:

$$\begin{aligned} (s,y) \in (\textrm{Id}_{Z} \cup \overline{{\mathcal {G}}}_{F}) \circ (\overline{{\mathcal {G}}}_{F'} \circ \overline{{\mathcal {G}}}_{F})^* \circ (\textrm{Id}_{Z} \cup \overline{{\mathcal {G}}}_{F'}) \circ \overline{{\mathcal {G}}}_{F''}. \end{aligned}$$

Then,

$$\begin{aligned} (z,y)&\in (\textrm{Id}_{Z} \cup \overline{{\mathcal {G}}}_{F' \otimes F''}) \circ (\overline{{\mathcal {G}}}_{F} \circ \overline{{\mathcal {G}}}_{F' \otimes F''})^{m-1}\!\! \circ \overline{{\mathcal {G}}}_{F} \circ (\textrm{Id}_{} \cup \overline{{\mathcal {G}}}_{F'}) \circ (\overline{{\mathcal {G}}}_{F''} \circ \overline{{\mathcal {G}}}_{F'})^*\!\! \circ \overline{{\mathcal {G}}}_{F''} \\&\quad \circ (\textrm{Id}_{Z} \cup \overline{{\mathcal {G}}}_{F}) \circ (\overline{{\mathcal {G}}}_{F'} \circ \overline{{\mathcal {G}}}_{F})^* \circ (\textrm{Id}_{} \cup \overline{{\mathcal {G}}}_{F'}) \circ \overline{{\mathcal {G}}}_{F''} \end{aligned}$$

Equivalently, $(z,y) \in (\textrm{Id}_{} \cup \overline{{\mathcal {G}}}_{F' \otimes F''}) \circ (\overline{{\mathcal {G}}}_{F} \circ \overline{{\mathcal {G}}}_{F' \otimes F''})^{m'}$ for some $m' > m$.

The case $y \in Y_{F,F'}$ is analogous. We prove by a similar inductive argument that ${\mathcal {G}}_{F\otimes F'} \bullet {\mathcal {G}}_{F''} \supseteq {\mathcal {G}}_{F} \bullet {\mathcal {G}}_{F' \otimes F''}$. $\square$

#### Lemma 7

Let $F$, $F'$ and $F''$ be information-flow interfaces that are pairwise composable.

- If $$(z,z') \in {\hat{\mathcal {A}}}_{F', F''}$$, then $$(z,z') \in {\hat{\mathcal {A}}}_{F\otimes F', F''}$$.
- If $$(z,z') \in {\hat{\mathcal {A}}}_{F, F' \otimes F''}$$, then $$(z,z') \in {\hat{\mathcal {A}}}_{F\otimes F', F''} \cup {\hat{\mathcal {A}}}_{F, F'}$$.

#### Proof

Consider arbitrary information-flow interfaces $F$, $F'$ and $F''$ that are pairwise composable, i.e., all three interfaces have pairwise disjoint sets of output variables.

We start by proving item (a). Consider arbitrary $(z,z') \in {\hat{\mathcal {A}}}_{F', F''}$. By definition of derived assumptions, there exists a variable *s* s.t.:

$$\begin{aligned} (z,s) \in {\mathcal {A}}_{F'} \cup {\mathcal {A}}_{F''} \text { and }(z',s) \in \overline{{\mathcal {G}}}_{F' \otimes F''}. \end{aligned}$$

Note that, by definition of interface and domain of composite open-guarantee, *s* is a shared variable, i.e., $s \in \textrm{Shared}_{F', F''}$, where $\textrm{Shared}_{F', F''} = (X_{F'} \cup X_{F''}) \cap (Y_{F'} \cup Y_{F''})$. Then, by $s\in \textrm{Shared}_{F', F''}$, $s\in Y_{F', F''}$ and, by output variables being disjoint, *s* cannot be an output variable of $F$ (i.e., $s\notin Y_{F}$). We proceed by cases on the domain of *s*. If $s \in Y_{F'}$, then, by $s \in X_{F'} \cup X_{F''}$ and definition of information-flow interfaces, *s* must an input variable of $F''$; hence, $(z,s) \in {\mathcal {A}}_{F''}$. By monotonicity, associativity (Lemma 6) and definition of of composite open-guarantee, $(z,'s) \in {\mathcal {G}}_{F\otimes F'} \bullet {\mathcal {G}}_{F''}$. So, by definition of derived assumptions, $(z,z') \in {\hat{\mathcal {A}}}_{F\otimes F', F''}$. If $s \in Y_{F''}$, then, by $s \in X_{F'} \cup X_{F''}$ and definition of information-flow interfaces, *s* must an input variable of $F'$ and $(z,s) \in {\mathcal {A}}_{F'}$. As for the previous case, it follows that $(z',s) \in {\mathcal {G}}_{F\otimes F'} \bullet {\mathcal {G}}_{F''}$ and consequently $(z,z') \in {\hat{\mathcal {A}}}_{F\otimes F', F''}$.

We now prove item (b). Assume that $(z,z') \in {\hat{\mathcal {A}}}_{F, F' \otimes F''}$. By definition of derived assumptions, there exists a variable *s* s.t.:

$$\begin{aligned} (z,s) \in {\mathcal {A}}_{F} \cup {\mathcal {A}}_{F' \otimes F''} \text { and }(z',s) \in \overline{{\mathcal {G}}}_{F, F' \otimes F''}, \text { where } s \in \textrm{Shared}_{F, F' \otimes F''}. \end{aligned}$$

By $(z',s) \in \overline{{\mathcal {G}}}_{F\otimes (F' \otimes F'')}$ and associativity of composite open-guarantees (Lemma 6), it follows (b1) $(z',s) \in \overline{{\mathcal {G}}}_{(F\otimes F') \otimes F''}$. We proceed now by cases on the domain of the shared variable $s\in (X_{F} \cup X_{F' \otimes F''}) \cap Y_{F, F' \otimes F''}.$

If $s\in X_{F} \cap Y_{F''}$, by all three interfaces having disjoint sets of output variables, then $s\notin Y_{F'}$, $s \in X_{F, F'}$ and *s* is a shared variable between $F\otimes F'$ and $F''$. Moreover, by $(z,s) \in {\mathcal {A}}_{F} \cup {\mathcal {A}}_{F' \otimes F''}$ and $s \in Y_{F''}$, then $s\notin X_{F' \otimes F''}$ and the only relevant case for (*z*, *s*) is it being in the assumption of $F$, i.e., $(z,s) \in {\mathcal {A}}_{F\otimes F'}$. Then, by (b1) and definition of derived assumptions, $(z,z') \in {\hat{\mathcal {A}}}_{F\otimes F', F''}$.

If $s\in X_{F} \cap Y_{F'}$, then, *s* is a shared variable between $F$ and $F'$. By $s\in Y_{F'}$, then $s\notin X_{F' \otimes F''}$. Then, we can assume (*z*, *s*) to be in the assumption of $F$, i.e., $(z,s) \in {\mathcal {A}}_{F}$. By associativity of composite open-guarantees, $(z',s) \in {\mathcal {G}}_{F\otimes F'} \bullet {\mathcal {G}}_{F''}$. By output variables of all three interfaces being disjoint, $s\in Y_{F'}$ and Lemma 2, then the last flow from any path from $z'$ to *s* must be in $\overline{{\mathcal {G}}}_{F\otimes F'}$. Formally, $(z',s) = (\textrm{Id}_{Z_{F\otimes F', F''}} \cup \overline{{\mathcal {G}}}_{F''}) \circ (\overline{{\mathcal {G}}}_{F\otimes F'} \circ \overline{{\mathcal {G}}}_{F''})^* \circ \overline{{\mathcal {G}}}_{F\otimes F'}$. Then, there exists a variable $s'$ s.t. $(z',s) = (z',s') \cdot (s',s)$ with:

$$\begin{aligned} (z',s') \in (\textrm{Id}_{Z_{F\otimes F', F''}} \cup \overline{{\mathcal {G}}}_{F''}) \circ (\overline{{\mathcal {G}}}_{F\otimes F'} \circ \overline{{\mathcal {G}}}_{F''})^* \text { and }(s',s) \in \overline{{\mathcal {G}}}_{F\otimes F'}. \end{aligned}$$

By $(z,s) \in {\mathcal {A}}_{F}$, $s\in \textrm{Shared}_{F, F'}$ and $(s',s) \in \overline{{\mathcal {G}}}_{F\otimes F'}$, then $(z,s') \in {\hat{\mathcal {A}}}_{F, F'}$. If $(z',s') \in \textrm{Id}_{Z_{F\otimes F', F''}}$, then $(z',s) = (s',s)$. Hence $(z',s) \in \overline{{\mathcal {G}}}_{F\otimes F'}$ and, by $(z,s) \in {\mathcal {A}}_{F}$, $(z,z') \in {\hat{\mathcal {A}}}_{F, F'}$. Otherwise, $s'$ must be an output variable of $F''$ ($s' \in Y_{F''}$) and an input variable of the other interface ($s' \in X_{F\otimes F'}$). Then, $(z,s') \in {\mathcal {A}}_{F\otimes F'}$. By $(z',s') \in \overline{{\mathcal {G}}}_{(F\otimes F') \otimes F''}$ and definition of derived assumptions, $(z,z') \in {\hat{\mathcal {A}}}_{F\otimes F', F''}$.

If $s \in X_{F' \otimes F''} \cap Y_{F}$, then it can only be the case that $(z,s) \in {\mathcal {A}}_{F' \otimes F''}$ and we proceed by cases on ${\mathcal {A}}_{F' \otimes F''}$ definition. If $(z,s) \in {\mathcal {A}}_{F'}$, then $s\in X_{F'} \cap Y_{F}$. The rest is analogous to the previous case where $s\in X_{F} \cap Y_{F'}$. If $(z,s) \in {\mathcal {A}}_{F''}$, then, by $(z',s) \in \overline{{\mathcal {G}}}_{F\otimes F', F''}$ and definition of derived assumptions, $(z,z') \in {\hat{\mathcal {A}}}_{F\otimes F', F''}$. Lastly, if $(z,s) \in {\hat{\mathcal {A}}}_{F', F''}$, by definition of derived assumptions:

$$\begin{aligned} (z,s') \in {\mathcal {A}}_{F'} \cup {\mathcal {A}}_{ F''} \text { and }(s,s') \in \overline{{\mathcal {G}}}_{F', F''}, \end{aligned}$$

with $s' \in \textrm{Shared}_{F', F''}$. By *s* being an output variable of $F$, $(z',s) \in \overline{{\mathcal {G}}}_{F, F' \otimes F''}$ and Lemma 2, $(z',s) \in (\textrm{Id}_{Z_{F, F' \otimes F''}} \cup \overline{{\mathcal {G}}}_{F' \otimes F''}) \circ (\overline{{\mathcal {G}}}_{F} \circ \overline{{\mathcal {G}}}_{F' \otimes F''})^* \circ \overline{{\mathcal {G}}}_{F}$. As $(s,s') \in \overline{{\mathcal {G}}}_{F', F''}$ then we know that a flow from *s* to $s'$ can be defined by an alternating composition of elements of $\overline{{\mathcal {G}}}_{F'}$ and $\overline{{\mathcal {G}}}_{F''}$, i.e. without using elements of $\overline{{\mathcal {G}}}_{F}$. Hence $(z',s) \cdot (s,s') \in \overline{{\mathcal {G}}}_{F, F' \otimes F''}$ and, by associativity of composite open-guarantees (Lemma 6), $(z',s') \in \overline{{\mathcal {G}}}_{F\otimes F', F''}$. If $(z,s') \in {\mathcal {A}}_{F'}$, then $s' \in Y_{F''}$ and, by (ii), $s' \notin Y_{F}$. Then, $s' \in X_{F\otimes F'}$ and so $(z,s') \in {\mathcal {A}}_{F\otimes F'}$. Hence, by $(z',s') \in \overline{{\mathcal {G}}}_{F\otimes F', F''}$ and $(z,s') \in {\mathcal {A}}_{F\otimes F'}$, $(z,z') \in {\hat{\mathcal {A}}}_{F\otimes F', F''}$. If $(z,s') \in {\mathcal {A}}_{F''}$, then, by $(z,s') \in \overline{{\mathcal {G}}}_{F\otimes F', F''}$, $(z,z') \in {\hat{\mathcal {A}}}_{F\otimes F', F''}$. $\square$

## References

[1] Bartocci E, Ferrère T, Henzinger TA, Nickovic D, da Costa AO (2022) Information-flow interfaces. In: Proceedings of FASE 2022: the 25th international conference on fundamental approaches to software engineering. LNCS, vol 13241. pp 3–22. 10.1007/978-3-030-99429-7

[2] Ratasich D, Khalid F, Geissler F, Grosu R, Shafique M, Bartocci E (2019) A roadmap toward the resilient internet of things for cyber-physical systems. IEEE Access 7:13260–13283. 10.1109/ACCESS.2019.2891969

[3] Mantel H (2002) On the composition of secure systems. In: IEEE Symposium on security and privacy, pp 88–101. 10.1109/SECPRI.2002.1004364

[4] Mantel H, Sands D, Sudbrock H (2011) Assumptions and guarantees for compositional noninterference. In: IEEE Computer security foundations symposium (CSF), pp 218–232. 10.1109/CSF.2011.22

[5] Schneider FB (2000) Enforceable security policies. ACM Trans Inform Syst Secur 3(1):30–50. 10.1145/353323.353382

[6] Sabelfeld A, Myers AC (2003) Language-based information-flow security. IEEE J Sel Areas Commun 21(1):5–19. 10.1109/JSAC.2002.806121

[7] Hammer C, Snelting G (2009) Flow-sensitive, context-sensitive, and object-sensitive information flow control based on program dependence graphs. Int J Inf Secur 8(6):399–422. 10.1007/s10207-009-0086-1

[8] Focardi R, Maffei M (2011) Types for security protocols. Formal Models Tech Anal Secur Protoc 5:143–181. 10.3233/978-1-60750-714-7-143

[9] Clarkson MR, Finkbeiner B, Koleini M, Micinski KK, Rabe MN, Sánchez C (2014) Temporal logics for hyperproperties. In: Principles of security and trust (POST). LNCS, vol 8414. pp 265–284. 10.1007/978-3-642-54792-8_15

[10] Mikulcak M, Herber P, Göthel T, Glesner S (2019) Information flow analysis of combined simulink/stateflow models. Inform Technol Control 48(2):299–315. 10.5755/j01.itc.48.2.21759

[11] Clarkson MR, Schneider FB (2010) Hyperproperties. J Comput Secur 18(6):1157–1210. 10.3233/JCS-2009-0393

[12] Hamilton MD, Tunstall M, Popovici EM, Marnane WP (2008) Side channel analysis of an automotive microprocessor. In: IET Irish signals and systems conference (ISSC), pp 4–9. 10.1049/cp:20080630

[13] Verdult R, Garcia FD, Balasch J (2012) Gone in 360 seconds: Hijacking with hitag2. In: 21st USENIX Security symposium, pp 237–252

[14] Benadjila R, Renard M, Lopes-Esteves J, Kasmi C (2017) One car, two frames: attacks on hitag-2 remote keyless entry systems revisited. In: 11th USENIX Workshop on offensive technologies

[15] Benveniste A, Caillaud B, Nickovic D, Passerone R, Raclet J, Reinkemeier P, Sangiovanni-Vincentelli AL, Damm W, Henzinger TA, Larsen KG (2018) Contracts for system design. Found Trends Electron Des Autom 12(2–3):124–400. 10.1561/1000000053

[16] de Alfaro L, Henzinger TA (2001) Interface theories for component-based design. In: Embedded software. LNCS, vol 2211. pp 148–165. 10.1007/3-540-45449-7_11

[17] de Alfaro L, Henzinger TA (2005) Interface-based design. In: Engineering theories of software intensive systems. NATO science series (Series II: mathematics, physics and chemistry), vol 195, pp 83–104. 10.1007/1-4020-3532-2_3

[18] Tripakis S, Lickly B, Henzinger TA, Lee EA (2011) A theory of synchronous relational interfaces. ACM Trans Progr Lang Syst TOPLAS 33(4):14. 10.1145/1985342.1985345

[19] Chakrabarti A, de Alfaro L, Henzinger TA, Stoelinga M (2003) Resource interfaces. In: Embedded software. LNCS, vol 2855. pp 117–133. 10.1007/978-3-540-45212-6_9

[20] de Alfaro L, Henzinger TA, Stoelinga M (2002) Timed interfaces. In: Embedded software. LNCS vol 2491. pp 108–122. 10.1007/3-540-45828-X_9

[21] David A, Larsen KG, Legay A, Nyman U, Wasowski A (2010) Timed I/O automata: a complete specification theory for real-time systems. In: Proceedings of the 13th ACM international conference on hybrid systems: computation and control (HSCC), pp 91–100. 10.1145/1755952.1755967

[22] Larsen KG, Nyman U, Wasowski A (2006) Interface input/output automata. In: Misra J, Nipkow T, Sekerinski E (eds) International symposium on formal methods (FM). LNCS, vol 4085, pp 82–97. 10.1007/11813040_7

[23] Lemke K, Sadeghi A-R, Stüble C (2005) An open approach for designing secure electronic immobilizers. In: Proceedings of ISPEC 2005. LNCS, vol 3439, pp 230–242. 10.1007/978-3-540-31979-5_20

[24] Benveniste A, Caillaud B, Ferrari A, Mangeruca L, Passerone R, Sofronis C (2008) Multiple viewpoint contract-based specification and design. In: Proceedings of FMCO 2007: the 6th International symposium on formal methods for components and objects. lecture notes in computer science, vol 5382. Springer, pp 200–225. 10.1007/978-3-540-92188-2_9

[25] Alur R, Henzinger TA, Kupferman O, Vardi MY (1998) Alternating refinement relations. In: CONCUR’98 Concurrency theory. LNCS, vol 1466. Springer, pp 163–178. 10.1007/BFb0055622

[26] Graf J, Hecker M, Mohr M (2013) Using JOANA for information flow control in Java programs—a practical guide. In: Software engineering 2013 - Workshopband. LNI, vol P-215, pp 123–138. https://dl.gi.de/20.500.12116/17361

[27] Pnueli A (1977) The temporal logic of programs. In: Annual symposium on foundations of computer science (FOCS), pp 46–57. 10.1109/SFCS.1977.32

[28] Bozzelli L, Maubert B, Pinchinat S (2015) Unifying hyper and epistemic temporal logics. In: Foundations of software science and computation structures (FoSSaCS). LNCS, vol 9034, pp 167–182. 10.1007/978-3-662-46678-0_11

[29] Balliu M, Dam M, Le Guernic G (2011) Epistemic temporal logic for information flow security. In: Proceedings of the ACM SIGPLAN 6th workshop on programming languages and analysis for security (PLAS), pp 1–12. 10.1145/2166956.2166962

[30] Meyer B (1992) Applying design by contract. Computer 25(10):40–51. 10.1109/2.161279

[31] Floyd RW (1967) Assigning meanings to programs. Proceed Symp Appl Math 19:19–32. 10.1007/978-94-011-1793-7_4

[32] Hoare CAR (1969) An axiomatic basis for computer programming. Commun ACM 12(10):576–580. 10.1145/363235.363259

[33] de Alfaro L, Henzinger TA (2001) Interface automata. In: European software engineering conference/foundations on software engineering (ESEC/FSE), pp 109–120. 10.1145/503209.503226

[34] Lee M, D’Argenio PR (2010) Describing secure interfaces with interface automata. Electron Notes Theor Comput Sci 264(1):107–123. 10.1016/j.entcs.2010.07.008

[35] Incer I, Benveniste A, Sangiovanni-Vincentelli AL, Seshia SA (2022) Hypercontracts. In: Proceedings of NFM 2022: the 14th International symposium. LNCS, vol 13260. pp 674–692. 10.1007/978-3-031-06773-0

[36] Raclet J-B, Badouel E, Benveniste A, Caillaud B, Legay A, Passerone R (2011) A modal interface theory for component-based design. Fund Inform 108(1–2):119–149. 10.3233/FI-2011-416

